# Strigolactones and sesquiterpene lactones induce *Orobanche cumana* germination via KAI2d receptors through distinct processes

**DOI:** 10.1111/tpj.70852

**Published:** 2026-04-16

**Authors:** Julien Affholder, Louis Lambret, Sara Penaranda, David Cornu, Philippe Lebris, Antoinette Keita, Sabine Delgrange, Stéphane Muños, Stéphane Téletchéa, François‐Didier Boyer, Jean‐Bernard Pouvreau, Philippe Delavault, Alexandre de Saint Germain

**Affiliations:** ^1^ Nantes Université, CNRS, US2B, UMR 6286 44000 Nantes France; ^2^ INRAE, AgroParisTech, Institute Jean‐Pierre Bourgin for Plant Sciences (IJPB) Université Paris‐Saclay 78000 Versailles France; ^3^ Institute for Integrative Biology of the Cell (I2BC), CEA, CNRS Université Paris‐Saclay 91198 Gif‐sur‐Yvette France; ^4^ CNRS, Institut de Chimie des Substances Naturelles, UPR 2301 Université Paris‐Saclay 91198 Gif‐sur‐Yvette France; ^5^ Laboratoire des Interactions Plantes‐Microbes‐Environnement (LIPME), INRAE, CNRS Université de Toulouse 31326 Castanet‐Tolosan France; ^6^ Present address: Institut de Recherche en Santé Digestive (IRSD) Université de Toulouse, INSERM, INRAE, ENVT Toulouse France

**Keywords:** broomrapes, *Orobanche cumana*, seed germination, KAI2d receptors, strigolactones, sesquiterpene lactones

## Abstract

*Orobanche cumana* is an obligate parasitic weed belonging to the Orobanchaceae family and represents the most important biotic constraint to sunflower seed production in all the regions where sunflower is cultivated, except in North and South America. *O. cumana* seeds compensate for their limited reserves by having strict requirements for germination, which involve the detection of specific allelochemicals exuded by sunflower roots, known as germination stimulants (GS). To date, these stimulants have been identified as strigolactones (SLs) and sesquiterpene lactones (SqTLs). In this study, we characterized nine KAI2d receptors of *O. cumana* with respect to their ability to perceive GS. We demonstrated that at least four OcuKAI2d proteins function as receptors for both canonical and non‐canonical SLs. In addition, we showed that these paralogs interact with the sunflower‐derived SqTL, (−)‐DCL, through a mechanism distinct from their interaction with SLs: they form a covalent adduct on a histidine residue within the binding pocket, adjacent to the serine of the catalytic triad. Our findings expand current knowledge on GS perception in *O. cumana* and offer promising perspectives for the development of sustainable and effective strategies for controlling this parasitic weed.

## Significance Statement

We characterized nine KAI2d receptors of *O. cumana* with respect to their ability to perceive two different families of germination stimulants: strigolactones and sesquiterpene lactones. We showed that these paralogs interact with the sunflower‐derived sesquiterpene lactones, dehydrocostuslactone, through a mechanism distinct from their interaction with strigolactones.

## INTRODUCTION

*Orobanche* and *Phelipanche* species (broomrapes) are obligate parasitic weeds belonging to the Orobanchaceae family that represent major threats to a range of economically important vegetables and crops, including legumes, sunflower, oilseed rape, tomato, and tobacco, across the Mediterranean basin and warm temperate regions of Europe, North Africa, the Middle East, and Asia (Gressel & Joel, 2013). Among these, the sunflower broomrape, *Orobanche cumana*, constitutes the most important biotic constraint to sunflower seed production in all the countries where sunflower is grown except in North and South America (Molinero‐Ruiz et al., 2015). Yield losses attributable to *O. cumana* typically exceed 50%, and can reach up to 100% in heavily infested fields (Shi & Zhao, 2020). Despite the deployment of several control strategies, none has led to unequivocal success (Fernandez‐Aparicio et al., 2022). Currently, the most efficient way of preventing expansion of sunflower broomrape involves the development of resistant cultivars that combine multiple resistance traits, implemented within an integrated pest management framework (Calderón‐González et al., 2022; Duriez et al., 2019; Fernandez‐Aparicio et al., 2022; Louarn et al., 2016; Pérez‐Vich et al., 2004; Pubert et al., 2024).

Sophisticated mechanisms enable the broomrapes to detect the presence of a suitable host plant and coordinate their life cycle with it (Brun et al., 2021). It is essentially the early stage of the parasite's development that is critical to its survival. A seedling that has germinated and fails to connect to a host will rapidly exhaust its limited energy reserves and die. Broomrapes compensate for these limited reserves by having strict requirements for germination, involving the presence of specific allelochemicals exuded by the host's roots, known as germination stimulants (GS). These chemical signals are mainly strigolactones (SLs) (Daignan‐Fornier et al., 2024). SLs are a class of plant hormones that regulate different aspects of plant growth and development (Waters et al., 2017). Over 40 SL molecules have been isolated from plants, classified as canonical and non‐canonical SLs (Daignan‐Fornier et al., 2024) (Figure S1a). The structural core of SLs is a tricyclic lactone (ABC part, canonical SLs) or an unclosed BC‐ring (non‐canonical SLs) connected via an enol ether bridge to an invariant α,β‐unsaturated furanone moiety (D‐ring). GR24 is a synthetic analog of canonical SLs and is widely used as a standard compound in laboratories working on SLs (Figure S1a) (de Saint Germain et al., 2019). To efficiently assess the receptor activity, many SL mimics have been synthesized over the years (Daignan‐Fornier et al., 2024) like the profluorescent probes Yoshimulactone Green [(±)‐YLG] or the coumarin‐based structure GC series (Figure S1b) (de Saint Germain et al., 2016; Tsuchiya et al., 2015).

The modalities of SL perception and the underlying signaling pathway have been mostly described in the model plant *Arabidopsis thaliana*. It is not SLs that trigger seed germination in *Arabidopsis*, but endogenous compounds [KAI2‐ligands (KLs)] that have yet to be identified, as well as exogenous smoke‐derived karrikin molecules (KARs) (Flematti et al., 2004; Nelson et al., 2011, 2012). However, SLs and KARs/KLs are perceived by the structurally similar α/β‐hydrolase receptors, DWARF14 (D14) and KARRIKIN INSENSITIVE2/HYPOSENSITIVE TO LIGHT (KAI2/HTL), respectively (Waters et al., 2012). Perception of SLs and KARs/KLs by their respective receptors causes their interaction with the F‐box protein MORE AXILLARY GROWTH2 (MAX2), which is a part of an E3 SCF ubiquitin ligase complex, causing rapid proteasome‐catalyzed degradation of SUPPRESSOR OF MAX2 1‐LIKE (SMXL) repressor proteins (Waters et al., 2017). Then, this event initiates a transcriptional response that affects various developmental and growth processes (Chang et al., 2025).

Interestingly, the seeds of obligate root parasitic plants of the Orobanchaceae family respond to infinitesimal doses of (±)‐GR24 (<pM concentrations) and are insensitive to KARs (Brun et al., 2019; Conn et al., 2015; de Saint Germain et al., 2019). Contrary to expectations, it is not D14 that is responsible for perceiving the SLs, but homologs of KAI2 (Conn et al., 2015; Toh et al., 2015; Tsuchiya et al., 2015). Moreover, SL perception is mediated not by a single KAI2 ortholog, but by a cocktail of paralogs whose number varies according to the broomrape species. Each broomrape species has retained a conserved copy orthologous to KAI2, called KAI2c (not involved in GS perception), and a cohort of SL divergent receptors called KAI2d (Nelson, 2021). As in *A. thaliana*, D14 is present in a single copy, while the *KAI2* gene has evolved, through duplication, into numerous copies of the *KAI2d* gene, leading to their neofunctionalization. According to the studies, the number of *KAI2d* sequences in *O. cumana* may vary from 6 to 9 (Bürger et al., 2025; Conn et al., 2015; Xu et al., 2022).

Sunflower roots exude heliolactone, a non‐canonical SL (Figure S1a), as well as other compounds of a different nature, such as sesquiterpene lactones (SqTLs) like dehydrocostus lactone (DCL) and costunolide (Figure S1c), both of which promote the germination of seeds of *O. cumana* but also *Orobanche minor* (*O. minor*) (Chabaud et al., 2022; Raupp & Spring, 2013; Takei et al., 2023; Ueno et al., 2014). The role of KAI2d proteins as receptors for canonical SLs has been clearly demonstrated in several broomrape and witchweed species such as *Phelipanche ramosa* (*P. ramosa*), *O. minor*, and *Striga hermonthica* (*S. hermonthica*) (Conn et al., 2015; de Saint Germain et al., 2021; Takei et al., 2023; Toh et al., 2015). However, very little is known about the mechanism of heliolactone and SqTLs perception by the seeds of *O. cumana*. Through differential scanning fluorometry (DSF) and isothermal titration calorimetry experiments, KAI2d3 and KAI2d4 from *O. minor* were shown to interact with SqTLs (Takei et al., 2023). However, these direct interactions are weaker than those observed with SLs. They also showed that costunolide weakly activates the thermoinhibited germination of *Arabidopsis kai2* mutant seeds expressing the exogenous OmKAI2d3 receptor. It should be noted that the effective concentrations of costunolide (100 μM) are much higher than those of GR24 (5 μM). In *O. cumana*, only the OcKAI2d2 protein (Genbank accession number MF358985) has been studied and shown to have a strong affinity for DCL (*K*_d_ = 2.4 μM) (Han et al., 2024). Using computational and LC–MS/MS analysis, the authors suggested that DCL can covalently bind surprisingly to OcKAI2d2 via two serine adducts. This mechanism has also been observed for DCL and the *Arabidopsis* KAI2 protein, although the activity on germination remains to be determined.

Depending on the *O. cumana* population, the perception of the GS cocktail in sunflower root exudates may differ (Chabaud et al., 2022; Dor et al., 2020; Joel et al., 2011). It is therefore possible to suppose that the difference in the induction of seed germination to GS may be the result of both diversity and polymorphism within KAI2d receptors. In this study, we characterized the KAI2 paralog receptors of *O. cumana* in their ability to perceive GS. We showed that at least four OcuKAI2d proteins act as both canonical and non‐canonical SL receptors. In addition, we demonstrated that these paralogs interact with the sunflower‐derived SqTL, (−)‐DCL, in a manner distinct from their interaction with SLs. Our results expand current knowledge on GS perception in *O. cumana*, offering perspectives for the development of sustainable and effective control methods against this parasitic weed.

## RESULTS

### *O. cumana* displays different affinities for natural and synthetic germination stimulants

*O. cumana* possesses the ability to recognize signaling molecules exuded by sunflower roots, which belong to two structurally distinct families: SLs and SqTLs. Despite their divergent core structures, both families share a conserved lactone functionality – specifically, a butenolide ring in SLs and an α‐methylene‐γ‐lactone moiety in SqTLs (Joel et al., 2011; Raupp & Spring, 2013; Ueno et al., 2014) (Figure S1).

**Figure 1:**
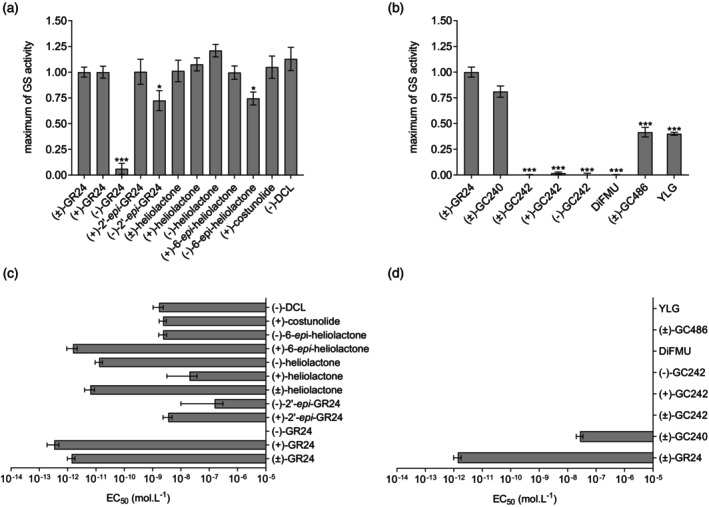
*O. cumana* germination is triggered by various natural GS, SL analogs, and profluorescent probes. (a) Maximum germination stimulation activity of SLs, SL analogs and SqTLs relative to 1 μM (±)‐GR24 (*N* = 6). (b) Maximum germination stimulation activity of various profluorescent probes relative to 1 μM (±)‐GR24 (*N* = 4). (c) Half maximal effective concentrations (EC_50_) of SLs, SL analogs, and SqTLs (*N* = 6). (d) EC_50_ of various profluorescent probes (*N* = 4). Compounds were assayed over a concentration range of 10^−13^ to 10^−6^ M in (a) and (c), 10^−16^ to 10^−5^ M in (b) and (d) (Figure S2). When the compounds induced germination and reached a plateau, the dose–response curves were modeled, and the maximum of GS activity (a, b) and EC50 (c, d) values were determined (Table S1). If GS activity was below 0.5, the maximum value was presented but EC_50_ was not modeled. Data are indicated ±SE. Each experiment was conducted at least three times. Statistical Welch's test with Holm correction was applied to compare the values to (±)‐GR24 (***P < 0.0001, *P < 0.01). Since most EC_50_ values were statistically different from (+/−)‐GR24, significant differences are only shown for maximum activity (a, b). The modeled curves and the data distribution can be seen in Figure S2.

First, we assessed the sensitivity of *O. cumana* seeds from the Spanish OcIN23 population to various compounds from both families, SLs and SqTLs, over a concentration range of 10^−13^ to 10^−6^ M. We further determined the maximum GS activity on seed germination and the half maximal effective concentration (EC_50_) if the GS activity was detected compared with mock (Figure 1a,c; Figure S2; Table S1). All tested compounds, except for (−)‐GR24, induced seed germination (Figure S2). (−)‐2′‐*epi*‐GR24 and (−)‐6‐*epi*‐heliolactone are the two other compounds that do not induce germination as high as (±)‐GR24 at maximum GS activity. Among the natural SLs, *O. cumana* perceives (−)‐heliolactone and (+)‐6*‐epi*‐heliolactone at the lowest concentration, with picomolar EC_50_ values comparable to the synthetic analog (+)‐GR24. By contrast, *O. cumana* seeds are less sensitive, in the nanomolar range, to SqTLs [(+)‐costunolide and (−)‐DCL] and non‐natural SLs [(+)‐2′‐*epi*‐GR24, (+)‐heliolactone, and (−)‐6‐*epi*‐heliolactone]. Notably, *O. cumana* was far less responsive to the orobanchol analog (−)‐2′‐*epi*‐GR24, with an EC_50_ ~10^6^‐fold higher than that of the strigol analog (+)‐GR24 (0.16 μM versus 0.34 pM) (Table S1).

We further evaluated *O. cumana* seed germination using profluorescent SL probes in which the ABC‐ring structure was substituted by a fluorescent moiety (Figure S1b) (de Saint Germain et al., 2016; Tsuchiya et al., 2015). These probes featured distinct D‐ring modifications: an unmodified D‐ring (GC240 and YLG), a D‐ring with either two additional methyl groups (GC242) or none (GC486), and in one case, complete absence of a D‐ring (DiFMU). GC242 probes and DiFMU did not induce seed germination at any tested concentration (Figure S2). In contrast, seed germination was moderately stimulated by (±)‐GC486 and (±)‐YLG, reaching about 40% of the maximum germination observed (Figure 1b). However, germination occurred only at 10 μM, which prevented the determination of EC_50_ values (Figure S2). Among the tested compounds, (±)‐GC240 elicited the strongest response, inducing up to 80% of maximum germination, not different from the (±)‐GR24. Nevertheless, *O. cumana* seeds were 10 000‐fold less sensitive to (±)‐GC240 (EC_50_ = 27.1 nM) than to (±)‐GR24 (EC_50_ = 1.4 pM) (Figure 1d; Table S1).

Overall, these results demonstrated that *O. cumana* seed germination is strongly dependent on the stereochemistry of the stimulating compound. Indeed, the lack of activity of (±)‐GC486, (±)‐GC242, and DiFMU confirms the requirement of an intact butenolide moiety for SL activity. The activity of SqTLs indicates that substitution of the methyl group with a vinylidene group can still be recognized by parasite seeds. Although the ABC‐ring is not essential for germination, its structural modifications substantially alter the sensitivity of *O. cumana* seeds.

### *O. cumana* genome contains at least 11 *KAI2/D14* paralogs

**Figure 2:**
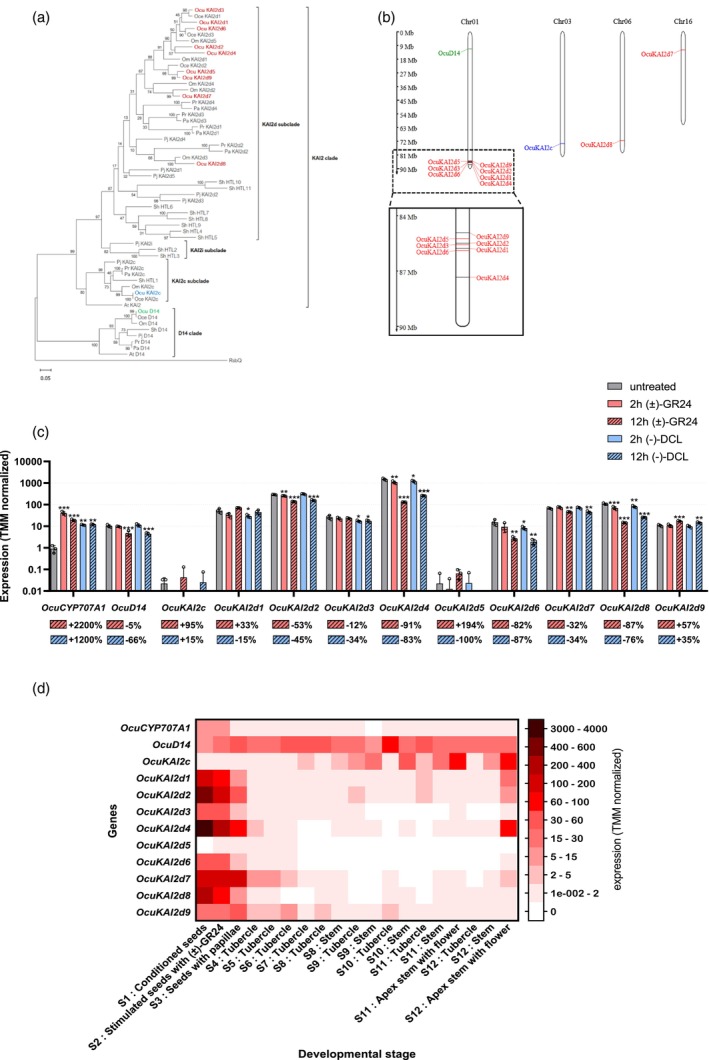
Identification of OcuKAI2 putative GS receptors in *O. cumana*. (a) Phylogenetic analysis of KAI2 and D14 amino acid sequences (At, *A. thaliana*; Oce, *O. cernua*; Ocu, *O. cumana*; Om, *O. minor*; Pa, *P. aegyptiaca*; Pj, *Phteirospermum japonicum*; Pr, *P. ramosa*; Sh, *Striga hermonthica*). The phylogenetic tree was constructed with the maximum likelihood method and 500 bootstrap replicates by means of RaxML. The scale bar represents 0.05 substitutions per site. Clades were designated as described by Conn et al. (2015). OcuKAI2c, OcuD14, and OcuKAI2d proteins are respectively displayed in blue, green, and red. (b) Schematic representation of *OcuKAI2* paralogs' positions on the *O. cumana* genome (Xu et al., 2022). Chromosomes that contain genes of interest are represented. *D14*, *KAI2c*, and *KAI2d* genes are represented in green, blue, and red, respectively. The area framed by a solid line is a zoom of the area framed by a dotted line. (c) Transcripts accumulation of *KAI2* and *D14* genes in *O. cumana* conditioned seeds. Seeds were either untreated, treated for 2 or 12 h with 1 μM (±)‐GR24 or (−)‐DCL. Means of read counts values normalized using the TMM (trimmed mean of *M* values) are shown with SD (*N* = 3). RNA was extracted from three independent seed batches for each condition. *OcuCYP707A1* was used as a control of germination induction. Because it is difficult to estimate the evolution of transcript accumulation due to the logarithmic scale, percentages of decrease or increase after 12 h of treatment compared with the untreated sample are provided under each gene label. Asterisks indicate statistical differences compared with untreated seed for each gene (one‐way ANOVA followed by Dunnett's post hoc test; *P < 0.05; **P < 0.01; and ***P < 0.001). (d) Heatmap showing the expression of *KAI2* and *D14* genes in *O. cumana* throughout its lifecycle. Values represent the mean of read counts values normalized using the TMM (trimmed mean of *M* values) from three independent samples at each stage. Sample numbers correspond to the chronological order of developmental stages (see Table S2). The color scale represents the expression levels from low to high.

Using the available *KAI2/D14* sequences (Conn et al., 2015; de Saint Germain et al., 2021; Toh et al., 2015), we investigated the presence of *KAI2* genes in the *O. cumana* annotated genome (GenBank accession JBFNAC000000000). We identified 11 distinct paralogous *KAI2/D14* coding genes, which were subsequently confirmed by SANGER sequencing from cDNA. Phylogenetic analysis using sequences from several *Orobanchaceae* species, together with comparison to sequences obtained from the genome and transcriptome of a Chinese *O. cumana* population (Xu et al., 2022), classified these genes into three clades: *D14*, *KAI2c*, and *KAI2d* (nine sequences) (Figure 2a; Figure S3). To avoid confusion with *Orobanche cernua* (*O. cernua*) sequences (*OceKAI2*) (Conn et al., 2015), the *O. cumana* sequences were designated *OcuD14*, *OcuKAI2c*, and *OcuKAI2d1–9*. As expected, all sequences clustered predominantly with *D14* and *KAI2* sequences, respectively, from *O. minor* and *O. cernua*, *OcuKAI2d8* displaying the most divergence from the *KAI2d* sequences. In a previous study, OmKAI2d3 and OmKAI2d4 from *O. minor* showed interactions with both SLs and SqTLs (Takei et al., 2023) and among the OcuKAI2ds, OcuKAI2d8, and OcuKAI2d7 are their closest homologs. All genes share a conserved structure, characterized by a single intron starting around 370 nucleotides downstream of the start codon as already described in *Striga asiatica* and other species from the Orobanchaceae (Conn et al., 2015; Yoshida et al., 2019) (Figure S4). Introns displayed various lengths with *OcuKAI2d8* showing the largest intronic region (~1900 nucleotides). Protein sequences were approximately 270 amino acids in length, except for OcuKAI2d4 from the OcIN23 genome, which displayed a 35‐amino‐acid N‐terminal extension (Figure S5). Although some other KAI2 receptors, such as KAI2‐L in *Physcomitrium patens*, also possess an N‐terminal extension (Lopez‐Obando et al., 2021), the OcuKAI2d4 extension displays different amino acid motifs and represents the first such extension reported among parasitic KAI2d genes. Nevertheless, the sequence from Xu et al. (2022) orthologous to OcuKAI2d4 (OcuC01G02752) was truncated at both C and N terminals and lacked the extension, suggesting that this receptor may not be functional. Indeed, the non‐synonymous to synonymous nucleotide substitutions ratio (Ka/Ks) between the two copies was 1.04, indicating neutral selection. On the contrary, all the other pairs of orthologous sequences had Ka/Ks values below 0.4, with OcuKAI2d1, *OcuKAI2d6*, *OcuKAI2d7*, and *OcuKAI2d8* fully conserved, suggesting that these paralogs may be under strong negative selection and thus play a similar role in both populations (Figure S3).

The genome assembly of the OcIN23 population from the Guadalquivir valley (Spain) revealed the presence of 19 chromosomes, in agreement with the genome published by Xu et al. (2022). The Xu's genome, exhibiting better assembly metrics, was selected as the reference for analyzing the genomic positioning of *KAI2* genes. In this assembly, *KAI2d* paralogs were identified on only three chromosomes, with most copies located on chromosome 1 (Figure 2b). Notably, seven *KAI2d* genes (*OcuKAI2d1–6* and *OcuKAI2d9*) were clustered within a compact region of chromosome 1 spanning less than 2.5 Mbp. These closely grouped paralogs, all belonging to the same subclade, showed a strong correlation between their chromosomal position and nucleotide sequence identity, suggesting that multiple tandem duplication events may have occurred through evolution (Figure S6).

The expression level of the *KAI2* genes was measured after 2 and 12 h post‐stimulation with both GS, (±)‐GR24, and (−)‐DCL (Figure 2c). In addition, their expression profiles were examined across different developmental stages and in various organs of *O. cumana* (Figure 2d). As expected, both GS, (−)‐DCL, and (±)‐GR24 triggered the upregulation of the *OcuCYP707A1* gene, the expression of which is induced by seed germination in obligate parasitic plants (Brun et al., 2019). In contrast, all receptor‐encoding genes, except *OcuKAI2d1* and *OcuKAI2d9*, were downregulated after 12 h of GS treatment, while *OcuKAI2d5* was not expressed. No significant differences in expression level were detected for any of the genes across the treatments. Most *KAI2d* sequences were strongly expressed following a 7 days conditioning period and almost exclusively during the seed stages, with *OcuKAI2d4* being the highest expressed paralog. By contrast, *OcuKAI2c* was not expressed in the seeds and *OcuD14* was expressed with very little variations throughout the parasite's life cycle, suggesting that they do not intervene in the host's early recognition.

Our results indicate that OcuKAI2d paralogs seem to be involved in the specialization to the sunflower‐derived SLs and SqTLs.

### 3D‐modeled *O. cumana* receptors display various pocket volumes and affinities toward ligands

**Figure 3:**
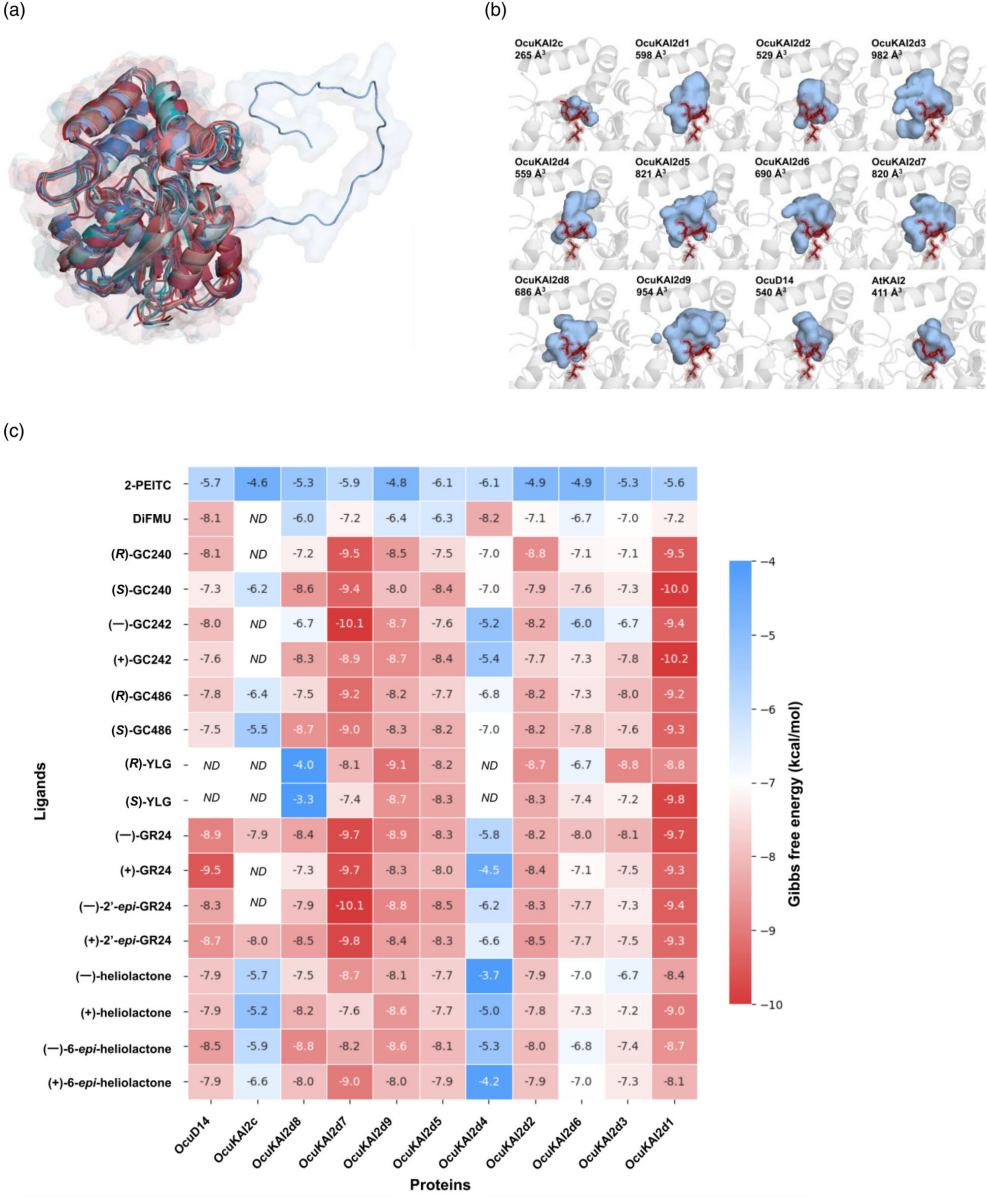
OcuKAI2d structures are in favor of an interaction with SL *in silico*. (a) Overlay of *O. cumana* KAI2 and D14 modeled structures. The blue extension belongs to OcuKAI2d4. (b) Visual representation of the ligand pockets of the *O. cumana* KAI2 paralogs with their respective volumes. The KAI2 proteins were modeled using Modeller v10.5 and the structures were visualized using PyMOL. The catalytic triad is highlighted in red. Volumes were measured using Castp30, pocket volume is determined using Connolly's solvent excluded volume (CSEV) measured in Å^3^. (c) Molecular docking of *O. cumana* KAI2/D14 paralogs against different compounds. Highest and lowest affinities are represented in red and blue, respectively. Affinities of molecules that could not reach the 4 Å area from the Ser of the catalytic triad were not determined (*ND*).

To predict the affinity of *O. cumana* KAI2 receptors toward their ligands, we first performed *in silico* analyses. Proteins in their open state were modeled using the crystal structure of OmKAI2d4 (Takei et al., 2023), ShHTL1 and ShD14 (Xu et al., 2018) as templates for OcuKAI2d proteins, OcuKAI2c, and OcuD14, respectively (Figure 3a). All paralog models were superimposed onto the crystallographic reference structure of OmKAI2d4 (Takei et al., 2023) and exhibited RMSD values between 0.7 and 0.9 Å, confirming a very similar overall fold, even though the proteins display divergences in their sequences (down to 42% identity between OcuKAI2d4 and OcuD14). Notably, the OcuKAI2d4 extension did not adopt any particular three‐dimensional fold. The binding pockets of OcuKAI2d proteins were larger than those of AtKAI2 (Guo et al., 2013) and OcuKAI2c, but displayed a wide range of volumes, from 529 to 982 Å^3^ (Figure 3b). We also retrieved the amino acids occupying the hydrophobic pocket and compared them to the crystal structures from AtD14 (Zhao et al., 2013) and AtKAI2 (Guo et al., 2013) (Figure S7). As expected, all proteins conserved the catalytic Ser97 and His247 residues (AtD14 positions), which were accessible to the solvent and therefore available for the interaction with potential GS ligands. Among the KAI2d receptors, OcuKAI2d8 exhibited the highest similarity to AtD14 and OcuKAI2d1 showed the lowest similarity; OcuKAI2d2 was the most divergent from AtKAI2. Recent studies demonstrated that the lack of bulky amino acids and the presence of polar residues in the KAI2d binding pocket favored the interaction of the ligand with the catalytic triad (Arellano‐Saab et al., 2021; Xu et al., 2018). Overall, all OcuKAI2d proteins possessed a higher proportion of small and polar amino acids in their binding pocket than AtKAI2 and AtD14 (Figure S7). However, OcuKAI2d4 proportionally conserved bulkier tyrosine and phenylalanine residues than the other proteins, resulting in a smaller binding pocket (Figure 3b; Figure S7) and leaving less space for potential ligands to access the catalytic triad.

Molecular docking of the tested compounds was performed to evaluate the ability of KAI2 models to accommodate their ligand in proximity to the catalytic triad, with a 4 Å threshold distance between the butenolide moiety of SL and the oxygen atom of the catalytic serine (Figure 3c). Most of the paralogs exhibited moderate to strong affinities for both natural SL and synthetic ligands, except for the 2‐PEITC used as a negative control, as expected, due to its inability to induce germination of *O. cumana* seeds (Auger et al., 2012). OcuKAI2d1 and OcuKAI2d7 showed robust docking values for all tested molecules, indicating a generic acceptability for ligand structure. OcuKAI2d4 and OcuKAI2c failed to properly accommodate sunflower‐derived SL in static docking analyses, suggesting that their natural ligands may not be SL. Confirmation of this hypothesis will require molecular dynamics analyses. Given the relatively good affinities of OcuKAI2d proteins for the diverse ligand structures, we could not further interpret a correlation between their interaction with the catalytic triad and their ability to induce germination. To summarize, all OcuKAI2d proteins, except for OcuKAI2d4, are in capacity to accept SL and to position them in a way that could promote interaction.

### OcuKAI2d proteins act as SL receptors

The above results raise the question of whether OcuKAI2d proteins have the ability to perceive canonical and non‐canonical SLs. To evaluate the potential function of *O. cumana* KAI2d proteins as SL receptors, we were able to heterologously express and purify 4 of the 9 OcuKAI2d proteins (OcuKAI2d2, OcuKAI2d6, OcuKAI2d7, and OcuKAI2d8) in *Escherichia coli* (Figure S8a,b). For each protein production batch, we performed mass spectrometry analysis to verify protein identity and detect any non‐specific post‐translational modifications. These analyses revealed that the protease inhibitor in the extraction buffer can generate a covalent glycerophosphate adduct on the serine of the catalytic triad of the OcuKAI2d2 protein, rendering the enzyme inactive and masking interactions with ligands (Figure S8c); therefore, the protease inhibitor was not used. We systematically removed the purification tag (GST or MBP) to avoid misinterpretation and false‐positive or false‐negative results. We noted that the presence of the MBP tag inhibits the enzymatic activity of the OcuKAI2d6 protein and masks the SL‐induced thermal shift (Figure S9).

**Figure 4:**
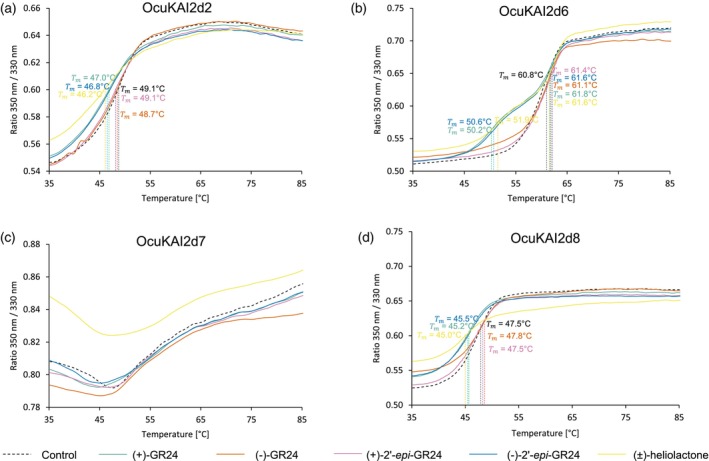
OcuKAI2d2, OcuKAI2d6, OcuKAI2d7, and OcuKAI2d8 show a stereoselectivity toward GR24 analogs mimicking the SL natural stereoconfigurations and perceive non‐canonical SLs. Thermostabilty (nanoDSF) of OcuKAI2d2 (a), OcuKAI2d6 (b), OcuKAI2d7 (c), and OcuKAI2d8 (d) at 10 μM with temperature in the absence of ligands (dotted black line) or in the presence of various ligands at 200 μM analyzed by monitoring the changes in fluorescence (ratio *F*_350nm_/*F*_330nm_). The apparent melting temperature (*T*_m_) is indicated for each curve. The traces represent one of the three replicates, and the experiments were repeated at least twice.

Interactions between the OcuKAI2d proteins and SL analogs were analyzed using differential scanning nano‐fluorimetry (nanoDSF). This approach evaluates protein–ligand interactions by detecting changes in tryptophan fluorescence (350 nm/330 nm ratio) and also detects conformational changes by recording shifts in the chemically induced melting temperature of proteins (de Saint Germain et al., 2021). Analysis of the initial fluorescence ratios revealed that the four GR24 stereoisomers and the non‐canonical SL, (±)‐heliolactone, interacted with all four OcuKAI2d proteins, with the exception of OcuKAI2d2 (Figure 4; Figure S10). For this protein, the stereoisomers (−)‐GR24 and (+)‐2′‐*epi*‐GR24 did not appear to interact, suggesting a stronger stereoselectivity toward SLs (Figure 4a). Furthermore, the high fluorescence ratio observed with heliolactone suggests that OcuKAI2d proteins had a stronger affinity for this ligand compared with GR24 analogs.

Similar to what has been demonstrated for the PrKAI2d3 protein of *P. ramosa* (de Saint Germain et al., 2021), only (±)‐heliolactone and the analogs of canonical SLs with natural configurations [(+)‐GR24 and (−)‐2′‐*epi*‐GR24], lowered the melting temperature of OcuKAI2d2, OcuKAI2d6, and OcuKAI2d8 proteins. This effect is consistent with ligand‐mediated protein destabilization, reflecting the transition of the SL receptor to an active signaling state (de Saint Germain et al., 2021; Yao et al., 2016, 2018) (Figure 4). For OcuKAI2d7, we were unable to evaluate the change in melting temperature in the presence of the ligand because the protein displayed an unconventional curve, similar to that observed for KAI2d3 in *Phtheirospermum japonicum* (Takei et al., 2024).

These results suggest that OcuKAI2d2, OcuKAI2d6, OcuKAI2d7, and OcuKAI2d8 act as SL receptors, corroborating the fact that *O. cumana* germination is induced indiscriminately by both canonical and non‐canonical SLs (Figure 1) (Fernández‐Aparicio et al., 2011; Ueno et al., 2014).

### OcuKAI2d enzymatic activity is correlated with SL signal transduction

**Figure 5:**
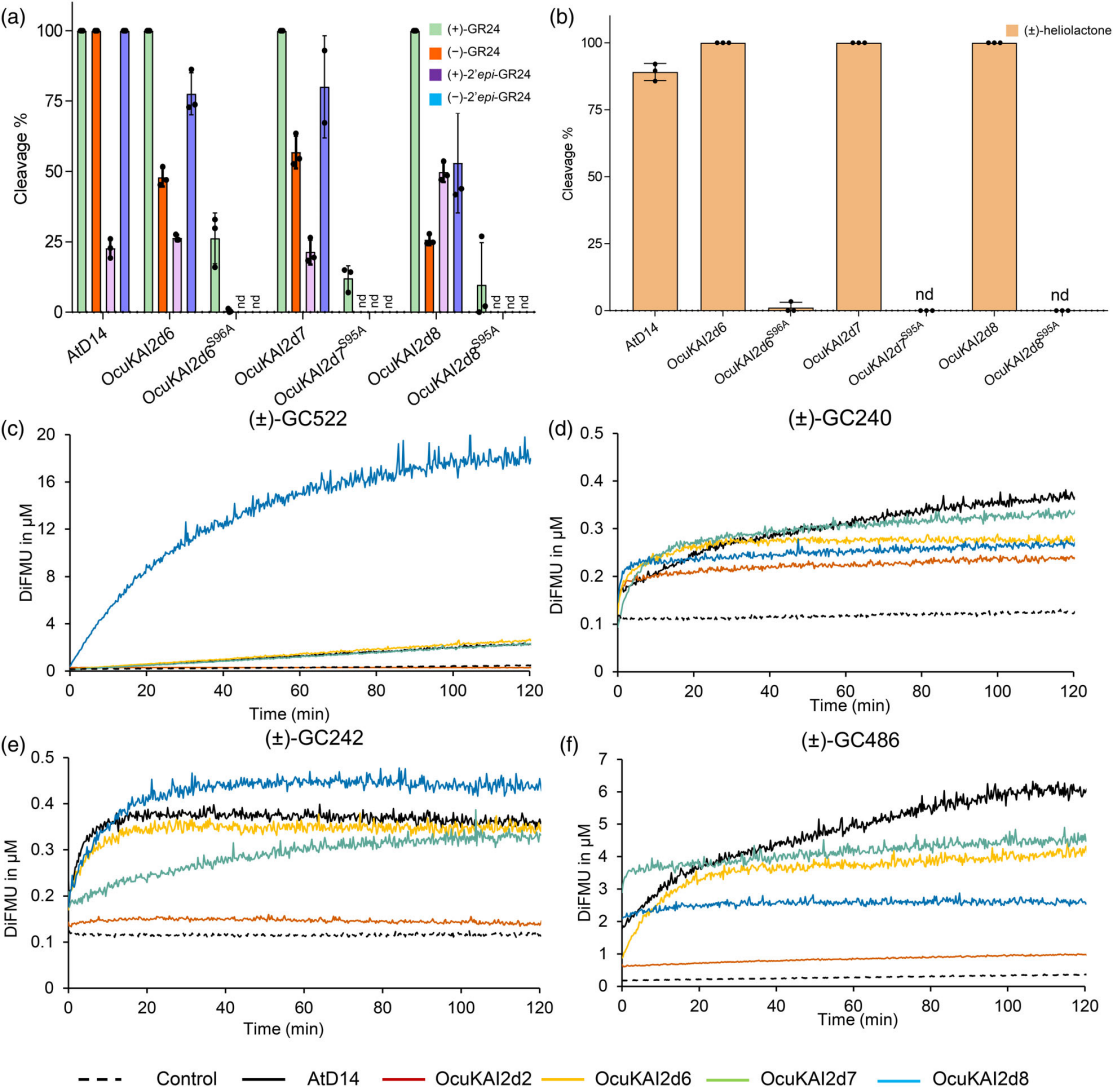
OcuKAI2d proteins show differential enzymatic activity regarding the SL analog structure. (a, b) Cleavage activity of GR24 isomers and heliolactone by various proteins. (+)‐GR24, (−)‐GR24, (+)‐2′*epi*‐GR24 and (−)‐2′*epi*‐GR24 (a) and (±)‐heliolactone (b) at 10 μM were incubated with OcuKAI2d6, OcuKAI2d7, OcuKAI2d8, OcuKAI2d6^S96A^, OcuKAI2d7^S95A^, OcuKAI2d8^S95A^, AtD14, and AtKAI2 at 5 μM for 150 min at 25°C. UPLC‐UV (260 nm) analysis was used to detect the remaining amount of GR24 isomers and heliolactone. Bars represent the mean value of the cleavage rate calculated from the remaining GR24 isomers and (±)‐heliolactone, taking into account the cleavage in the buffer alone (without protein sample), quantified with (±)‐1‐indanol as internal standard. Error bars represent the SD of three replicates (means ± SD, *N* = 3). nd, no cleavage detected. (c–f) Enzymatic kinetics for OcuKAI2d2, OcuKAI2d6, OcuKAI2d7, OcuKAI2d8, AtD14, and AtKAI2 proteins incubated with (±)‐GC522 (c), (±)‐GC240 (d), (±)‐GC242 (e) and (±)‐GC486 (f). Progress curves during hydrolysis of the probes, monitored (*λ*_em_ 460 nm) at 25°C with the use of 333 nM protein without compound (control) or 20 μM probes for (±)‐GC522 & (±)‐GC486 and 4 μM (±)‐GC240 & (±)‐GC242. The traces represent one of the three replicates, and the experiments were repeated at least twice.

According to several reports (de Saint Germain et al., 2021; Yao et al., 2017), KAI2 proteins show cleavage activity. Thus, the enzymatic activity of three OcuKAI2d was evaluated by incubating the proteins with GR24 analogs and (±)‐heliolactone, and the resulting reaction mixtures were subsequently analyzed by ultraperformance liquid chromatography‐diode array detector (UPLC‐DAD) analysis. We found that OcuKAI2d6/d7/d8 preferentially cleave (+)‐GR24 and (−)‐2′‐*epi*‐GR24 stereoisomers (Figure 5a). Their cleavage activity toward (±)‐heliolactone is even more efficient, reaching complete (100%) cleavage (Figure 5b). Mutation of the catalytic triad (OcuKAI2d^S96A^) abolished enzymatic activity for all tested substrates, with the exception of (+)‐GR24, for which residual activity is observed, suggesting a strong preference of the tested OcuKAI2d proteins for this molecule. Collectively, these results demonstrate that the tested OcuKAI2d proteins exhibit strong cleavage activity toward both the natural configuration‐mimicking GR24 stereoisomers and the non‐canonical SL (±)‐heliolactone.

To characterize the enzymatic properties of the OcuKAI2d proteins, we performed enzymatic kinetic analyses using profluorescent probes as substrates. We first used the GC522 probe (CoumarinAc) (de Saint Germain et al., 2022), a generic substrate for α/β‐hydrolases, to evaluate the cleavage activity of OcuKAI2. We next used (±)‐GC240 and (±)‐GC242, two SL‐mimicking molecules, to determine whether OcuKAI2 proteins perceive SLs in the same way as D14 hormone receptors and ShHTL and PrKAI2 proteins. We also tested GC486, a molecule similar to desmethyl‐YLG or desmethyl‐GR24, which are KL mimics that specifically stimulate KAI2c receptors (Figure S10). With GC522, we confirmed the enzymatic activity of OcuKAI2d6, OcuKAI2d7, and OcuKAI2d8 (Figure 5c). Among these, OcuKAI2d8 showed the strongest activity, displaying a clear Michaelian‐type cleavage kinetic (Figure 5c). Monitoring DiFMU fluorescence revealed that all tested OcuKAI2d proteins cleaved (±)‐GC240. We observed a biphasic fluorescence time course, consisting of a burst (initial) phase followed by a plateau phase (or a slow phase in the case of AtD14) (de Saint Germain et al., 2022). OcuKAI2d proteins thus appear to function as a single‐turnover enzyme toward the (±)‐GC240 probe, as the plateau never reached the maximum of the predicted product (20 μM) but instead produced a concentration product closer to the protein concentration (0.4 μM) (Figure 5d). This correlates with the ability of (±)‐GC240 to destabilize OcuKAI2d proteins (Figure S11) and to stimulate the germination of *O. cumana* seeds (Figure 1).

When we tested (±)‐GC242, we obtained curves similar to (±)‐GC240 for OcuKAI2d6, OcuKAI2d7, and OcuKAI2d8 but different for OcuKAI2d2, following the behavior of AtKAI2, which is unable to cleave this molecule (Figure 5e). This observation translates into a lack of destabilization of OcuKAI2d2 by (±)‐GC242, whereas OcuKAI2d6, OcuKAI2d7 and OcuKAI2d8 are destabilized (Figure S11). This could explain the lack of germination stimulation by (±)‐GC242 (Figure 1) and highlights the predominant role of OcuKAI2d2. With the (±)‐GC486 probe that lacks the 3′‐methyl group on the D‐ring, OcuKAI2d6, OcuKAI2d7, OcuKAI2d8, and AtD14 do not act as single‐turnover enzymes (de Saint Germain et al., 2016), unlike OcuKAI2d2 and AtKAI2 (Figure 5f), which seem able to trap the 3′‐desmethyl D‐ring, confirmed by nanoDSF assays (Figure S11a). The unique ability of OcuKAI2d2 to trap 3′‐desmethyl D‐ring may explain the moderate germination‐stimulating activity of (±)‐GC486 (Figure 1).

To test the hypothesis that OcuKAI2d proteins form a stable intermediate with the D‐ring, we incubated (±)‐GR24 and (±)‐heliolactone with these proteins and recorded mass spectrometry spectra under denaturing conditions. In all cases, a mass shift of 96 Da occurred, corresponding to the D‐ring covalently bound to the protein (Figure S12) and specifically attached to the histidine residue of the catalytic triad (Figure S13), similarly to what has been reported for RMS3 and PrKAI2d3 (de Saint Germain et al., 2016, 2021).

### OcuKAI2d proteins perceive DCL by covalent adduct formation to a histidine of the binding pocket

**Figure 6:**
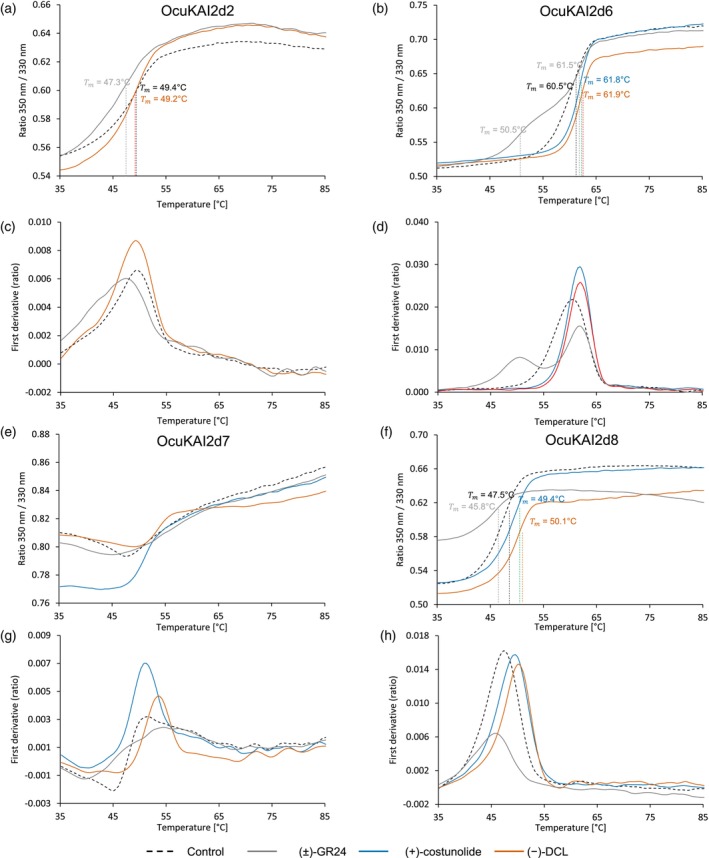
OcuKAI2d2, OcuKAI2d6, OcuKAI2d7, and OcuKAI2d8 interact with sesquiterpene lactones. Thermostabilty of OcuKAI2d2 (a, c), OcuKAI2d6 (b, d), OcuKAI2d7 (e, g) and OcuKAI2d8 (f, h) at 10 μM in the absence of ligands (dotted black line) or in the presence of (±)‐GR24 (grey line), (+)‐costunolide (blue line) or (−)‐DCL (orange line) at 200 μM analyzed by nanoDSF. The panels (a, b, e, f) show the changes in fluorescence (ratio *F*_350nm_/*F*_330nm_) with temperature and (c, d, g, h) the first derivatives for the *F*_350nm_/*F*_330nm_ curve against the temperature gradient from which the apparent melting temperature (*T*_m_) was determined for each sample. The traces represent one of the three replicates and the experiments were repeated at least twice.

As highlighted above, SqTLs, such as DCL and costunolide, can stimulate the germination of *O. cumana* seeds, making their interaction with sunflower unique (Joel et al., 2011). Therefore, the ability of OcuKAI2d proteins to perceive SqTLs was evaluated by nanoDSF and revealed that (+)‐costunolide and (−)‐DCL interact with the four tested OcuKAI2d proteins (Figure 6). While SLs lowered the melting temperature of OcuKAI2d proteins (a shift between −2 and −10°C depending on the protein), (−)‐DCL and (+)‐costunolide increased the melting temperature of OcuKAI2d6 and OcuKAI2d8 (a small shift of ≃+2.0°C), suggesting that SqTLs may induce a conformational change distinct from the SL‐induced destabilization and, *per se*, have a different perception mechanism. Puzzlingly, (−)‐DCL did not induce any changes in OcuKAI2d2, even though (−)‐DCL can bind to it (Figure 6a–c). We then investigated whether OcuKAI2d proteins can break down SqTLs. Despite a decrease in (−)‐DCL amount, we did not observe a degradation product of this chemical, as with GR24 or heliolactone (Figure S14). This suggests no cleavage activity with (−)‐DCL. The decrease in the amount of (−)‐DCL could be explained by ligand trapping by the OcKAI2d proteins.

**Figure 7:**
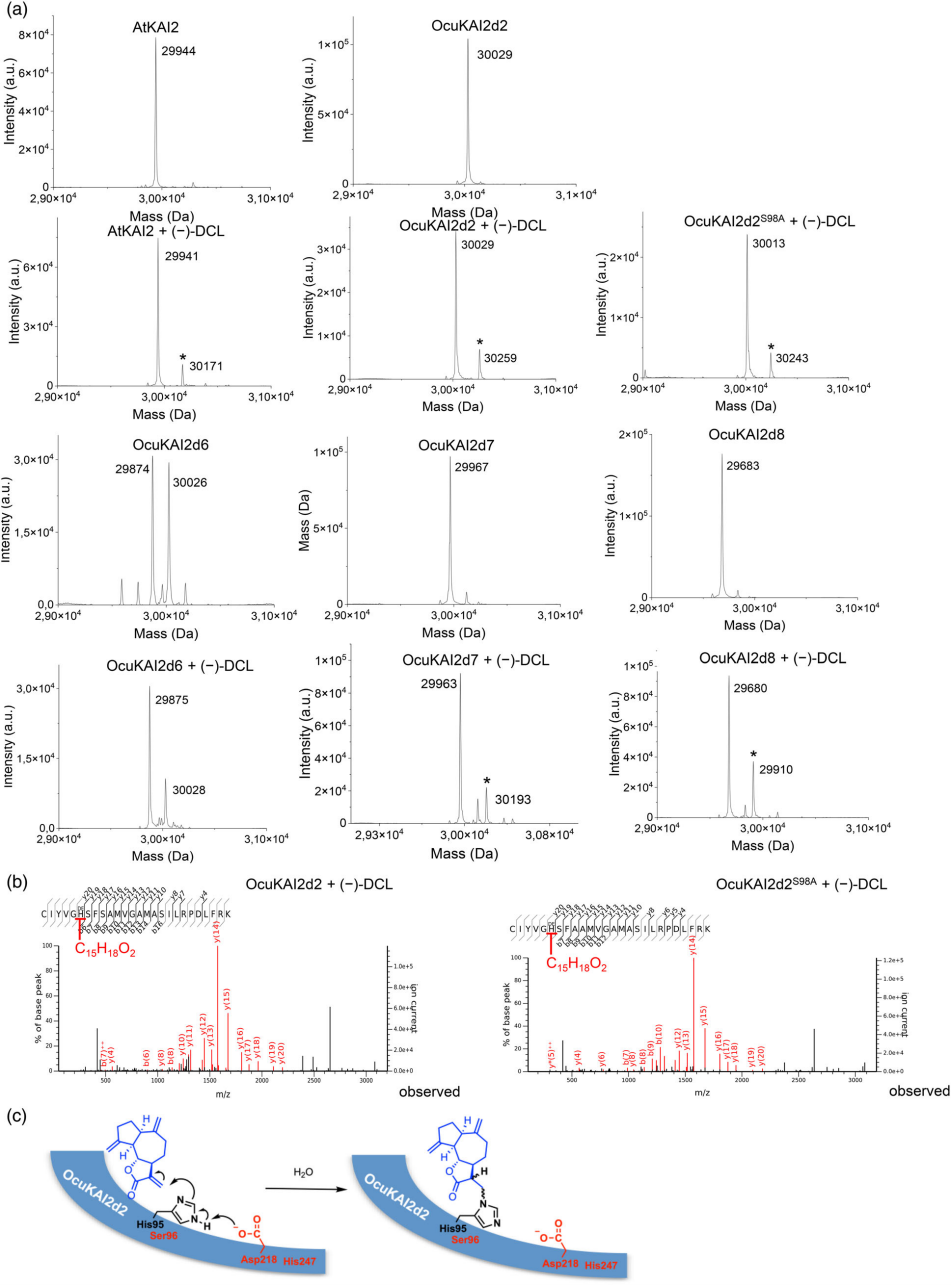
Sesquiterpene lactones form a covalent adduct with the Histidine of the OcuKAI2d2 protein. (a) Deconvoluted electrospray mass spectra of OcuKAI2d proteins (OcuKAI2d2, OcuKAI2d6, OcuKAI2d7, OcuKAI2d8) and AtKAI2 after adding (−)‐DCL ligand are shown. Peaks with an asterisk correspond to protein covalently bound to a (−)‐DCL ligand. A mass increment of 230 Da is measured for all proteins except for OcuKAI2d6. (b) Ligand‐modified amino acids were identified by nano‐LC‐MS/MS analyses after trypsin proteolysis. Labeled peaks correspond to the b and y fragments of the double‐charged precursor ion displayed at the top. The histidine residue modified by (−)‐DCL is underlined. (c) Proposed mechanism for DCL perception. In red, amino acids of the catalytic triad.

Finally, we hypothesized that the interaction of OcuKAI2d proteins with SqTLs may trigger the formation of a covalent adduct. Indeed, a mass shift of 230 Da was detected correlating to the (−)‐DCL covalently bound to OcuKAI2d2, OcuKAI2d7, OcuKAI2d8, or AtKAI2 as previously described (Figure 7a) (Han et al., 2024). After the OcuKAI2d2‐DCL complex digestion, the (−)‐DCL attachment was localized on a peptide corresponding to the amino acids 90–115 of OcuKAI2d2. Surprisingly, tandem mass spectrometry data revealed that the (−)‐DCL attachment was on His95 and not on Ser96 or Ser98 as described in Han et al. (2024) (Figure 7b). Despite evidence for the formation of a covalent adduct between DCL and OcuKAI2d6, OcuKAI2d7, OcuKAI2d8, or AtKAI2 (Figure 7a), it was not possible to localize the covalent adduct on these four proteins due to the formation of non‐specific covalent adducts following the enzymatic digestion required for analysis. These results may explain the different observation reported by Han et al. (2024). These results suggest a DCL perception mechanism in which His95 reacts with DCL to generate an OcuKAI2d2‐DCL adduct via a Michael addition mechanism (Figure 7c). However, our experiments cannot exclude the possibility that the other proteins also operate through the same mechanism.

## DISCUSSION

Unlike some broomrape species, such as *P. ramosa*, *O. cumana* has specialized on sunflower as its single crop host (Parker, 2013). However, the molecular mechanism underlying this fine‐tuned recognition process remains poorly understood. Recent studies suggested that *O. cumana* possesses seven KAI2 paralogs (Han et al., 2024), whose interactions with sunflower‐produced GS may explain the process, although they have not been fully characterized.

### Expansion of the KAI2d protein family in *O. cumana*

In our study, we used whole genome sequences and identified 10 genes of KAI2 family in two *O. cumana* populations (Xu et al., 2022; this study), including nine *KAI2d* paralogs and one *KAI2c*, highlighting a more complex multigene family than previously established (Conn et al., 2015). In the genus *Striga*, it is commonly assumed that the multiplication of KAI2d/HTL receptors is correlated with the number of host species parasitized (Yoshida et al., 2019). However, this assumption is challenged in broomrape species: *O. cumana*, which has only been described on *Helianthus annuu*s, possesses a higher number of GS receptors than the more generalist parasite *P. ramosa*, which possesses only four KAI2d paralogs (de Saint Germain et al., 2021). *O. cernua* and *O. cumana* share a recent common ancestor but are recognized as two distinct species (Benharrat et al., 2002; Pineda‐Martos et al., 2014; Pujadas‐Salvà & Velasco, 2000). Speciation is thought to have occurred when *O. cumana* specialized on sunflower, in connection with the expansion of its cultivation, possibly originating from a wild *O. cernua* population parasitizing *Artemisia* spp. (Parker, 2013). Interestingly, *O. cernua* and *Orobanche coerulescens* – a non‐weedy root parasite that parasitizes species of the genus *Artemisia* – possess only 3 and 5 *KAI2d* genes, respectively (Bürger et al., 2025; Conn et al., 2015; Kim et al., 2024). Thus, the expansion of the number of KAI2d encoding genes in *O. cumana* could have facilitated the transition to a new host. Indeed, while SLs induced seed germination of both *O. cernua* and *O. cumana*, only the latter can perceive sunflower‐derived SqTLs, supporting this hypothesis (Dor et al., 2020; Fernández‐Aparicio et al., 2011; Raupp & Spring, 2013). As a matter of fact, the germination of *O. cumana* is stimulated by these two families of specialized metabolites that exhibit a large structural diversity (Daignan‐Fornier et al., 2024; Frey et al., 2024). *Helianthus annuus* synthesizes different SqTLs and SLs and several of them have shown an activity on *O. cumana* seed germination (Bharti et al., 2015; de Luque et al., 2000; Galindo et al., 2002). However, only four SqTLs − comprising the DCL and the costunolide − and one SL, heliolactone, have been detected in sunflower root exudates (Joel et al., 2011; Raupp & Spring, 2013; Ueno et al., 2014). Thus, it remains possible that other SLs and SqTLs could be exudated under environmental conditions different from those used for their detection. In this context, it can be proposed that an increase in the number of copies of *KAI2d‐*encoding genes enabled *O. cumana* to detect a broader range of SLs and SqTLs specific to sunflowers. Thereby, we can speculate that *KAI2* genes have evolved, through duplication, into numerous copies of the *KAI2d* gene, leading to their neofunctionalization and specialization to the sunflower. Conversely, *KAI2c* remains in a single copy that is conserved in both parasitic and non‐parasitic plants (Conn et al., 2015). The function of this paralog is still not fully understood and has not been characterized in parasites yet.

### Among strigolactones *O. cumana* seeds prefer strigol and non‐canonical‐type for their germination

As suggested by previous studies (Chen et al., 2021; de Saint Germain et al., 2019; Fernández‐Aparicio et al., 2011), we confirmed that *O. cumana* has a strong stereochemical preference for strigol‐type SL over orobanchol‐type SL, for which it shows very low affinity. This contrasts with other parasitic *Orobanchaceae* species, most of them displaying at least moderate affinity for both SL types (de Saint Germain et al., 2019; Fernández‐Aparicio et al., 2011). Additionally, the parasite is much less sensitive to contalactone, a by‐product of GR24 synthesis, as well as to (−)‐GR24 compared with *P. ramosa* and *S. hermonthica* (de Saint Germain et al., 2019). On the other hand, except for *O. minor*, no other parasitic plant is able to perceive the (−)‐DCL (Brun et al., 2019; Takei et al., 2023; Ueno et al., 2014). These observations indicate that *O. cumana* is highly selective for a group among the stereoisomers within SLs, a necessary ability for a parasite with a restricted host range. Indeed, as *O. cumana* specialized in parasitizing sunflower, we can hypothesize that the plant has a selective advantage in not recognizing GS from other potential hosts, thereby preventing parasitism on plants that exude orobanchol‐type SLs, such as legumes (Fernández‐Aparicio et al., 2009). Therefore, it is not surprising that the interaction, the cleavage and the stimulation are more effective with all heliolactone isomers, which display greater flexibility and allow, for example, the (−)‐6‐*epi*‐heliolactone to perform far better than the (−)‐GR24, even though they share similar conformations.

### Do OcuKAI2d proteins play other roles than the perception of GS?

In this study, we demonstrated that four OcuKAI2d proteins exhibit high affinity for natural GS from sunflowers (DCL, heliolactone) as well as for synthetic SLs (GR24) in *in vitro* assays, and thus may be involved in the germination process. The implication of the other paralogs remains speculative. Although Han et al. (2024) argue that OcuKAI2c can perceive DCL, the expression of *OcuKAI2c* and *OcuD14* genes during the life cycle suggests that they are not involved in the germination induction. Among the KAI2d paralogs that were not tested *in vitro*, molecular docking experiments showed that OcuKAI2d1, OcuKAI2d5, and OcuKAI2d9 displayed strong affinities for most ligands. In particular, OcuKAI2d1 is a promising candidate as a more generalist receptor for SLs. Conversely, OcuKAI2d4 exhibited very low affinity for almost all tested ligands. This can be explained by the composition of its binding pocket, which contains more bulky amino acids that cause steric hindrance and reduce the cavity volume (Figure 3b; Figure S7). OcuKAI2d4 is the only KAI2d paralog harboring a phenylalanine (Phe) amino acid in 231 and 256 locations (195 and 220 in AtD14), which determine the volume of the binding pocket and play an important role in GR24/KARs selectivity of the HTL witchweed *Striga hermonthica* proteins (Toh et al., 2015; Wang et al., 2022; Xu et al., 2018). These observations, together with the neutral selection acting on its sequence, suggest that this copy may not play a role in germination induction.

OcuKAI2d2, OcuKAI2d6, OcuKAI2d7, and OcuKAI2d8 do not display any differences in the *in vitro* interactions with the strigol‐type analog (+)‐GR24 and the orobanchol‐type analog (−)‐2′‐*epi*‐GR24, and both are preferentially hydrolyzed. However, the previously described weak activity of orobanchol‐type SLs on the seeds seems to contradict these data. This could explain why cleavage capacity does not always correlate with bioactivity. For example, AtD14 and RMS3 fully cleave (−)‐GR24 and (±)‐GC486 *in vitro* (de Saint Germain et al., 2016, 2021), yet they are weakly and not bioactive for the shoot branching inhibition, respectively (de Saint Germain et al., 2016; Scaffidi et al., 2014). In addition, we cannot rule out the possibility that another KAI2d protein, which is sensitive only to strigol‐type SL, plays a determinant role in the germination induction.

Therefore, some KAI2d proteins might have adapted their recognition process to SqTLs or SLs. However, we cannot exclude that these proteins have functions beyond GS perception. Although the expression of divergent receptors decreases after germination induction, their transcripts are still detected during haustorium development, leading to papillae formation (Figure 2c). Thus, some KAI2d paralogs may play a role in chemiotropism as in *P*. *japonicum* which detects SL from its host to redirect its radicle (Ogawa et al., 2022). In *O. cumana*, the chemiotropic signal would consist of at least a costunolide gradient as suggested by Krupp et al. (2021). White et al. (2024) demonstrated that at least two KAI2d proteins in *P. japonicum* and *S. hermonthica* act in a dominant‐negative manner by attenuating GS‐mediated response. A similar role could be assumed by the highly expressed, but poorly effective *in silico*, OcuKAI2d4, which could capture GS without inducing the germination pathway.

### An original perception mechanism for SqTLs by OcuKAI2d proteins

As previously observed (Han et al., 2024), we confirmed that (−)‐DCL covalently binds AtKAI2 (Figure 7a). In addition, a previous computational study showed a very good affinity of sunflower HaKAI2 receptors for endogenous SqTLs, 8‐*epi*‐xanthatin and tomentosin (Rahimi & Bouwmeester, 2021). Therefore, it seems that the KAI2 receptors are able to perceive SqTLs, suggested to be candidates for the elusive KL. However, further experiments are needed to definitively conclude (Han et al., 2024; Rahimi & Bouwmeester, 2021). By transitioning to parasitism, KAI2d supposedly arose from KAI2 by specializing in SL perception. Thus, at least the KAI2d receptors of *O. cumana* and *O. minor* may have conserved the capacity to accommodate SqTLs and gained the ability to redirect it toward the induction of germination (Brun et al., 2019; Takei et al., 2023; Ueno et al., 2014; this study), but through a different mechanism that does not involve cleavage or destabilization.

Here, we demonstrated that among the nine OcuKAI2d proteins, at least four function as SL receptors. Binding assays and mass spectrometry analyses showed that OcuKAI2d2, OcuKAI2d6, OcuKAI2d7, and OcuKAI2d8 covalently interact with a SqTL such as DCL. Our results indicate that OcuKAI2d receptors are involved in the perception of non‐SL‐type GS. For OcuKAI2d2, we identified that DCL is covalently bound to a histidine in the binding pocket adjacent to the serine of the catalytic triad, rather than the serine residues described previously (Han et al., 2024). We thus elucidate the complete molecular mechanism underlying the SqTL‐dependent perception in *O. cumana* and propose a mechanism for DCL interaction with OcuKAI2d proteins. DCL reacts with the imidazole nitrogen of His95 in OcuKAI2d2 via a Michael addition mechanism to form a covalent adduct with the non‐cleaved molecule (Figure 7c). Due to the moderately nucleophilic nature of the histidine imidazole side chain (Weerapana et al., 2008), modified histidine residues can undergo specific phosphorylation (Zhong et al., 2023) or methylation (Petrovic et al., 2018) and serve as targets for small molecules (Lowther et al., 1998). Notably, histidine‐specific aza‐Michael addition reactions with α,β‐unsaturated compounds have already been reported in various proteins (Tsou et al., 2024; Uchida & Stadtman, 1992).

### Potential innovative *O. cumana* control methods

In addition to the genetic strategy involving the breeding of resistant cultivars, our results may offer insights in the first steps of developing innovative *O. cumana* management methods. The parasite's affinity for (+)‐costunolide and (−)‐DCL raises the possibility of using these compounds as suicide GS, as suggested by Han et al. (2024), who observed a strong decrease in *O. cumana* infestation when previously treated with (−)‐DCL. To the best of our knowledge, no study was published on the stability of sunflower‐derived GS in soil; however, parthenin, a pseudoguaianolide resembling DCL, has an average half‐life of 59 h in soil (Belz et al., 2009; Moriyama et al., 2022; Wallach et al., 2024). An alternative chemical approach has been explored, though solely for targeting *Striga*, through the development of various selective inhibitors of the SL receptor ShHTL7 (Arellano‐Saab et al., 2022; Du et al., 2024; Holbrook‐Smith et al., 2016; Shahul Hameed et al., 2018; Zarban et al., 2022). This strategy also deserves to be developed to combat *O. cumana* by targeting OcuKAI2d proteins. Another strategy could involve the use of a crop bait, such as the non‐host black oat (*Avena strigosa*), which exudes the particularly efficient *O. cumana* GS (+)‐6‐*epi*‐heliolactone (Moriyama et al., 2022).

## METHODS

### Plant material and growth conditions

The population of *O. cumana* used in this study is OcIN23 (US2B code Ocum52.2), harvested in 2020 in Córdoba (Spain) and kindly provided by Begoña Pérez Vich. Dried seeds were stored at 25°C in darkness before use. For expression analysis following GS treatments, another population displaying the same phenotype and possessing identical KAI2 sequences was used (Ocum49, harvested in Longeville‐sur‐mer, France, in 2017). Seeds of *O. cumana* were surface sterilized and conditioned for 7 days in darkness at 21°C, as detailed (Pouvreau et al., 2021). The cultivated sunflower used for mRNA sequencing after infection with *O. cumana* was the 2603 accession, which is susceptible to *O. cumana* race F, and was grown in minirhizotron systems as previously described (Letousey et al., 2007).

### Chemicals

See Supporting Information.

### *O. cumana* germination bioassays

Activity of the GS on the seeds of *O. cumana* was assessed as detailed by Pouvreau et al. (2021). Chemicals (Supporting Information; Figure S1) were suspended at 10^−2^ M in a solvent (DMSO or acetonitrile). For each test, a dilution at 10^−4^ M in water was performed, followed by a cascade dilution ranging from 10^−5^ to 10^−12^ M for potential stimulants and from 10^−4^ to 10^−15^ M for the profluorescent ligands in water/solvent (v/v; 99:1). Conditioned seeds of *O. cumana* were treated with diluted chemicals from 10^−6^ to 10^−13^ M for potential stimulants and from 10^−5^ to 10^−16^ M for profluorescent ligands. GS activity was then expressed as a relative ratio to the germination with (±)‐GR24 1 μM treated seeds within the same plate. For each test, the four‐parameter logistic model implemented in the R package “drc” was used as described in Pouvreau et al. (2021) to generate dose–response curves relative to the positive control, EC_50_, and maxima of GS activity. When no significant GS activity was induced or below a 0.5 threshold, the EC_50_ were not determined. Each experiment was conducted at least three times.

### Identification of *OcuKAI2* and *OcuD14* sequences in the *O. cumana* genome

The identification of *OcuKAI2* and *D14* sequences within the genome was achieved by BlastN and tBlastX on the annotated genome using specific *KAI2/D14* sequences from different *Orobanchaceae* species as templates (Conn et al., 2015; de Saint Germain et al., 2021; Toh et al., 2015). The same procedure was performed on the previously sequenced genome by Xu et al. (2022). The sequences have been deposited on NCBI under the following accession codes: PX844445 (*OcuKAI2c*); PX844446 (*OcuD14*); PX844447 (*OcuKAI2d1*); PX844448 (*OcuKAI2d2*); PX844449 (*OcuKAI2d3*); PX844450 (*OcuKAI2d4*); PX844451 (*OcuKAI2d5*); PX844452 (*OcuKAI2d6*); PX844453 (*OcuKAI2d7*); PX844454 (*OcuKAI2d8*); PX844455 (*OcuKAI2d9*). Gene structures and distribution on chromosomes were respectively visualized with the online tools GSDS 2.0 and MG2C.

### Phylogenetic analysis

Along with the 11 sequences described in this study, 46 KAI2 and D14 from *P. ramosa*, *P*. *aegyptiaca*, *S. hermonthica*, *O. minor*, *O. cernua*, *P. japonicum*, and *A. thaliana* (Conn et al., 2015; Toh et al., 2015; Waters et al., 2012) were retrieved to perform phylogenetic analysis. As an outgroup for the α/β‐hydrolase receptors, we used RsbQ from *Bacillus subtilis* as its sequence contains the conserved catalytic triad (de Saint Germain et al., 2021; Waters et al., 2012). Amino acid sequences were aligned using the ClustalW multiple alignment algorithm. This alignment was used to infer evolutionary history by using the Maximum Likelihood method and JTT matrix‐based model (Jones et al., 1992) with 500 bootstrap replicates. The tree with the highest log likelihood (−11 560.16) was chosen. The percentage of trees in which the associated taxa clustered together is shown next to the branches. Initial tree(s) for the heuristic search were obtained automatically by applying Neighbor‐Join and BioNJ algorithms to a matrix of pairwise distances estimated using the JTT model and then selecting the topology with superior log likelihood value. The tree is drawn to scale, with branch lengths measured in the number of substitutions per site. There was a total of 310 positions in the final dataset. The number of synonymous substitutions and non‐synonymous sites between sequences (Ka/Ks) was determined using a Nei‐Gojobori model. All positions containing gaps and missing data were eliminated (complete deletion option) in the estimation of synonymous substitutions. There was a total of 251 positions in the final dataset. Evolutionary analyses and Ka/Ks ratio were conducted in MEGA11 (Tamura et al., 2021), and alignment visualization was made with Jalview (Waterhouse et al., 2009).

### RNAseq experiments

See Supporting Information. Development‐wide RNA sequencing data are presented in Table S4.

### PCR, cloning, and sequencing

See Data S1.

### Computational modeling of the protein structure and molecules

The *O. cumana* proteins were modeled by homology using the software MODELER (v10.5) (Šali & Blundell, 1993). The models of the nine divergent *O. cumana* receptors were generated using chain A of the OmKAI2d4 (PDB 7UOC) (Takei et al., 2023) as a template. Concerning the structures of OcuKAI2c and OcuD14, they were obtained using the template ShHTL1 (PDB 5Z7W) and ShD14 (PDB 5Z7Z) (Xu et al., 2018), respectively. In total, 50 models per protein were generated and only the best model was conserved for each, using the Discrete Optimized Protein Energy score (DOPE score) given by MODELER. For each model of KAI2/D14, an all‐atom refinement of structures has been applied using the relax main protocol of Rosetta (v2024.09) (Tyka et al., 2011) force‐field creating 10 full‐atom relaxed structures. The protein structures figures were generated with PyMOL (PyMOL Molecular Graphics System, Version 2.5.7 Schrödinger, LLC) using the best model given by Rosetta for each. Pocket sizes were calculated using the CASTpFold server with a probe radius of 1.4 Å (Ye et al., 2024). The reported pocket sizes were the Connolly solvent excluded surface volumes of the catalytic pocket. Residues in contact with the solvent were retrieved and aligned for comparison.

### *In silico* molecular docking assays

Molecules were modeled using Maestro (Schrödinger 2024‐4). All molecules and all 10 relaxed structures for each protein were prepared via MGLTools (v.1.5.6) (https://ccsb.scripps.edu/mgltools/) (Sanner, 1999). AutoDock Vina (v1.2.5) (Eberhardt et al., 2021) was used to perform the docking experiments. The searching box was centered on the binding hydrophobic pocket with a box size of 18.75 × 18.75 × 18.75 Å. Output results were analyzed to keep only the ones that showed the lowest Gibbs free energy values within a 4 Å distance between the butenolide moiety for SL and the oxygen atom from the serine of the catalytic triad. The heatmap was generated using Python libraries Matplotlib (v.3.8.2) (Hunter, 2007) and Seaborn (v.0.13.2) (Waskom, 2021), and affinity values were marked as ND (not determined) otherwise.

### Protein expression and purifications

See Supporting Information.

### Site‐directed mutagenesis

See Supporting Information.

### Enzymatic hydrolysis of GR24 isomers by purified proteins

See Supporting Information.

### Enzymatic assays with profluorescent probes

See Supporting Information.

### nanoDSF

See Supporting Information.

### Direct electrospray ionization – mass spectrometry of OcuKAI2 proteins

See Supporting Information.

### NanoLC‐MSMS peptide analysis of OcuKAI2 proteins

See Supporting Information.

## AUTHOR CONTRIBUTIONS

AdSG, SM, F‐DB, J‐BP and PD designed the research. AK synthesized and purified heliolactone isomers. AK and F‐DB synthesized the chemicals. AdSG and LL produced and purified the proteins. AdSG and LL characterized the proteins and performed the kinetic experiments. JA, PL, SD, AdSG, and J‐BP performed the plant experiments. DC performed the mass spectrometry experiments. AdSG and F‐DB performed the high‐performance liquid chromatography analyses. SP performed the computational modeling and molecular docking experiments. AdSG, JA, LL, SP, F‐DB, J‐BP, ST and PD analyzed the data. AdSG, JA, F‐DB and PD wrote the paper. All authors critically revised the manuscript.

## CONFLICT OF INTEREST

The authors declare no conflict of interest.

## Supporting information


**Figure S1.** Ligand chemical structures. (a) Strigolactones (GR24: artificial canonical SL; heliolactone: non‐canonical SL). (b) Profluorescent probes: coumarin‐based probes in blue (GC series); fluorescein‐based probes in green (YLG series). (c) Sesquiterpene lactones, isothiocyanate, and DiFMU.
**Figure S2.** Phenotyping seed germination of *O. cumana* by stimulation with ligands and profluorescent probes. Molecules were applied from 10^−13^ to 10^−6^ M for tested ligands and from 10^−15^ to 10^−5^ M for profluorescent probes and GS activity is relative to 1 μM (±)‐GR24. The dose–response curves were modeled as described by Pouvreau et al. (2021) and all raw data points are shown. The modeling of curves was not conducted when the activity at the maximum concentration did not demonstrate statistical differences from the activity at the minimum concentration.
**Figure S3.** Analysis of KAI2 paralog sequences between genomes. Phylogenetic analysis of KAI2 and D14 nucleotide sequences (At, *A. thaliana*; Oce, *O. cernua*; Ocu, *O. cumana*; Om, *O. minor*; Pa, *P. aegyptiaca*; Pj, *Phteirospermum japonicum*; Pr, *P. ramosa*; Sh, *Striga hermonthica*). The evolutionary history of nucleotide sequences was inferred using the neighbor joining method. The optimal tree is shown. The percentage of replicate trees in which the associated taxa clustered together in the bootstrap test (1000 replicates) are shown above the branches. The evolutionary distances were computed using the Maximum Composite Likelihood method and are in the units of the number of base substitutions per site. *O. cumana* sequences from this study and from Xu et al. (2022) are represented in red and blue respectively. Evolutionary analyses were conducted in MEGA11.
**Figure S4.** Structure representation of *OcuKAI2* and *OcuD14* genes. Intron sequences were retrieved from the OcIN23 genome and sequences were aligned on their first exon. The graph was made with the online tool GSDS 2.0, using a phylogenetic tree constructed on MEGA11 with the Neighbor‐Joining method (1000 bootstrap replicates).
**Figure S5.** Sequence alignment of KAI2 protein from *Orobanchaceae* species with D14 and KAI2 proteins from *Arabidopsis thaliana* and the bacterial RsbQ. The classical catalytic triad is marked with red stars. The second catalytic triad observed by Han et al. (2024) for SqTL perception is marked with blue stars. The histidine responsible for the binding of DCL in this study is marked with a black star.
**Figure S6.** Correlation between nucleotide sequence identity and distance between *KAI2* and *D14* genes on the chromosome 1. Positions of START codons were retrieved from the OcIN23 genome and used for distance calculation. A clustalW alignment was used to calculate identity percentage of nucleotide sequences. The correlation line and coefficient were inferred by linear regression with SE (gray area) and Pearson's correlation method, respectively.
**Figure S7.** Amino acid sequence alignment of OcuKAI2 binding pocket residues in contact with the solvent. Amino acids are colored according to the Zappo coloring system implemented in Jalview (Waterhouse et al., 2009). Dashes indicate that the amino acids at the indicated positions are not a part of the binding pocket. Positions are relative to the sequence of AtD14.
**Figure S8.** Expression of OcuKAI2d proteins in *E. coli*. (a, b) SDS‐PAGE electrophoresis gel of purified OcuKAI2d2 (30 030.37 Da), OcuKAI2d6 (29 876.14 Da), OcuKAI2d7 (29 967.10 Da) and OcuKAI2d8 (29 683.88 Da) proteins and their mutant in the catalytic triad OcuKAI2d6^S96A^ (29 860.14 Da), OcuKAI2d7^S95A^ (29 951.10 Da) and OcuKAI2d8^S95A^ (29 667.88 Da). (c, d) Deconvoluted electrospray mass spectra of OcuKAI2d2 protein purified without (c) and with protease cocktail inhibitor (d) showing a shift of 154 Da (asterisk) in comparison to the native protein (red triangle) corresponding to a glycerophosphate covalent adduct.
**Figure S9.** The His‐MBP tag masks protein destabilization by (±)‐GR24 and the enzymatic activity of the OcuKAI2d6 protein. (a–d) Thermostability of HisMBP‐OcuKAI2d6 (a, c) and OcuKAI2d6 (b, d), at 10 μM in the absence of ligands (black line) or in the presence of (±)‐GR24 (dotted black line) at 200 μM analyzed by nanoDSF. The panels (a, b) show the changes in fluorescence (ratio *F*_350nm_/*F*_330nm_) with temperature and (c, d) the first derivatives for the *F*_350nm_/*F*_330nm_ curve against the temperature gradient from which the apparent melting temperature (*T*_m_) was determined for each sample. The experiment was carried out twice. (e) Enzymatic kinetics for OcuKAI2d6, OcuKAI2d6^S96A^, HisMBP‐OcuKAI2d6, AtD14, and AtKAI2 proteins at 333 nM incubated with 4 μM of (±)‐GC240. Progress curves during hydrolysis of the probes, monitored (*λ*_em_ 460 nm) at 25°C. The traces represent one of the three replicates and the experiments were repeated at least twice.
**Figure S10.** Thermostability (nanoDSF) of OcuKAI2d2 (a), OcuKAI2d6 (b), OcuKAI2d7 (c) and OcuKAI2d8 (d) at 10 μM with temperature in the absence of ligands (black line) or in the presence of various ligands at 200 μM analyzed by monitoring the changes in fluorescence (ratio *F*_350nm_/*F*_330nm_). The first derivatives for the *F*_350nm_/*F*_330nm_ curve against the temperature gradient from which the apparent melting temperature (*T*_m_) was determined for each sample. The traces represent one of the three replicates and the experiments were repeated at least twice.
**Figure S11.** Thermostability of OcuKAI2d2 (a), OcuKAI2d6 (b), OcuKAI2d7 (c) and OcuKAI2d8 (d) at 10 μM in the absence of ligands (black line) or in the presence of (±)‐GR24 (dotted black line), and the SL profluorescent probes: (±)‐GC486 (green line), (±)‐GC240 (red line) or (±)‐GC242 (purple line) at 200 μM analyzed by nanoDSF. The upper panels show the changes in fluorescence (ratio *F*_350nm_/*F*_330nm_) with temperature and the lower panels the first derivatives for the *F*_350nm_/*F*_330nm_ curve against the temperature gradient from which the apparent melting temperature (*T*_m_) was determined for each sample. The traces represent one of the three replicates and the experiments were repeated at least twice.
**Figure S12.** Mass spectrometry characterization of covalent protein–ligand complexes. Deconvoluted electrospray mass spectra of OcuKAI2d proteins (OcuKAI2d2, OcuKAI2d6, OcuKAI2d7, OcuKAI2d8) mutated versions, and AtKAI2 after adding (±)‐GR24 and (±)‐heliolactone (ligands) are shown. Peaks with an asterisk correspond to protein covalently bound to a D‐ring. A mass increment of 96 Da is measured for all proteins with ligands except for mutated proteins and AtKAI2 in the presence of (±)‐heliolactone.
**Figure S13.** Ligand‐modified amino acids were identified by nano‐LC–MS/MS analyses after trypsin proteolysis. Labeled peaks correspond to the b and y fragments of the double‐charged precursor ion displayed at the top. The histidine residue modified by (±)‐GR24 or (±)‐heliolactone is indicated as GR and HE, respectively, and corresponds to an addition of C_5_H_5_O_2_.
**Figure S14.** Loss of signal of (−)‐DCL after incubation with various proteins. (+)‐GR24 and (−)‐DCL at 10 μM were incubated with OcuKAI2d6, OcuKAI2d7, OcuKAI2d8, OcuKAI2d6^S96A^, OcuKAI2d7^S96A^, OcuKAI2d8^S96A^ at 5 μM for 150 min at 25°C. UPLC‐UV (260 nm) analysis was used to detect the remaining amount of (+)‐GR24 and (−)‐DCL. Bars represent the mean value of the loss of molecule calculated from the remaining (+)‐GR24 and (−)‐DCL, taking into account the effect of the buffer alone (without protein sample), quantified with (±)‐1‐indanol as internal standard. Error bars represent the SD of three replicates (means ± SD, *n* = 3).
**Table S1.** *O. cumana* seed germination phenotyping. EC_50_ and maximum of GS activity are indicated ±SE.
**Table S2.** Descriptions of *O. cumana* developmental stages used for RNAseq experiment.
**Table S3.** Primer table.

**Table S4.** Development‐wide RNA sequencing data of *O. cumana*. The table contains biological metadata per triplicated samples (sheet “metadata”), read count tables for all major life stages (sheet “lifecycle”) or seed responses to (±)‐GR24 and (−)‐DCL (sheet “germination‐stimulants”), gene functional annotation (sheet “functional_annotation”), and gene identifiers corresponding to candidate genes investigated in this study (sheet “candidate_gene_ids”).

**Data S1.** Supplemental information regarding the synthesis of chemical compounds.

## Data Availability

All data supporting the findings of this study, except RNA sequencing data, are included in the article and its supplementary data, and further inquiries are available from the corresponding author. All herein generated RNA sequencing data have been submitted to the Short Read Archive in NCBI, under BioProject PRJNA1414087.
