# Supplementary material for: Strigolactones and sesquiterpene lactones induce Orobanche cumana germination via KAI2d receptors through distinct processes
Source: Plant J. 2026 Apr 16;126(1):e70852. doi: 10.1111/tpj.70852 (PMC13086240; doi:10.1111/tpj.70852)
Supplement: Supplementary file 1 — Figure S1. Ligand chemical structures. (a) Strigolactones (GR24: artificial canonical SL; heliolactone: non‐canonical SL). (b) Profluorescent probes: coumarin‐based probes in blue (GC series); fluorescein‐based probes in green (YLG series). (c) Sesquiterpene lactones, isothiocyanate, and DiFMU. Figure S2. Phenotyping seed germination of O. cumana by stimulation with ligands and profluorescent probes. Molecules were applied from 10−13 to 10−6 M for tested ligands and from 10−15 to 10−5 M for profluorescent probes and GS activity is relative to 1 μM (±)‐GR24. The dose–response curves were modeled as described by Pouvreau et al. (2021) and all raw data points are shown. The modeling of curves was not conducted when the activity at the maximum concentration did not demonstrate statistical differences from the activity at the minimum concentration. Figure S3. Analysis of KAI2 paralog sequences between genomes. Phylogenetic analysis of KAI2 and D14 nucleotide sequences (At, A. thaliana; Oce, O. cernua; Ocu, O. cumana; Om, O. minor; Pa, P. aegyptiaca; Pj, Phteirospermum japonicum; Pr, P. ramosa; Sh, Striga hermonthica). The evolutionary history of nucleotide sequences was inferred using the neighbor joining method. The optimal tree is shown. The percentage of replicate trees in which the associated taxa clustered together in the bootstrap test (1000 replicates) are shown above the branches. The evolutionary distances were computed using the Maximum Composite Likelihood method and are in the units of the number of base substitutions per site. O. cumana sequences from this study and from Xu et al. (2022) are represented in red and blue respectively. Evolutionary analyses were conducted in MEGA11. Figure S4. Structure representation of OcuKAI2 and OcuD14 genes. Intron sequences were retrieved from the OcIN23 genome and sequences were aligned on their first exon. The graph was made with the online tool GSDS 2.0, using a phylogenetic tree constructed on MEGA11 with the Neighbor [file TPJ-126-0-s001.pptx]

## Slide 1
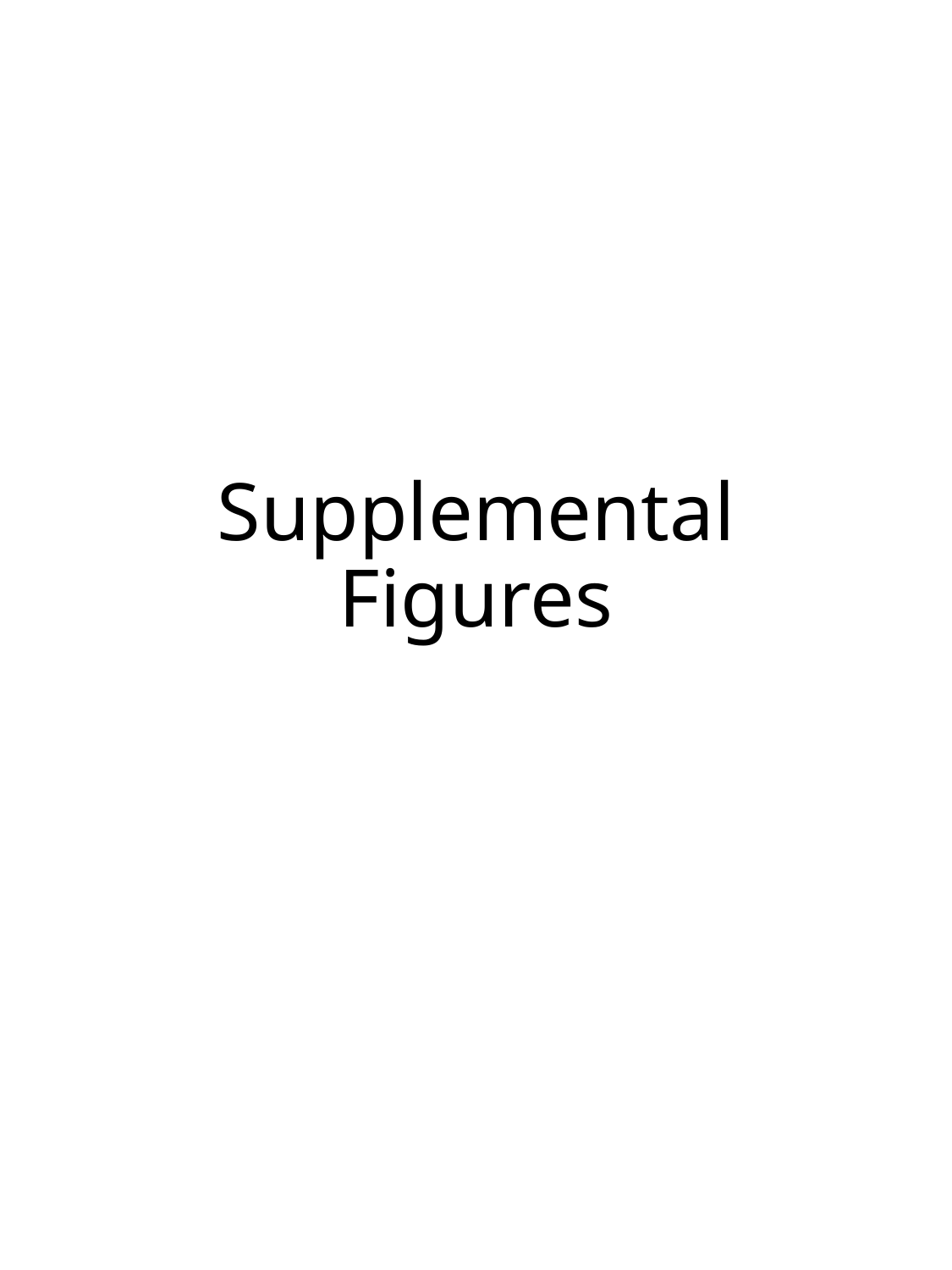

# Supplemental Figures

## Slide 2
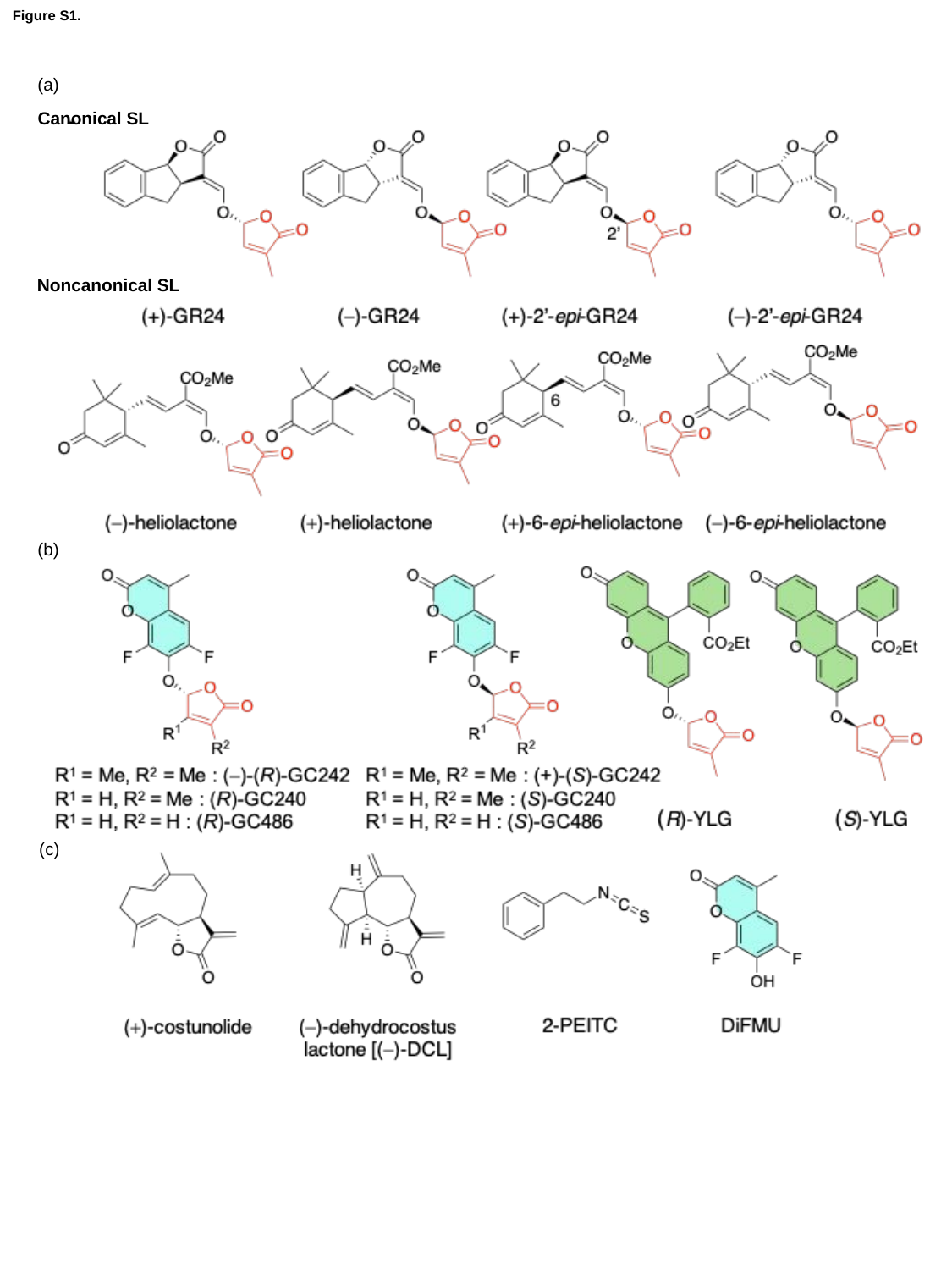

Figure S1.
(a)
Canonical SL
Noncanonical SL
(b)
(c)

## Slide 3
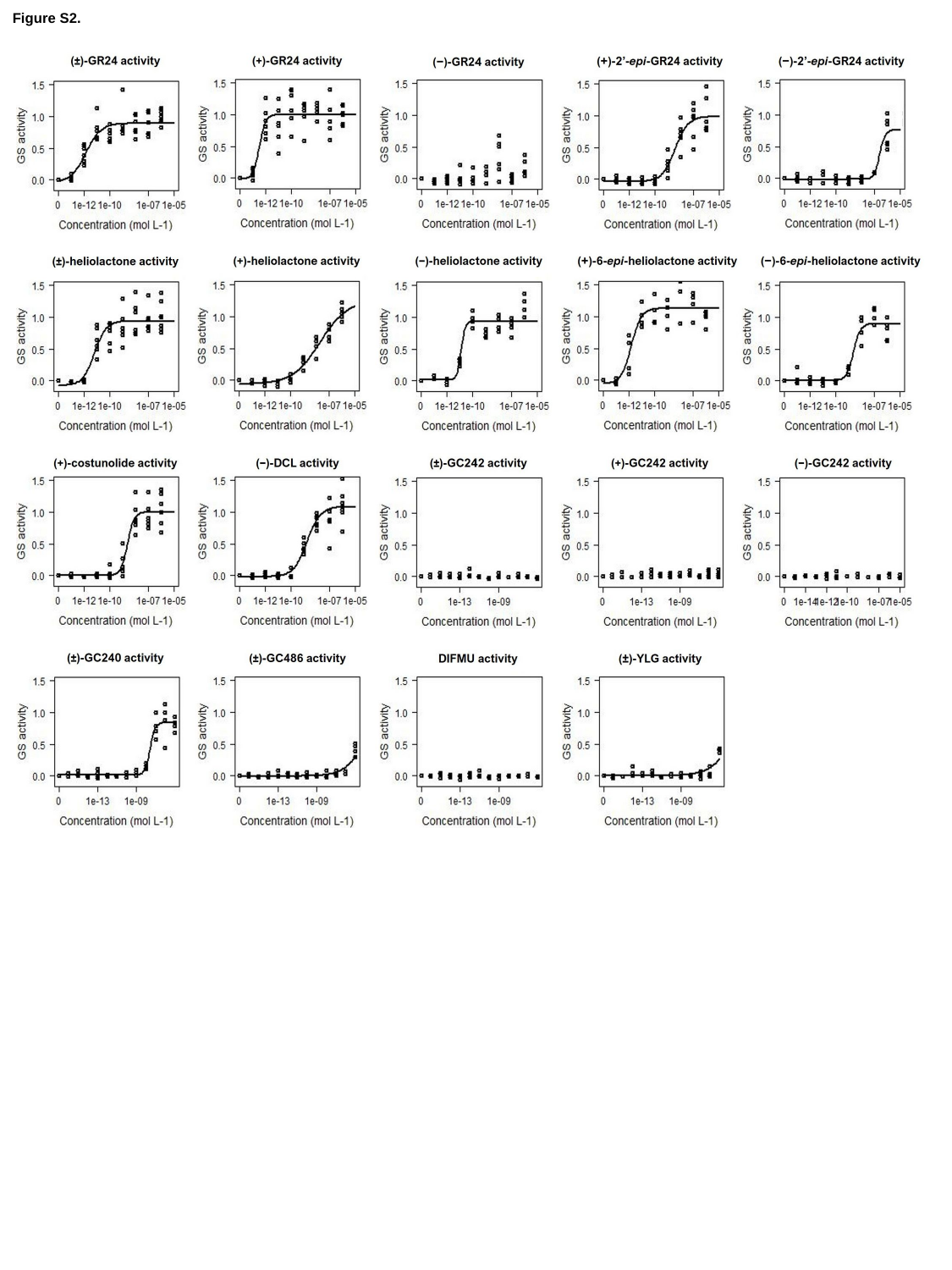

Figure S2.

## Slide 4
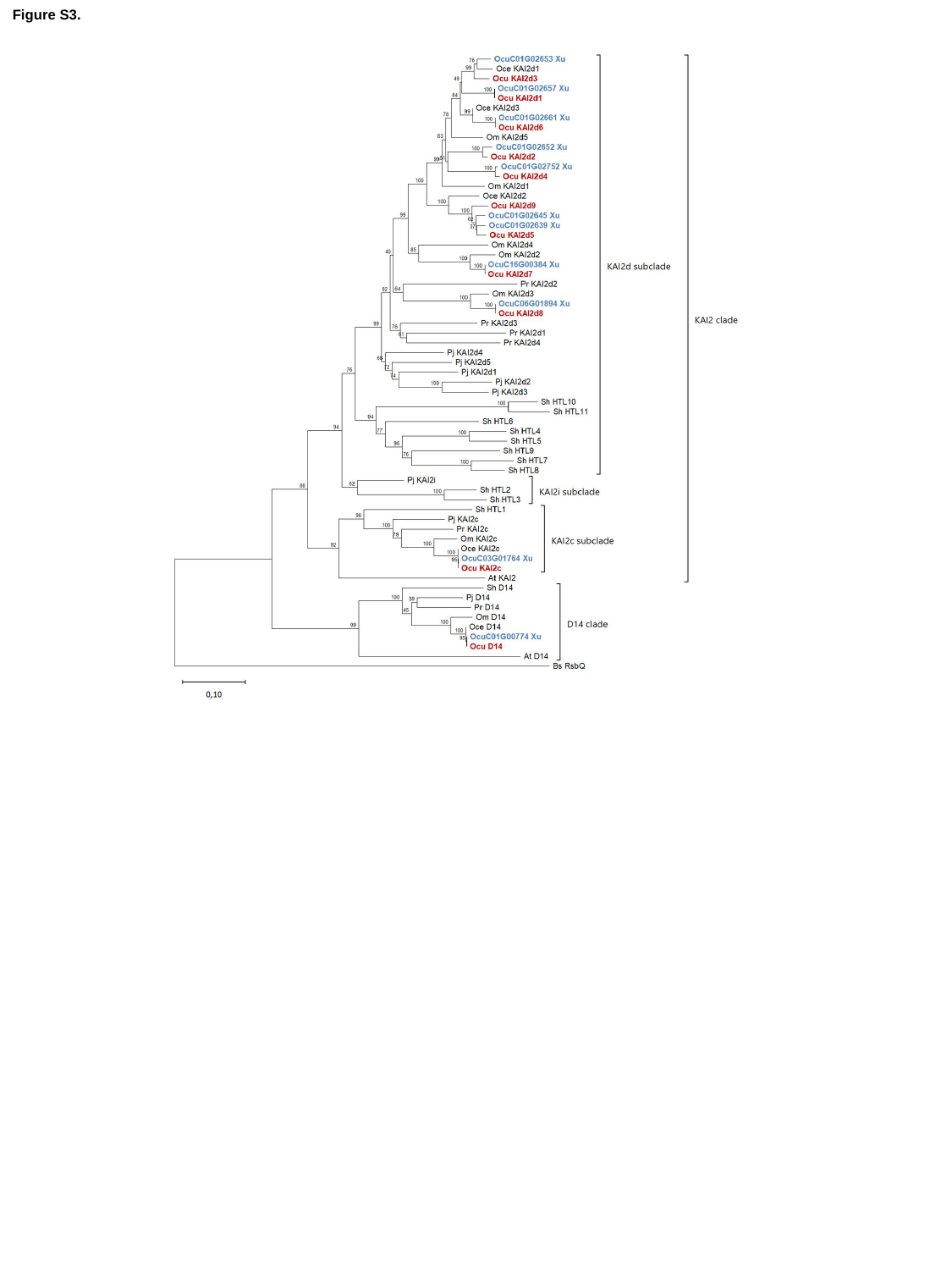

Figure S3.

## Slide 5
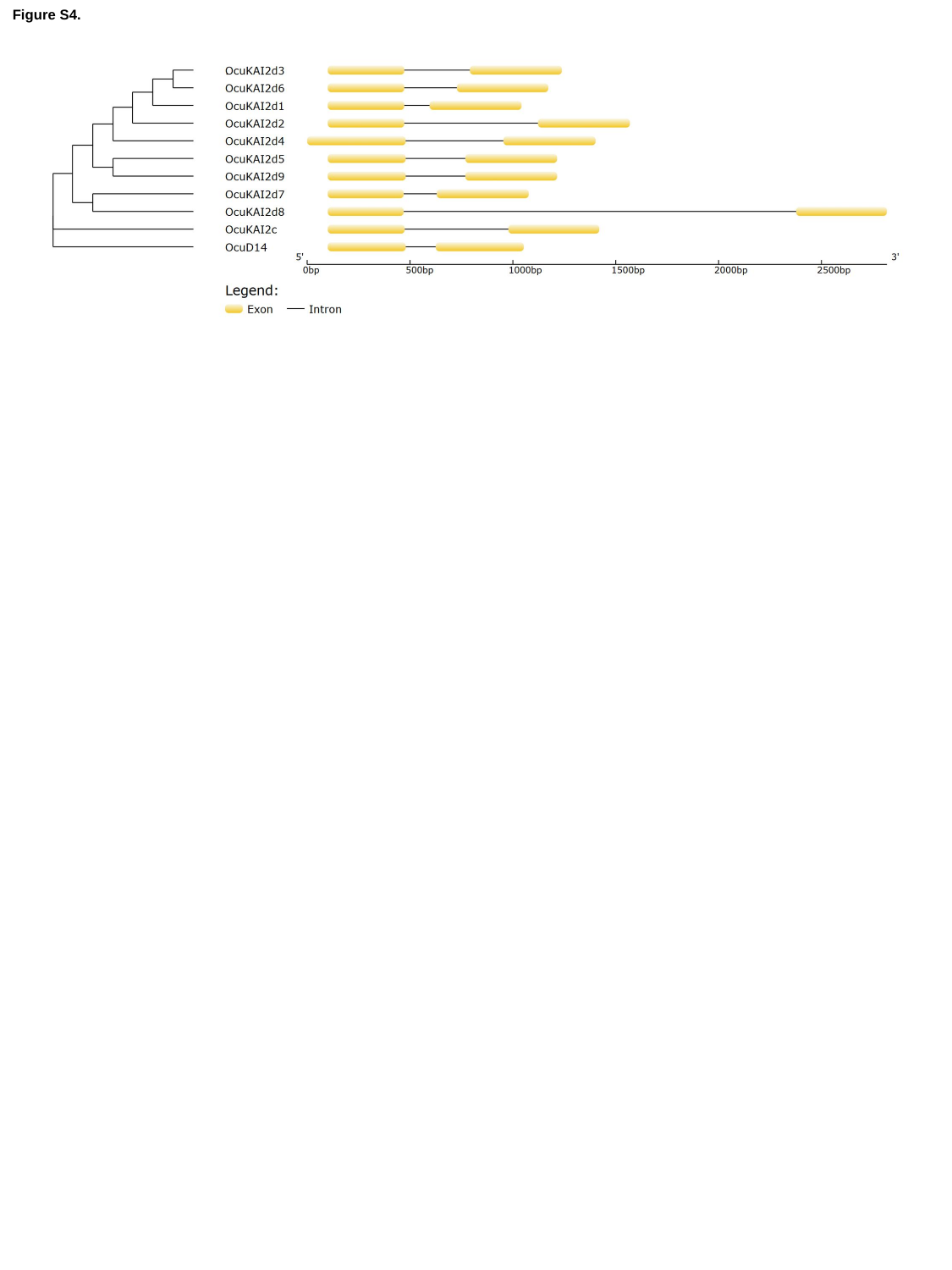

Figure S4.

## Slide 6
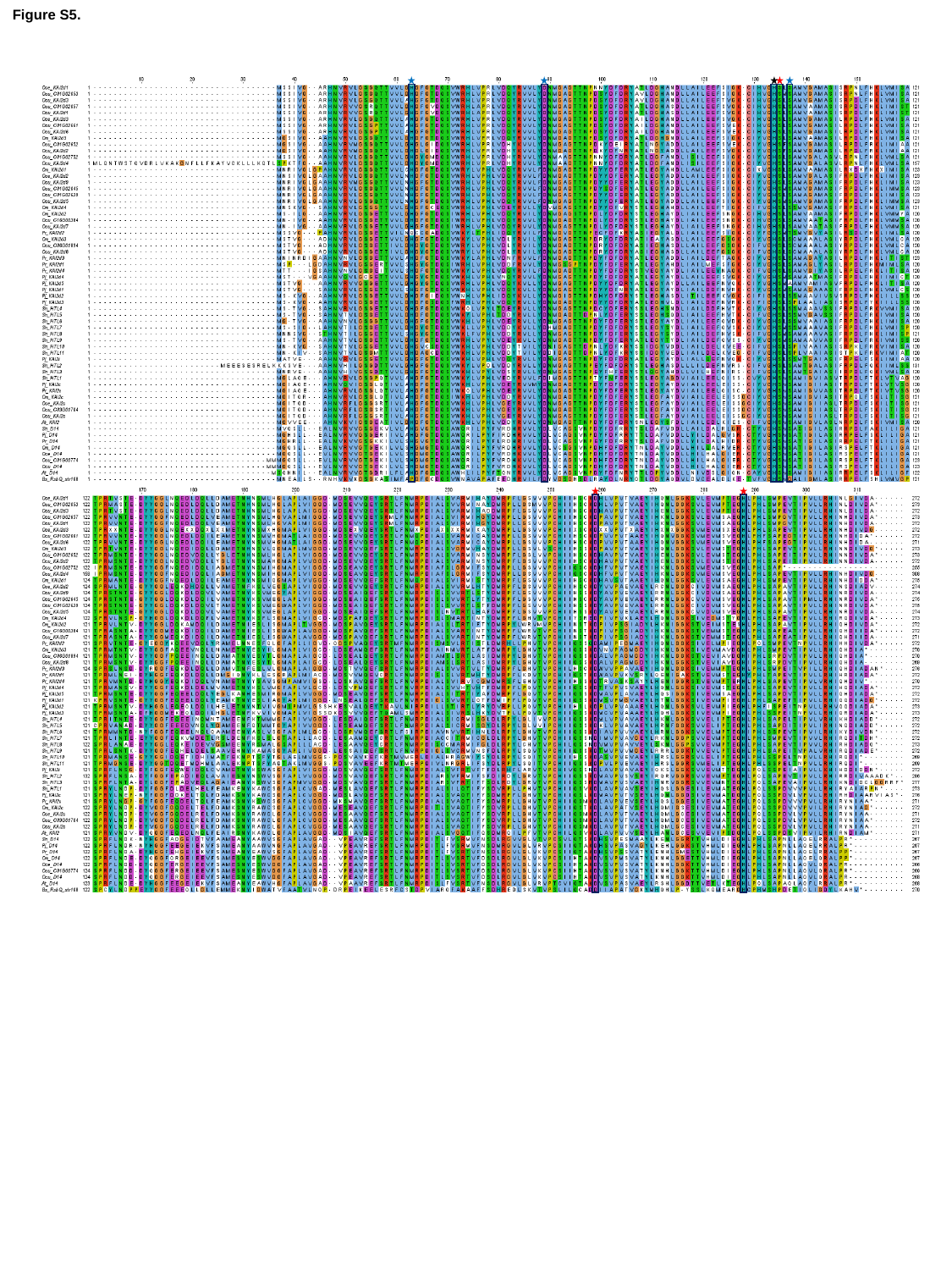

Figure S5.

## Slide 7
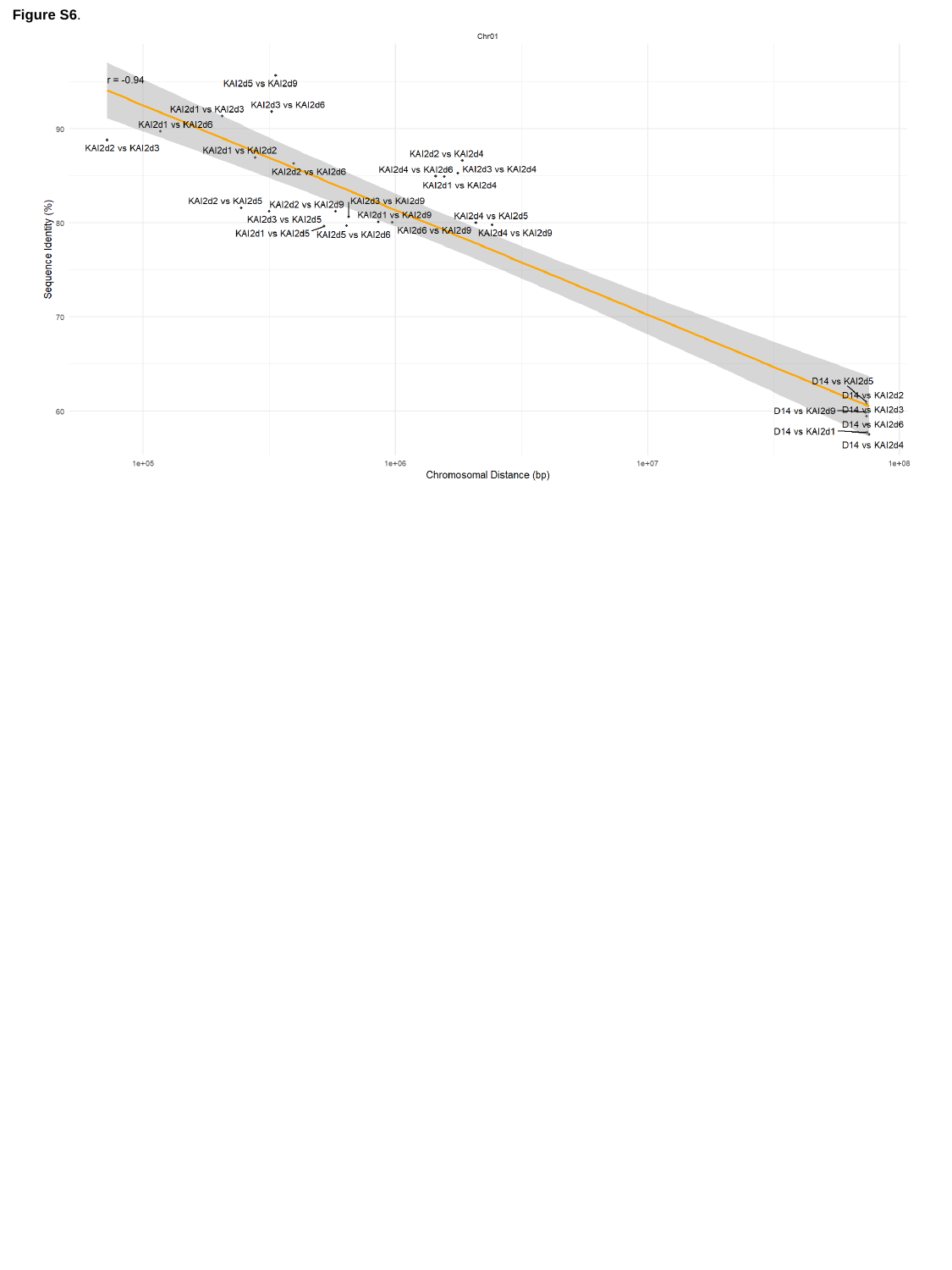

Figure S6.

## Slide 8
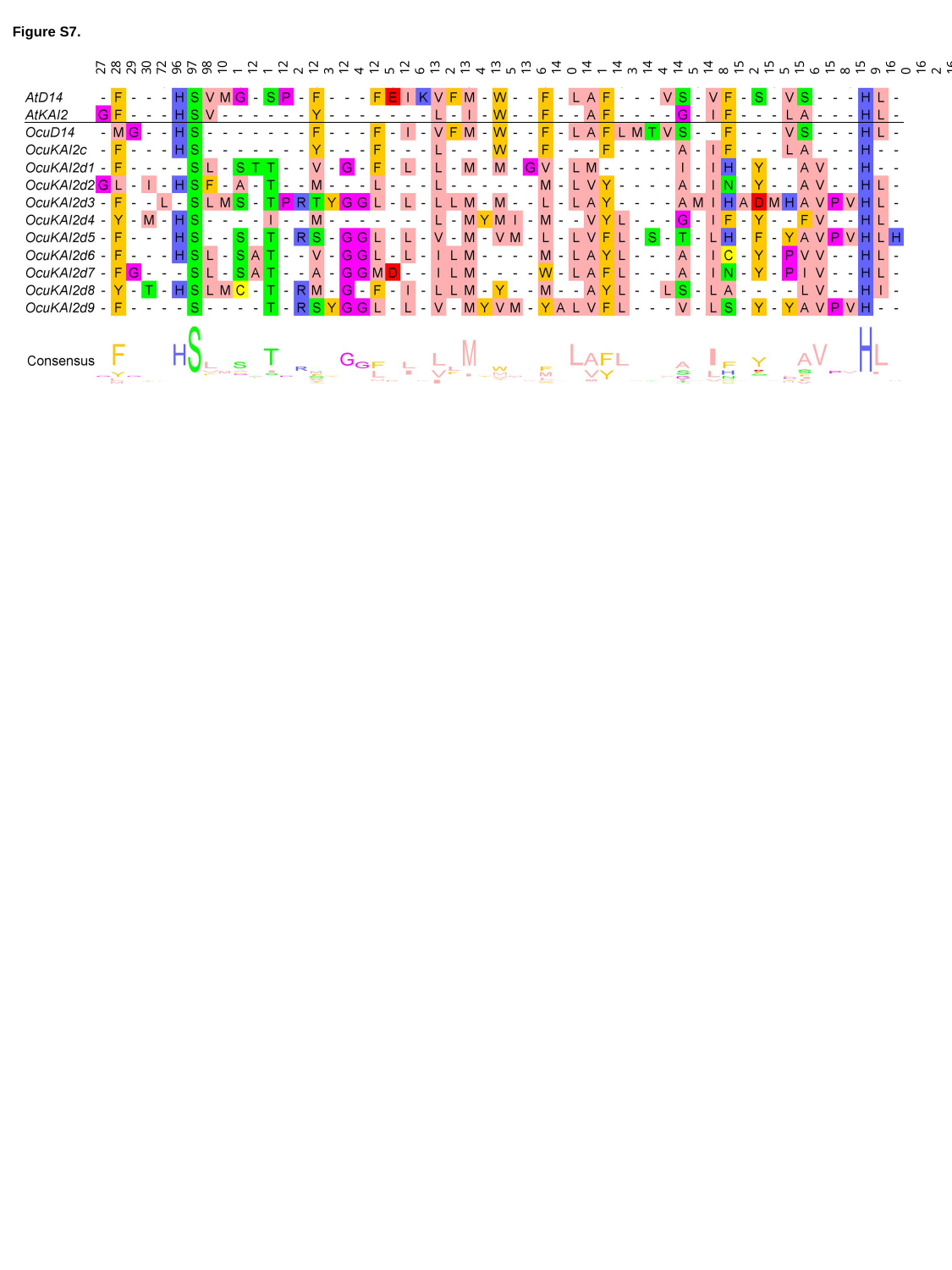

Figure S7.
27 28 29 30 72 96 97 98 101 121 122 123 124 125 126 132 134 135 136 140 141 143 144 145 148 152 155 156 158 159 160 162 163 175 179 182 187 190 191 193 194 195 196 197 198 219 220 221 222 223 247 248 249

## Slide 9
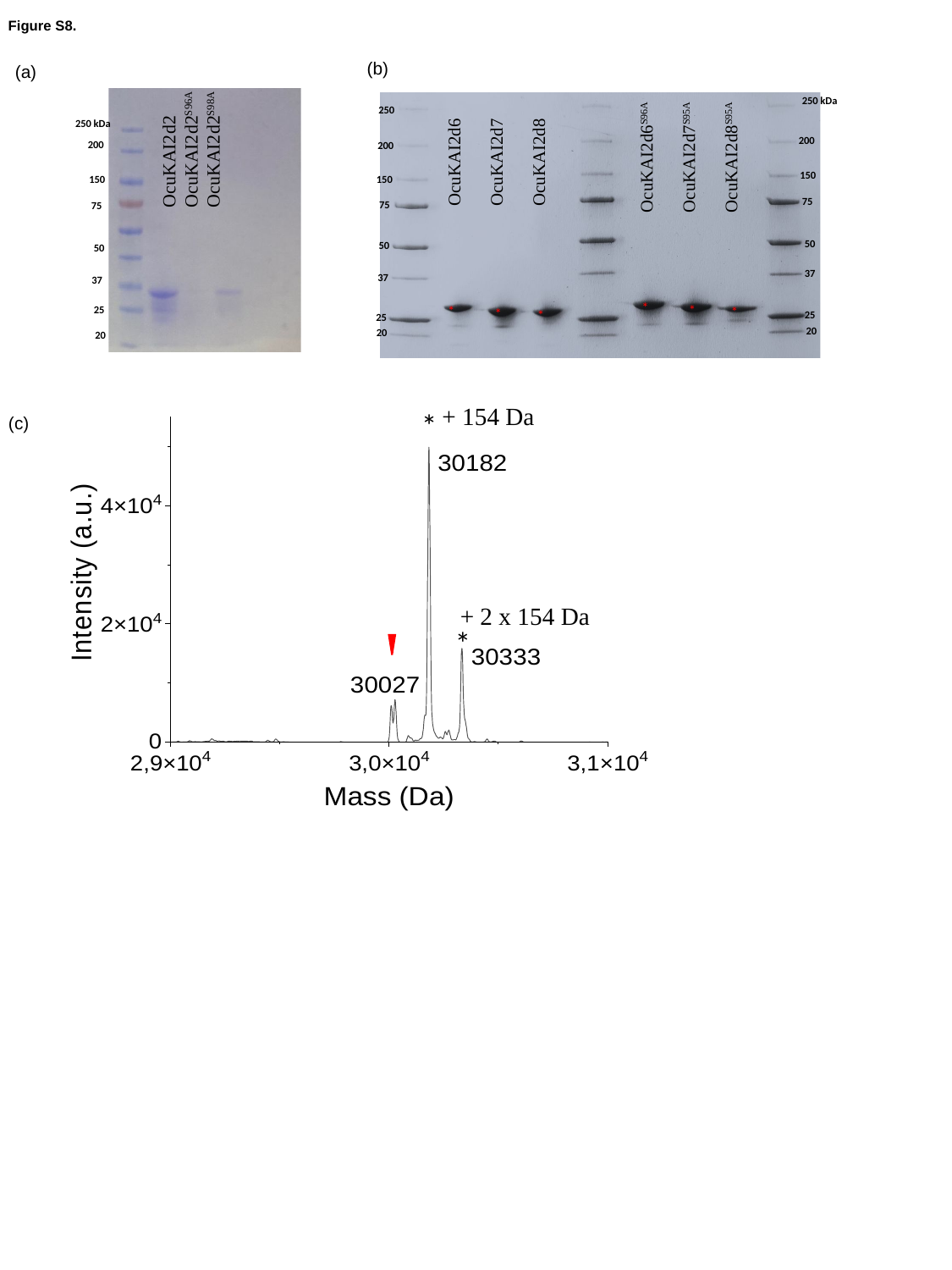

Figure S8.
OcuKAI2d2
OcuKAI2d2S96A
OcuKAI2d2S98A
(b)
(a)
OcuKAI2d6S96A
OcuKAI2d7S95A
OcuKAI2d8S95A
250 kDa
250
OcuKAI2d6
OcuKAI2d7
OcuKAI2d8
250 kDa
200
200
200
150
150
150
75
75
75
50
50
50
37
37
37
*
*
*
*
25
*
*
25
25
20
20
20
+ 154 Da
*
(c)
+ 2 x 154 Da
*

## Slide 10
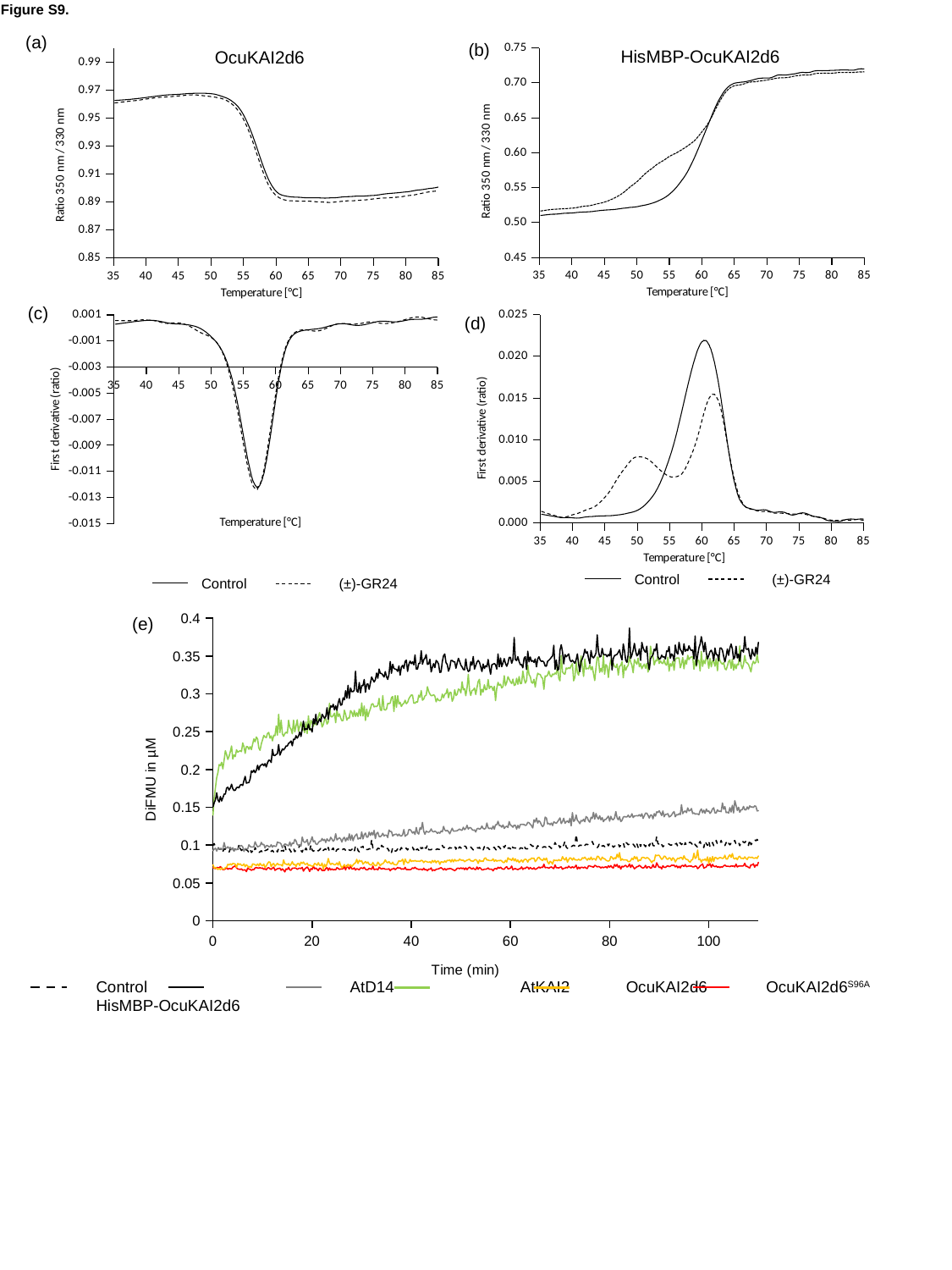

Figure S9.
(a)
(b)
### Chart
| Category | OcKAI2d6 10 µM + DMSO | OcKAI2d6 10 µM + (+/-)-GR24 400µM |
|---|---|---|
### Chart
| Category | HisMBP-OcKai2d6 10µM + DMSO | HisMBP-OcKai2d6 10µM + (+/-)-GR24 400µM |
|---|---|---|HisMBP-OcuKAI2d6
OcuKAI2d6
(c)
### Chart
| Category | OcKAI2d6 10 µM + DMSO | OcKAI2d6 10 µM + (+/-)-GR24 400µM |
|---|---|---|
### Chart
| Category | HisMBP-OcKai2d6 10µM + DMSO | HisMBP-OcKai2d6 10µM + (+/-)-GR24 400µM |
|---|---|---|(d)
Control (±)-GR24
Control (±)-GR24
### Chart
| Category | RMS3 (±)-GC240 | OcKAI2d6 (±)-GC240 | OcKAI2d6 S96A (±)-GC240 | HisMBP-OcKAI2d6 (±)-GC240 | AtKAI2 (±)-GC240 | Vide (±)-GC240 |
|---|---|---|---|---|---|---|(e)
Control 		AtD14	 AtKAI2	 OcuKAI2d6 	 OcuKAI2d6S96A	 HisMBP-OcuKAI2d6

## Slide 11
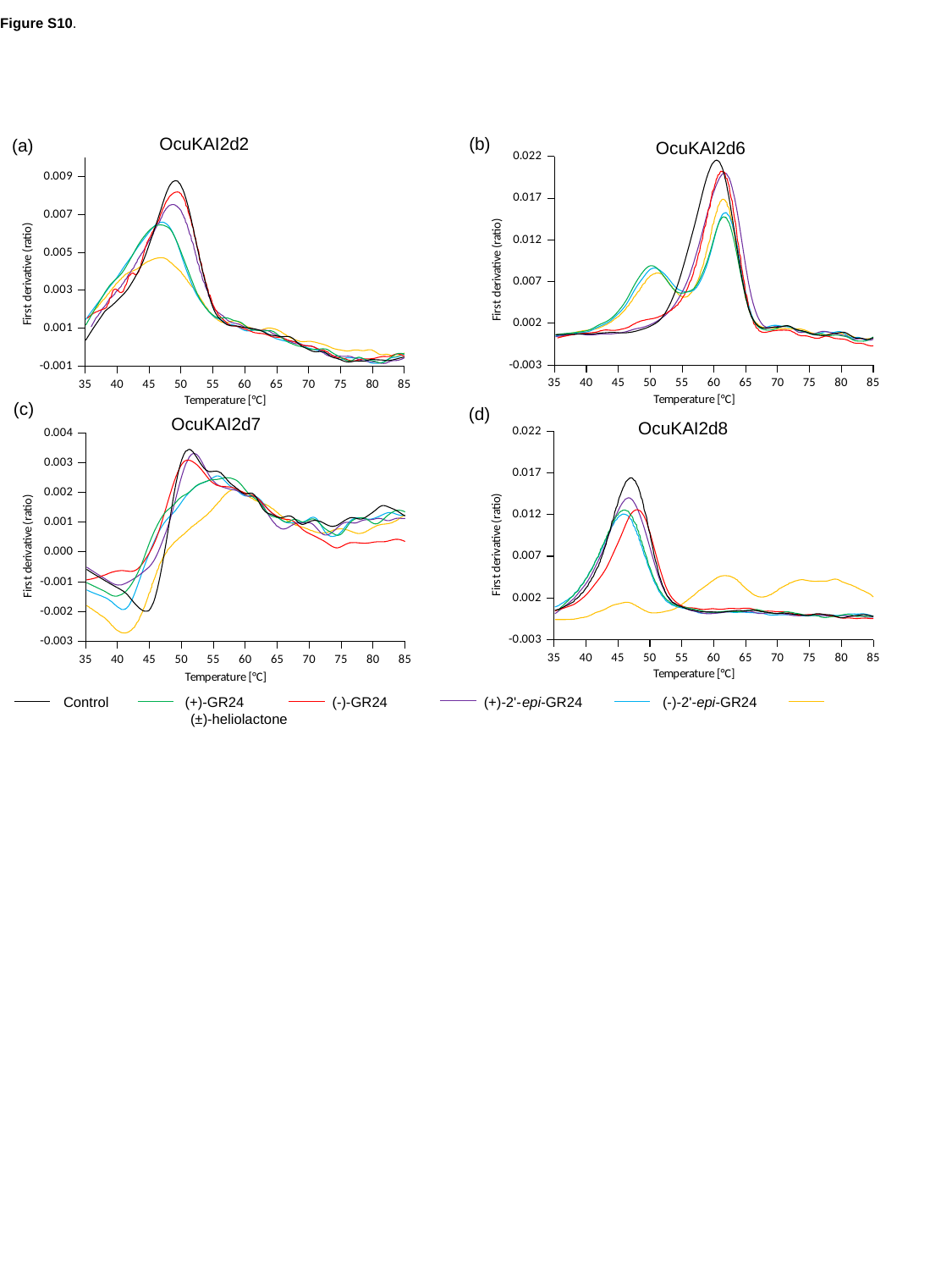

Figure S10.
OcuKAI2d2
(b)
(a)
 OcuKAI2d6
### Chart
| Category | OcKAI2d6 10µM + DMSO | OcKAI2d6 10µM + (+)-GR24 | OcKAI2d6 10µM + (-)-GR24 | OcKAI2d6 10µM + (+)-2'-epi-GR24 400 µM | OcKAI2d6 10µM + (-)-2'-epi-GR24 400 µM | OcKAI2d6 10 µM + heliolactone 400 µM |
|---|---|---|---|---|---|---|
### Chart
| Category | OcuKAI2d2 10µM DMSO | OcuKAI2d2 10µM (+)GR24 200µM | OcuKAI2d2 10µM (-)GR24 200µM | OcuKAI2d2 10µM (+)-Epi-GR24 200µM | OcuKAI2d2 10µM (-)-Epi-GR24 200µM | OcuKAI2d2 10µM (+/-)Heliola 200µM |
|---|---|---|---|---|---|---|(c)
(d)
OcuKAI2d7
OcuKAI2d8
### Chart
| Category | OcKAI2d8 10µM+ DMSO 400 µM | OcKAI2d8 10µM+ (+)-GR24 400 µM | OcKAI2d8 10µM+ (-)-GR24 400 µM | OcKAI2d8 10µM+ (+)-2'-epi-GR24 400 µM | OcKAI2d8 10µM+ (-)-2'-epi-GR24 | OcKAI2d8 10 µM + helio 400 µM |
|---|---|---|---|---|---|---|
### Chart
| Category | OcKAI2d7 10µM + DMSO | OcKAI2d7 10µM + (+)-GR24 400µM | OcKAI2d7 10µM + (-)-GR24 400µM | OcKAI2d7 10µM + (+)-2'-epi-GR24 400µM | OcKAI2d7 10µM + (-)-2'-epi-GR24 400µM | OcKAI2d7 10µM + Heliolactone |
|---|---|---|---|---|---|---|Control (+)-GR24 (-)-GR24 (+)-2'-epi-GR24 (-)-2'-epi-GR24 		(±)-heliolactone

## Slide 12
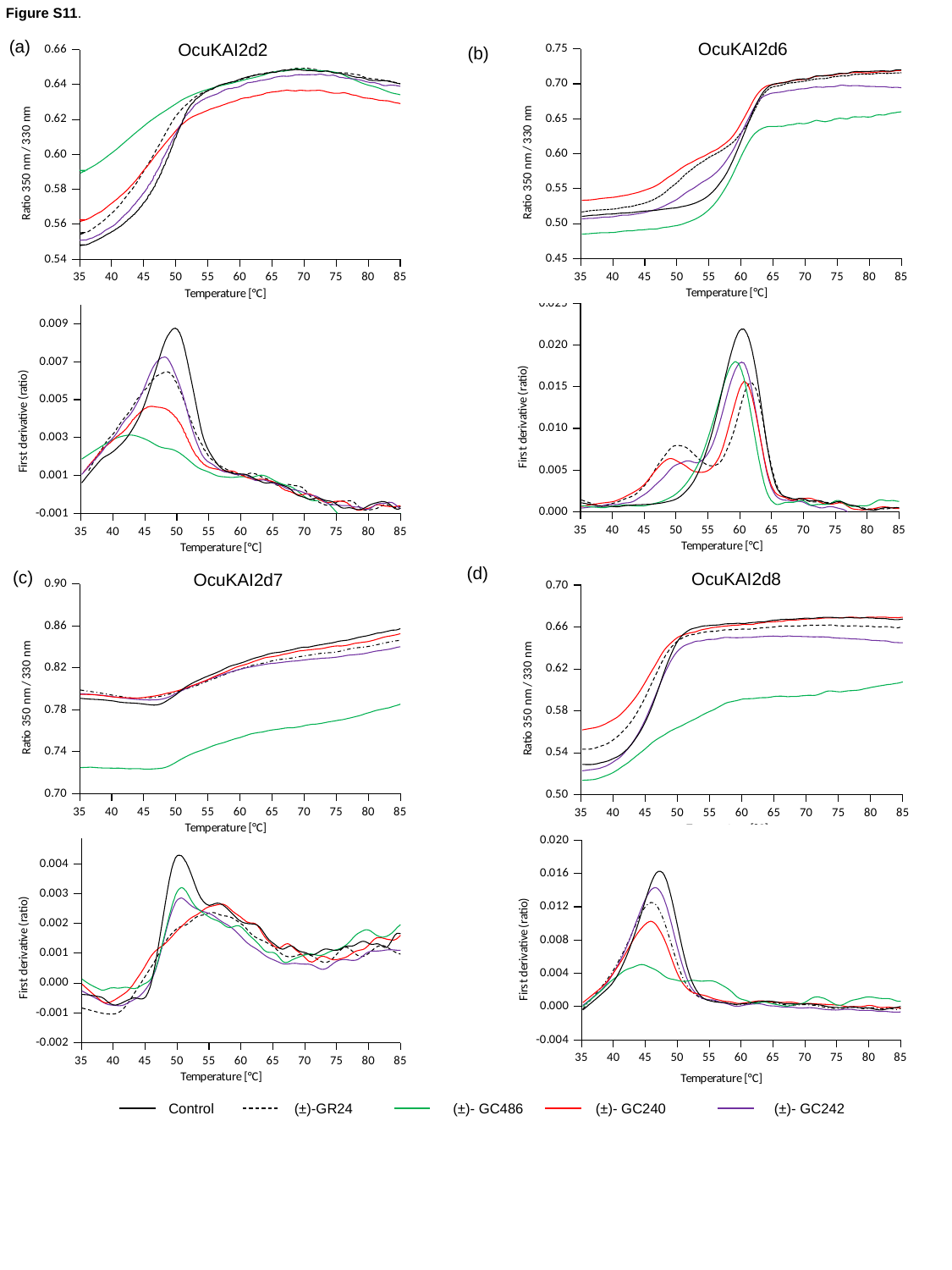

Figure S11.
(a)
 OcuKAI2d6
 OcuKAI2d2
(b)
### Chart
| Category | OcKAI2d6 10 µM + DMSO | OcKAI2d6 10 µM + GC 486 400µM | OcKAI2d6 10 µM + GC 240 400 µM | OcKAI2d6 10 µM + GC 242 400µM | OcKAI2d6 10 µM + (+/-)-GR24 400µM |
|---|---|---|---|---|---|
### Chart
| Category | OcuKAI2d2 10µM DMSO | OcuKAI2d2 10µM Gc242 100µM | OcuKAI2d2 10µM GC240 100µM | OcuKAI2d2 10µM Gc486 100µM | OcuKAI2d2 10µM (+/-)GR24 200µM |
|---|---|---|---|---|---|
### Chart
| Category | OcKAI2d6 10 µM + DMSO | OcKAI2d6 10 µM + GC 486 400µM | OcKAI2d6 10 µM + GC 240 400 µM | OcKAI2d6 10 µM + GC 242 400µM | OcKAI2d6 10 µM + (+/-)-GR24 400µM |
|---|---|---|---|---|---|
### Chart
| Category | OcuKAI2d2 10µM DMSO | OcuKAI2d2 10µM Gc242 100µM | OcuKAI2d2 10µM GC240 100µM | OcuKAI2d2 10µM Gc486 100µM | OcuKAI2d2 10µM (+/-)GR24 200µM |
|---|---|---|---|---|---|(d)
(c)
 OcuKAI2d8
 OcuKAI2d7
### Chart
| Category | OcKAI2d7 10 µM + DMSO | OcKAI2d7 10 µM + GC 486 400µM | OcKAI2d7 10 µM + GC 240 400 µM | OcKAI2d7 10 µM + GC 242 400µM | OcKAI2d7 10 µM + (+/-)-GR24 400µM |
|---|---|---|---|---|---|
### Chart
| Category | OcKAI2d8 10 µM + DMSO | OcKAI2d8 10 µM + GC 486 400µM | OcKAI2d8 10 µM + GC 240 400 µM | OcKAI2d8 10 µM + GC 242 400µM | OcKAI2d8 10 µM + (+/-)-GR24 400µM |
|---|---|---|---|---|---|
### Chart
| Category | OcKAI2d7 10 µM + DMSO | OcKAI2d7 10 µM + GC 486 400µM | OcKAI2d7 10 µM + GC 240 400 µM | OcKAI2d7 10 µM + GC 242 400µM | OcKAI2d7 10 µM + (+/-)-GR24 400µM |
|---|---|---|---|---|---|
### Chart
| Category | OcKAI2d8 10 µM + DMSO | OcKAI2d8 10 µM + GC 486 400µM | OcKAI2d8 10 µM + GC 240 400 µM | OcKAI2d8 10 µM + GC 242 400µM | OcKAI2d8 10 µM + (+/-)-GR24 400µM |
|---|---|---|---|---|---|Control (±)-GR24 (±)- GC486 (±)- GC240 (±)- GC242

## Slide 13
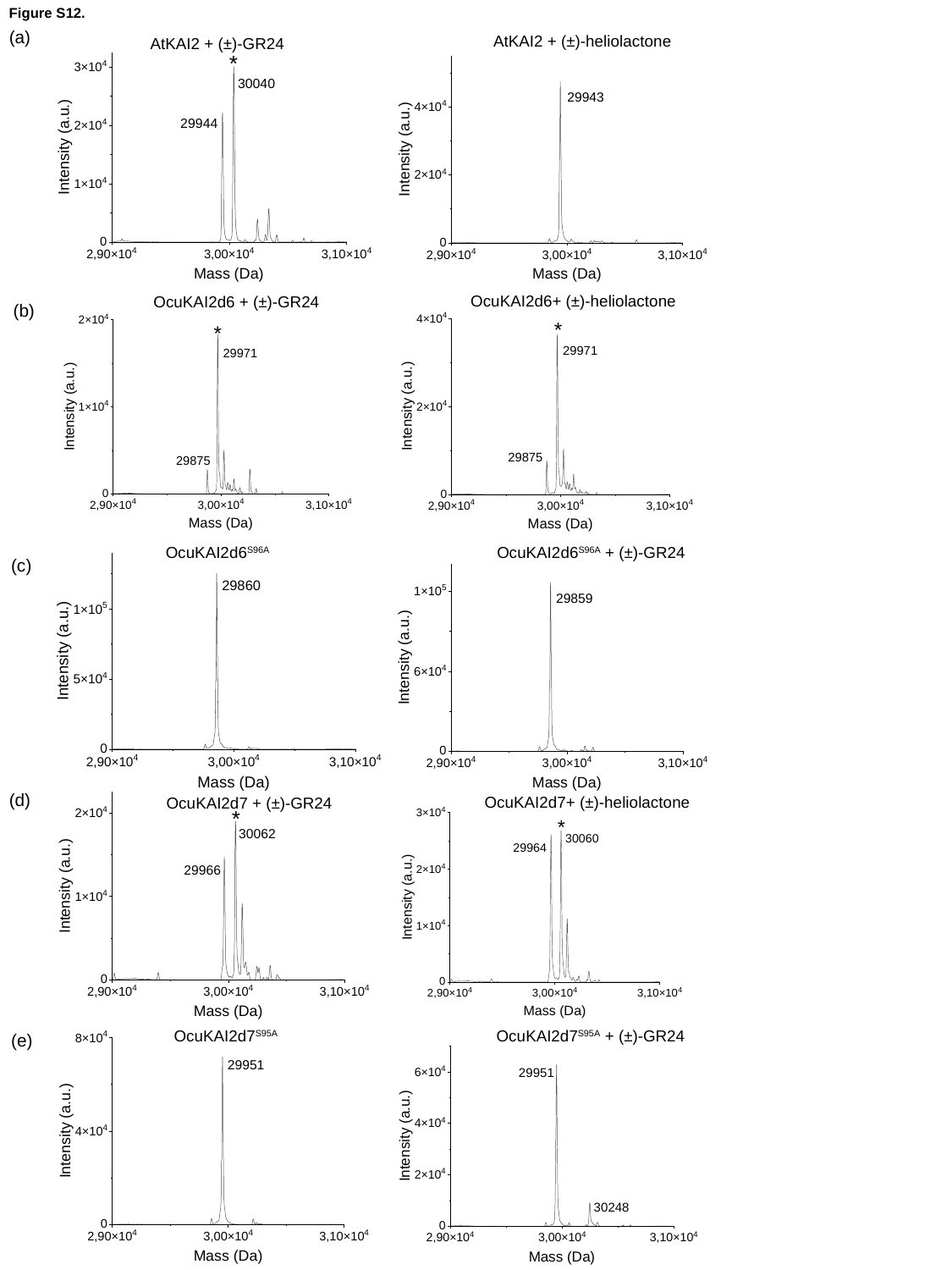

Figure S12.
(a)
AtKAI2 + (±)-heliolactone
AtKAI2 + (±)-GR24
OcuKAI2d6+ (±)-heliolactone
OcuKAI2d6 + (±)-GR24
(b)
OcuKAI2d6S96A + (±)-GR24
OcuKAI2d6S96A
(c)
(d)
OcuKAI2d7+ (±)-heliolactone
OcuKAI2d7 + (±)-GR24
OcuKAI2d7S95A + (±)-GR24
OcuKAI2d7S95A
(e)

## Slide 14
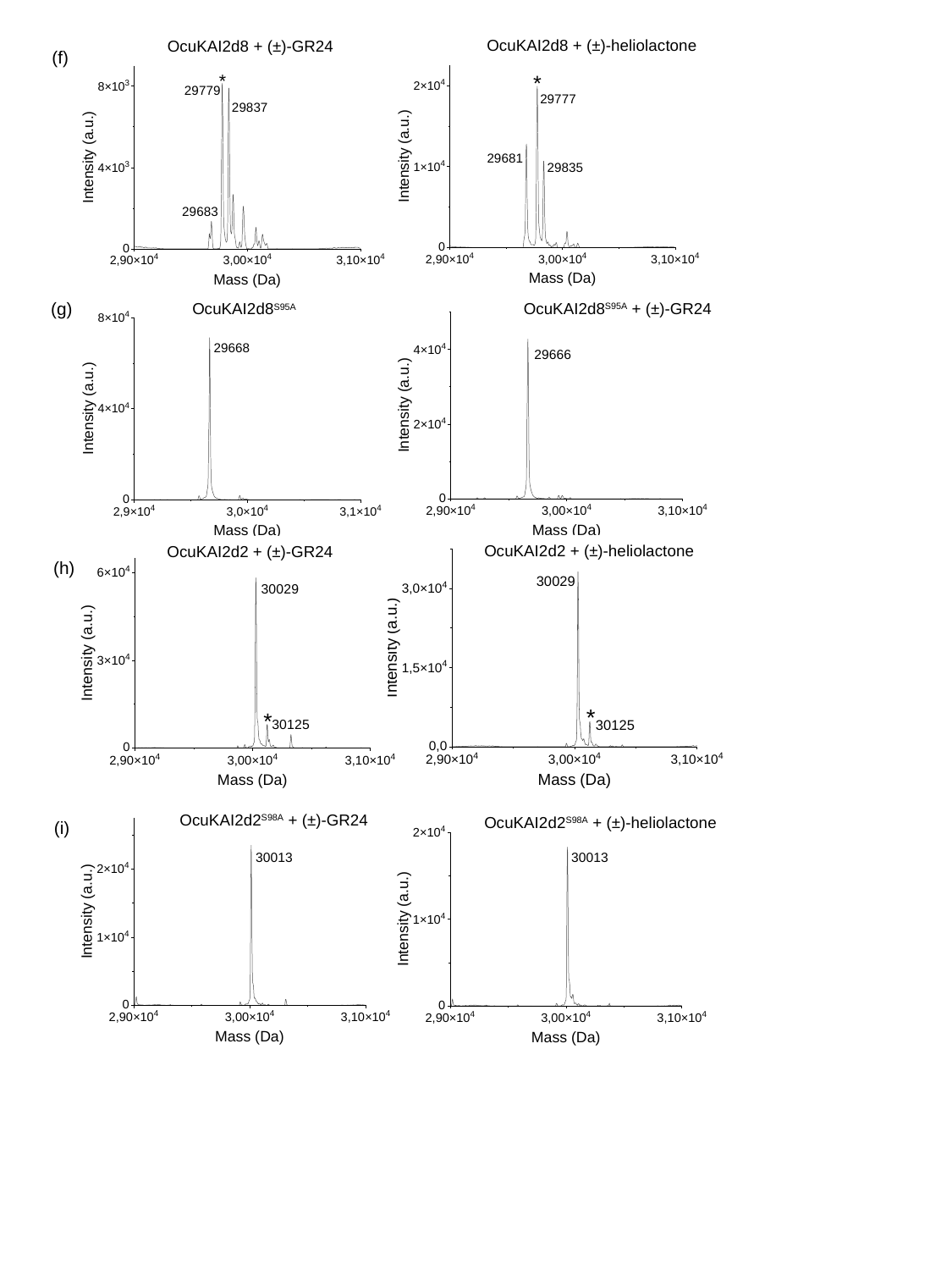

OcuKAI2d8 + (±)-heliolactone
OcuKAI2d8 + (±)-GR24
(f)
(g)
OcuKAI2d8S95A + (±)-GR24
OcuKAI2d8S95A
OcuKAI2d2 + (±)-heliolactone
OcuKAI2d2 + (±)-GR24
(h)
OcuKAI2d2S98A + (±)-GR24
OcuKAI2d2S98A + (±)-heliolactone
(i)

## Slide 15
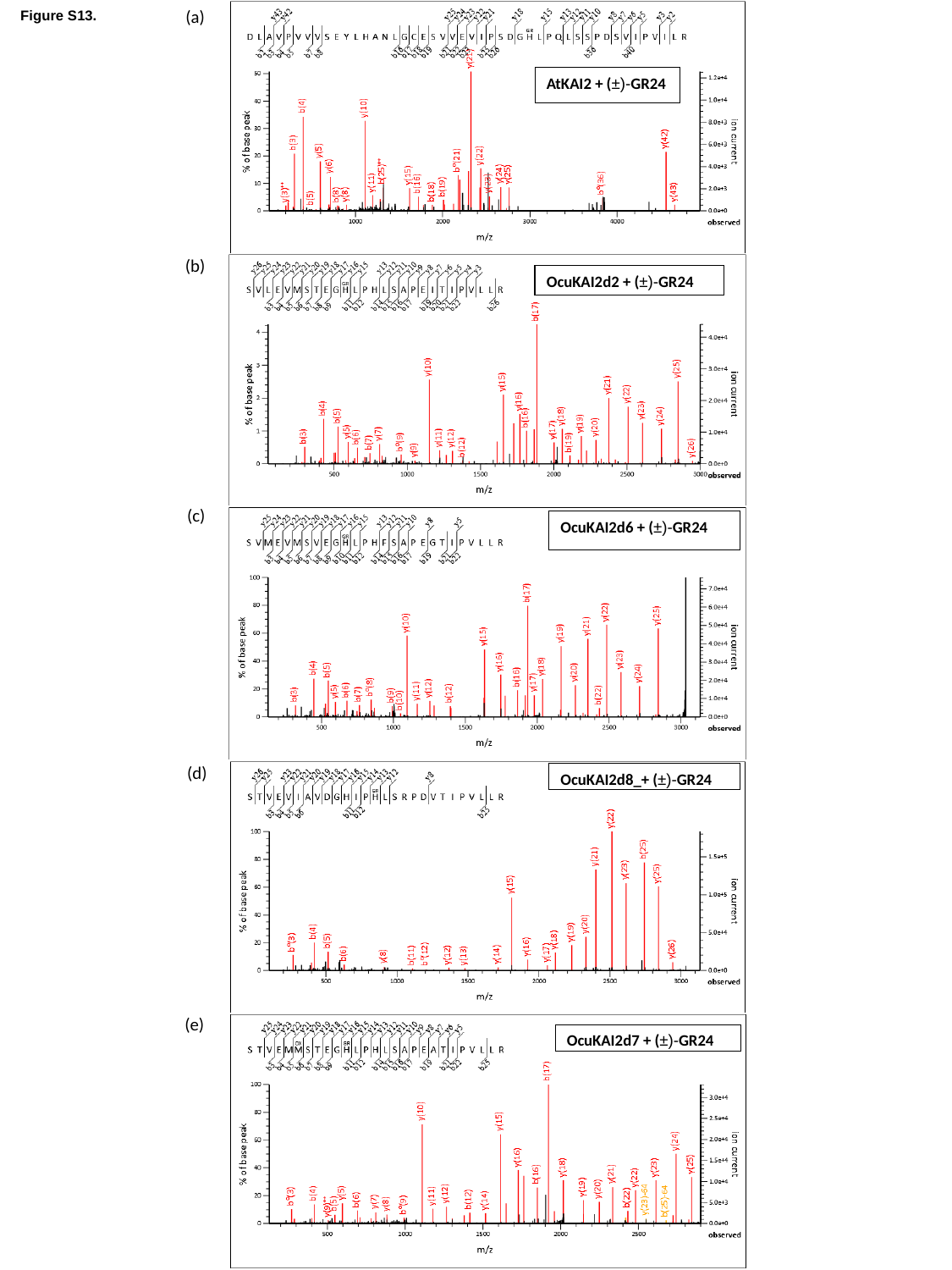

(a)
Figure S13.
AtKAI2 + (±)-GR24
(b)
OcuKAI2d2 + (±)-GR24
(c)
OcuKAI2d6 + (±)-GR24
(d)
OcuKAI2d8_+ (±)-GR24
(e)
OcuKAI2d7 + (±)-GR24

## Slide 16
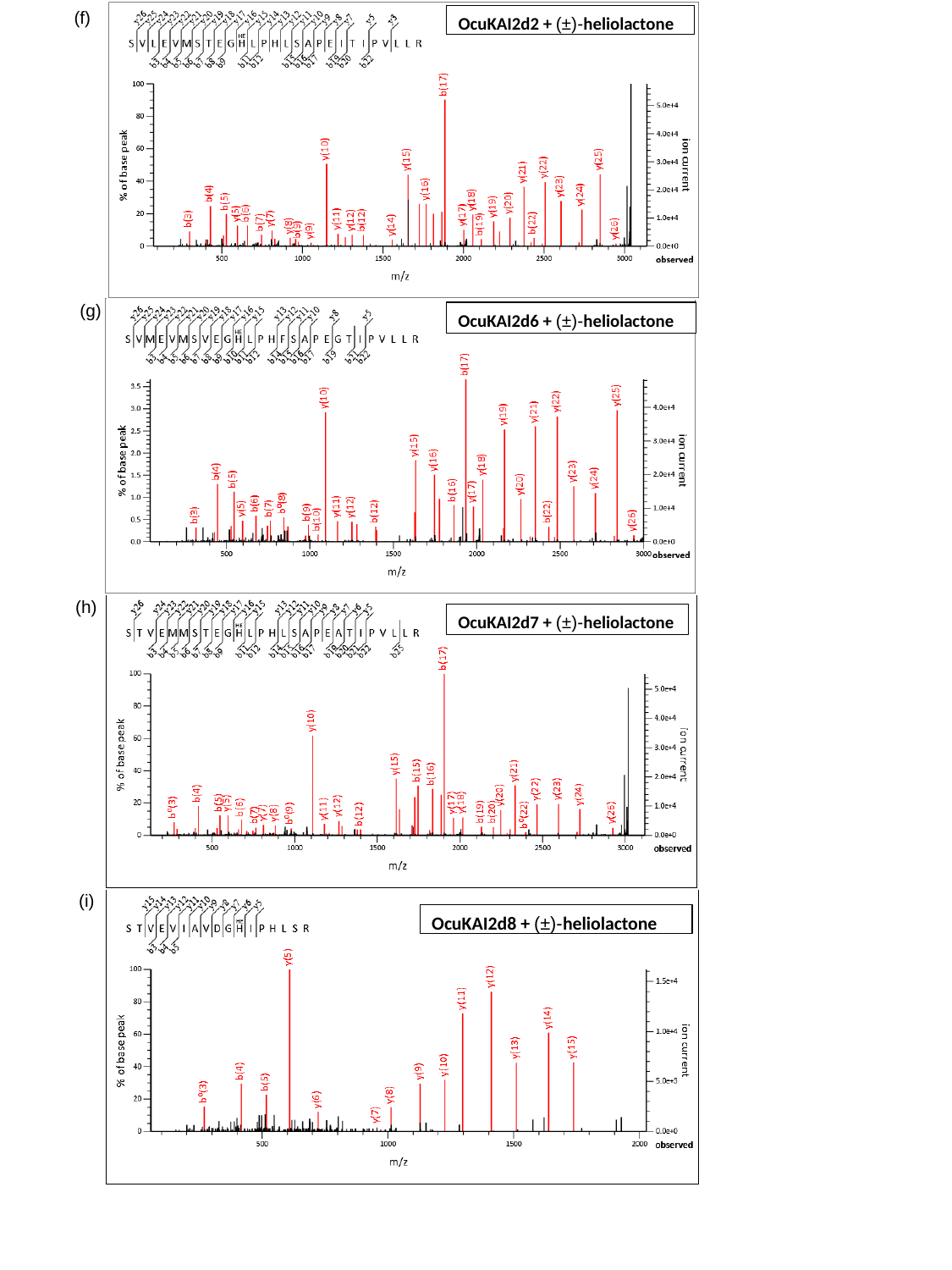

(f)
OcuKAI2d2 + (±)-heliolactone
(g)
OcuKAI2d6 + (±)-heliolactone
(h)
OcuKAI2d7 + (±)-heliolactone
(i)
OcuKAI2d8 + (±)-heliolactone

## Slide 17
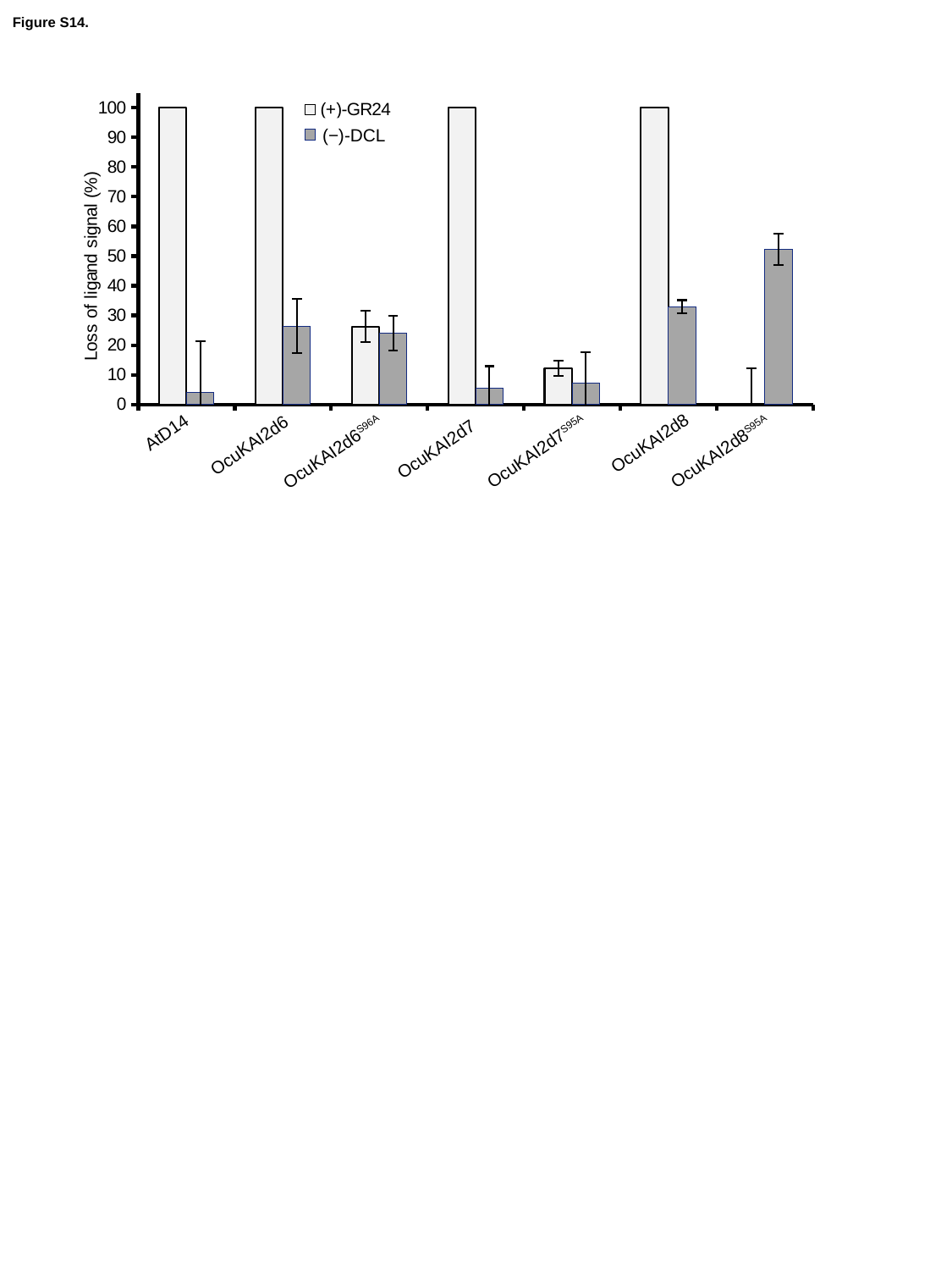

Figure S14.
### Chart
| Category | | |
|---|---|---|
| AtD14 | 100.0 | 3.973643247718144 |
| OcuKAI2d6 | 100.0 | 26.431419231381813 |
| OcuKAI2d6 S96A | 26.24988160798036 | 23.911123744839347 |
| OcuKAI2d7 | 100.0 | 5.442387839032908 |
| OcuKAI2d7 S96A | 12.096200613851869 | 7.245750291489216 |
| OcuKAI2d8 | 100.0 | 32.944795249763644 |
| OcuKAI2d8 S96A | -0.2770717520458324 | 52.38996695661857 |(−)-DCL
AtD14
OcuKAI2d8
OcuKAI2d7S95A
OcuKAI2d8S95A
OcuKAI2d6S96A
OcuKAI2d6
OcuKAI2d7

## Slide 18
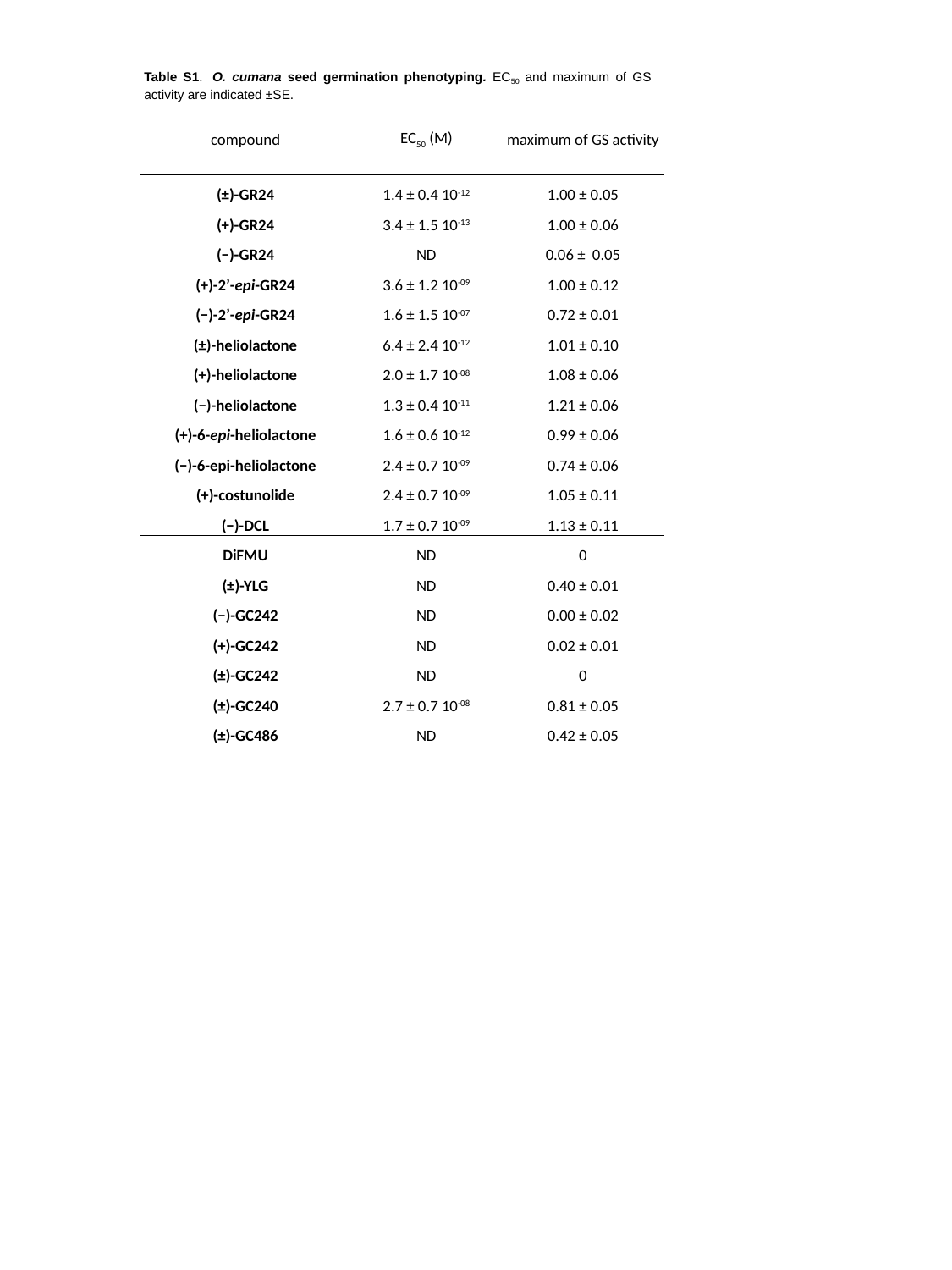

Table S1.  O. cumana seed germination phenotyping. EC50 and maximum of GS activity are indicated ±SE.
| compound | EC50 (M) | maximum of GS activity |
| --- | --- | --- |
| (±)-GR24 | 1.4 ± 0.4 10-12 | 1.00 ± 0.05 |
| (+)-GR24 | 3.4 ± 1.5 10-13 | 1.00 ± 0.06 |
| (−)-GR24 | ND | 0.06 ± 0.05 |
| (+)-2’-epi-GR24 | 3.6 ± 1.2 10-09 | 1.00 ± 0.12 |
| (−)-2’-epi-GR24 | 1.6 ± 1.5 10-07 | 0.72 ± 0.01 |
| (±)-heliolactone | 6.4 ± 2.4 10-12 | 1.01 ± 0.10 |
| (+)-heliolactone | 2.0 ± 1.7 10-08 | 1.08 ± 0.06 |
| (−)-heliolactone | 1.3 ± 0.4 10-11 | 1.21 ± 0.06 |
| (+)-6-epi-heliolactone | 1.6 ± 0.6 10-12 | 0.99 ± 0.06 |
| (−)-6-epi-heliolactone | 2.4 ± 0.7 10-09 | 0.74 ± 0.06 |
| (+)-costunolide | 2.4 ± 0.7 10-09 | 1.05 ± 0.11 |
| (−)-DCL | 1.7 ± 0.7 10-09 | 1.13 ± 0.11 |
| DiFMU | ND | 0 |
| (±)-YLG | ND | 0.40 ± 0.01 |
| (−)-GC242 | ND | 0.00 ± 0.02 |
| (+)-GC242 | ND | 0.02 ± 0.01 |
| (±)-GC242 | ND | 0 |
| (±)-GC240 | 2.7 ± 0.7 10-08 | 0.81 ± 0.05 |
| (±)-GC486 | ND | 0.42 ± 0.05 |

## Slide 19
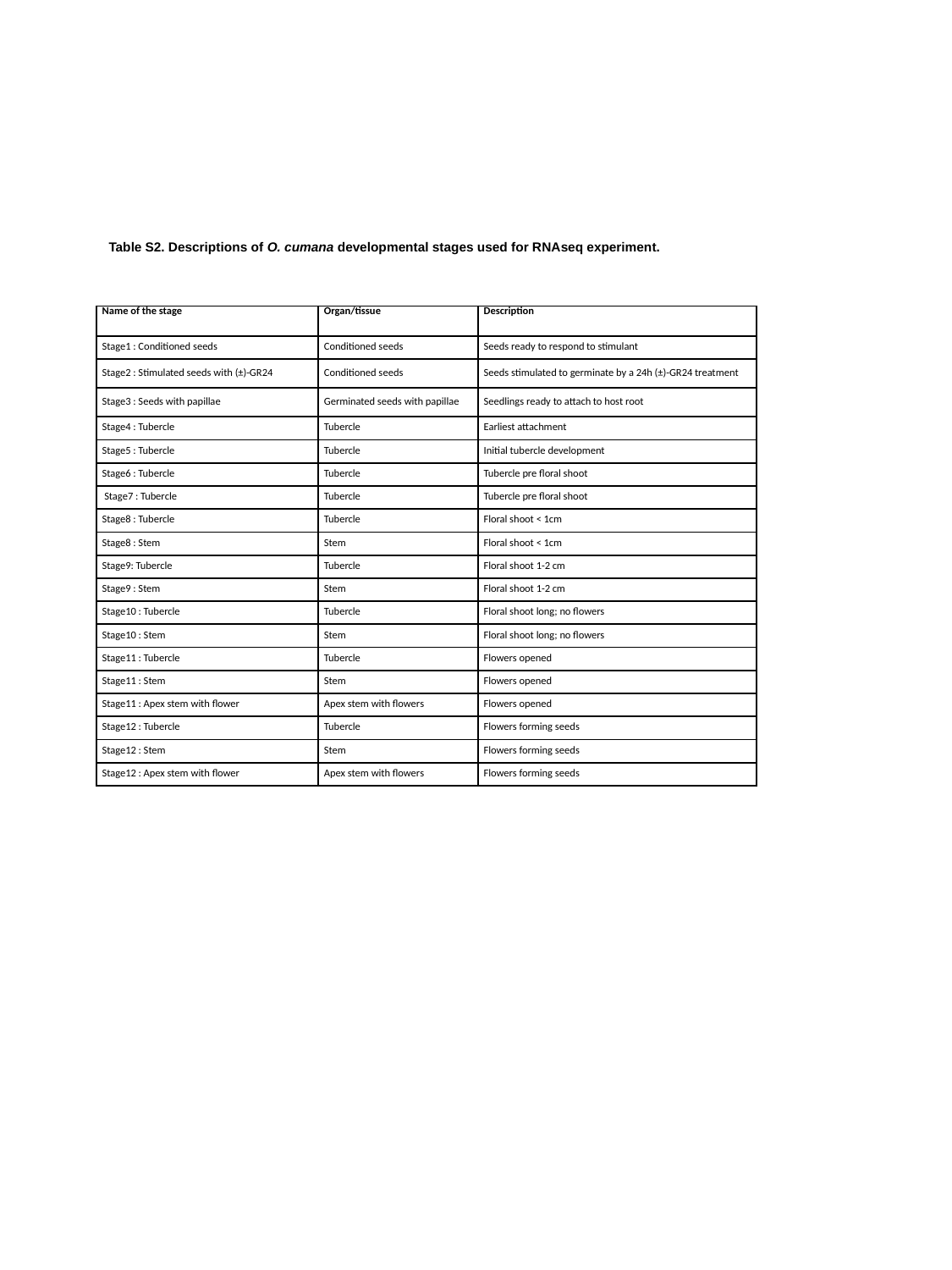

Table S2. Descriptions of O. cumana developmental stages used for RNAseq experiment.
| Name of the stage | Organ/tissue | Description |
| --- | --- | --- |
| Stage1 : Conditioned seeds | Conditioned seeds | Seeds ready to respond to stimulant |
| Stage2 : Stimulated seeds with (±)-GR24 | Conditioned seeds | Seeds stimulated to germinate by a 24h (±)-GR24 treatment |
| Stage3 : Seeds with papillae | Germinated seeds with papillae | Seedlings ready to attach to host root |
| Stage4 : Tubercle | Tubercle | Earliest attachment |
| Stage5 : Tubercle | Tubercle | Initial tubercle development |
| Stage6 : Tubercle | Tubercle | Tubercle pre floral shoot |
| Stage7 : Tubercle | Tubercle | Tubercle pre floral shoot |
| Stage8 : Tubercle | Tubercle | Floral shoot < 1cm |
| Stage8 : Stem | Stem | Floral shoot < 1cm |
| Stage9: Tubercle | Tubercle | Floral shoot 1-2 cm |
| Stage9 : Stem | Stem | Floral shoot 1-2 cm |
| Stage10 : Tubercle | Tubercle | Floral shoot long; no flowers |
| Stage10 : Stem | Stem | Floral shoot long; no flowers |
| Stage11 : Tubercle | Tubercle | Flowers opened |
| Stage11 : Stem | Stem | Flowers opened |
| Stage11 : Apex stem with flower | Apex stem with flowers | Flowers opened |
| Stage12 : Tubercle | Tubercle | Flowers forming seeds |
| Stage12 : Stem | Stem | Flowers forming seeds |
| Stage12 : Apex stem with flower | Apex stem with flowers | Flowers forming seeds |

## Slide 20
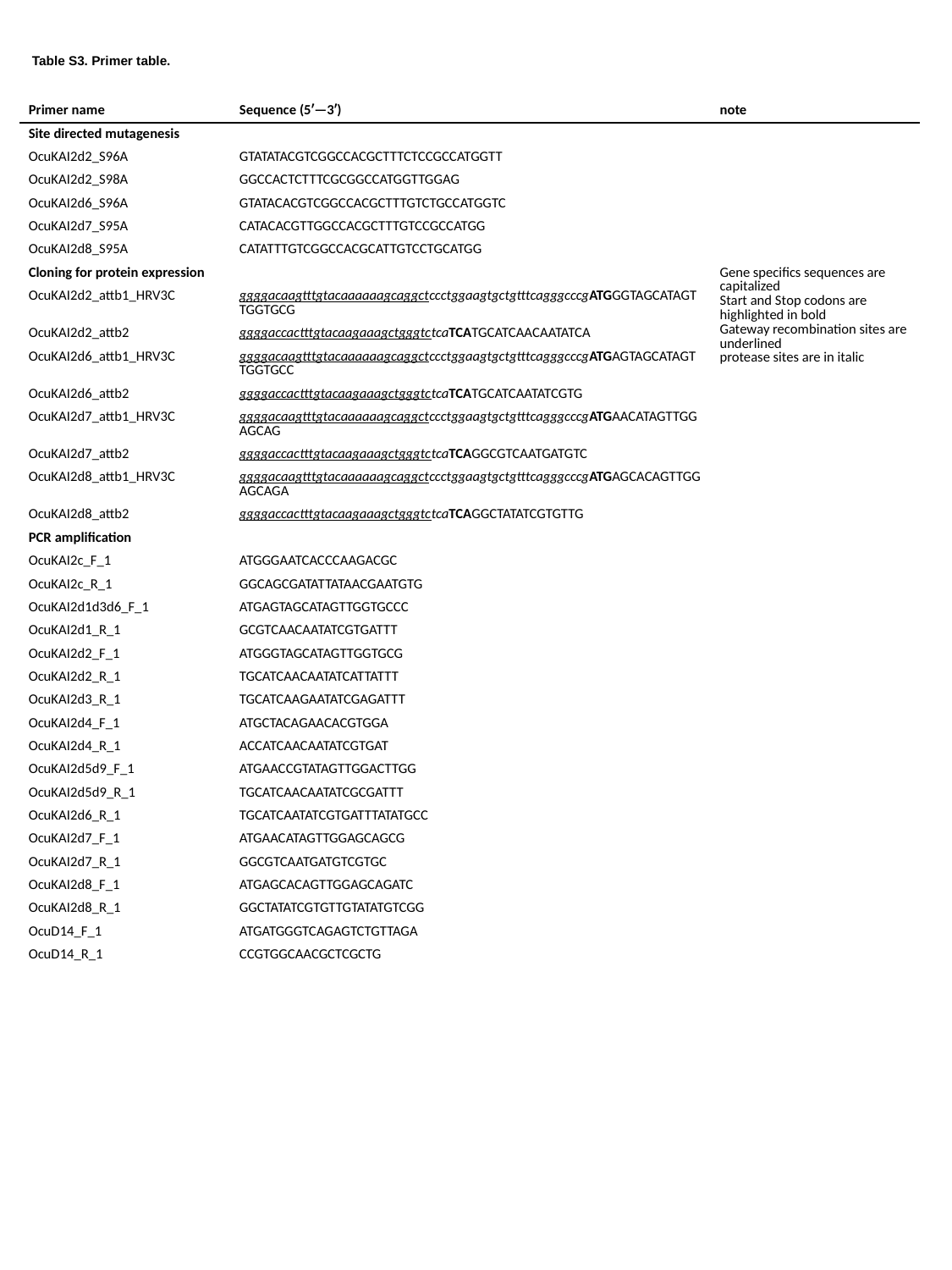

Table S3. Primer table.
| Primer name | Sequence (5ʹ—3ʹ) | note |
| --- | --- | --- |
| Site directed mutagenesis | | |
| OcuKAI2d2\_S96A | GTATATACGTCGGCCACGCTTTCTCCGCCATGGTT | |
| OcuKAI2d2\_S98A | GGCCACTCTTTCGCGGCCATGGTTGGAG | |
| OcuKAI2d6\_S96A | GTATACACGTCGGCCACGCTTTGTCTGCCATGGTC | |
| OcuKAI2d7\_S95A | CATACACGTTGGCCACGCTTTGTCCGCCATGG | |
| OcuKAI2d8\_S95A | CATATTTGTCGGCCACGCATTGTCCTGCATGG | |
| Cloning for protein expression | | Gene specifics sequences are capitalized Start and Stop codons are highlighted in bold Gateway recombination sites are underlined protease sites are in italic |
| OcuKAI2d2\_attb1\_HRV3C | ggggacaagtttgtacaaaaaagcaggctccctggaagtgctgtttcagggcccgATGGGTAGCATAGTTGGTGCG | |
| OcuKAI2d2\_attb2 | ggggaccactttgtacaagaaagctgggtctcaTCATGCATCAACAATATCA | |
| OcuKAI2d6\_attb1\_HRV3C | ggggacaagtttgtacaaaaaagcaggctccctggaagtgctgtttcagggcccgATGAGTAGCATAGTTGGTGCC | |
| OcuKAI2d6\_attb2 | ggggaccactttgtacaagaaagctgggtctcaTCATGCATCAATATCGTG | |
| OcuKAI2d7\_attb1\_HRV3C | ggggacaagtttgtacaaaaaagcaggctccctggaagtgctgtttcagggcccgATGAACATAGTTGGAGCAG | |
| OcuKAI2d7\_attb2 | ggggaccactttgtacaagaaagctgggtctcaTCAGGCGTCAATGATGTC | |
| OcuKAI2d8\_attb1\_HRV3C | ggggacaagtttgtacaaaaaagcaggctccctggaagtgctgtttcagggcccgATGAGCACAGTTGGAGCAGA | |
| OcuKAI2d8\_attb2 | ggggaccactttgtacaagaaagctgggtctcaTCAGGCTATATCGTGTTG | |
| PCR amplification | | |
| OcuKAI2c\_F\_1 | atgggaatcacccaagacgc | |
| OcuKAI2c\_R\_1 | ggcagcgatattataacgaatgtg | |
| OcuKAI2d1d3d6\_F\_1 | atgagtagcatagttggtgccc | |
| OcuKAI2d1\_R\_1 | gcgtcaacaatatcgtgattt | |
| OcuKAI2d2\_F\_1 | atgggtagcatagttggtgcg | |
| OcuKAI2d2\_R\_1 | tgcatcaacaatatcattattt | |
| OcuKAI2d3\_R\_1 | tgcatcaagaatatcgagattt | |
| OcuKAI2d4\_F\_1 | atgctacagaacacgtgga | |
| OcuKAI2d4\_R\_1 | accatcaacaatatcgtgat | |
| OcuKAI2d5d9\_F\_1 | atgaaccgtatagttggacttgg | |
| OcuKAI2d5d9\_R\_1 | tgcatcaacaatatcgcgattt | |
| OcuKAI2d6\_R\_1 | tgcatcaatatcgtgatttatatgcc | |
| OcuKAI2d7\_F\_1 | atgaacatagttggagcagcg | |
| OcuKAI2d7\_R\_1 | ggcgtcaatgatgtcgtgc | |
| OcuKAI2d8\_F\_1 | atgagcacagttggagcagatc | |
| OcuKAI2d8\_R\_1 | ggctatatcgtgttgtatatgtcgg | |
| OcuD14\_F\_1 | atgatgggtcagagtctgttaga | |
| OcuD14\_R\_1 | ccgtggcaacgctcgctg | |
