# Supplementary material for: Strigolactones and sesquiterpene lactones induce Orobanche cumana germination via KAI2d receptors through distinct processes
Source: Plant J. 2026 Apr 16;126(1):e70852. doi: 10.1111/tpj.70852 (PMC13086240; doi:10.1111/tpj.70852)
Supplement: Supplementary file 3 — Data S1. Supplemental information regarding the synthesis of chemical compounds. [file TPJ-126-0-s003.docx]

**Supplementary information**

**Chemicals.** GR24 and isomers were prepared according to (de Saint Germain *et al.*, 2019). GC probes were synthesized according to (de Saint Germain *et al.*, 2016) and are commercially available as StrigoLightOn^TM^ probes (<https://www.idylle-labs.com/>). Sesquiterpenes lactones (DCL, costunolide) are commercially available from Sigma-Aldrich.


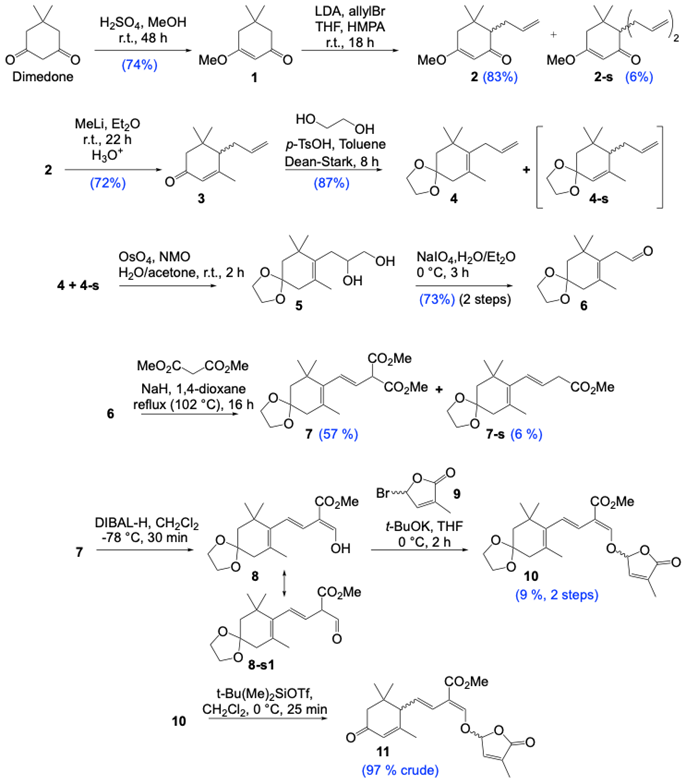


**Supplemental data 1. Synthesis of (±)-heliolactone.**

**Chemical synthesis of** (±)-**heliolactone and** (±)-**6-*epi-*heliolactone.** (±)-Heliolactone and (±)-6-*epi-*heliolactone were synthesized following the methodology of Yamamoto and colleagues (Yamamoto *et al.*, 2020), described from commercially available dimedone, avoiding using stannane intermediates as described in (Woo and McErlean, 2019, Yoshimura *et al.*, 2019) (Supplemental data 1). The ketone **1** yield was obtained with a 74 % reaction by dimedone with H_2_SO_4_ in MeOH. Allylation of this ketone **1** using lithium diisopropyl amide (LDA) base and allyl bromide gave compound **2** in 83 % yield, and the corresponding di-allylated byproduct **2-s** in 6 % yield. The treatment of compound **2** with MeLi and then acidic work-up gave the enone **3** with 72 % yield. A keto protection of compound **3** by an acetal followed by an olefin migration afforded to the mixture of compound **4** and a minor rate of its inseparable regioisomer **4-s** in 87 % yield. A two-step oxidative cleavage was performed on compound **4** to obtain the corresponding aldehyde **6** in 81 % yield. A Knoevenagel-type condensation of aldehyde **6** with dimethyl malonate allowed obtaining the diester **7** and the corresponding mono-decarboxylated byproduct **7-s** in 57 % and 6 % yields respectively. Mono-reduction of this diester **7** using DIBAL-H, followed by an enol etherification with compound **9**, or compound **15** for the deuterated derivative, gave compound **10** or **21** with very low yields (9 % and 5 % respectively from the diester **7**). The last step of deprotection-isomerization was performed using *tert*-butyldimethylsilyl trifluoromethanesulfonate (TBDMS triflate) with good yields. Compound **11** was obtained as a mixture of enantiomers and diastereomers that could not be separated by conventional column chromatography. Several chiral separation conditions screening by supercritical fluid chromatography (SFC) allowed the separation of each isomer (see below). A couple of enantiomers were identified by ^1^H NMR analysis (identical ^1^H NMR spectra) as follows: products at retention time (RT) = 32.5 and 53.1 mins are enantiomers (couple 2), and products at 35.7 and 42.5 mins are enantiomers (couple 2) (Supplemental data 2). Comparison with ^1^H NMR spectra described in (Woo and McErlean, 2019, Yoshimura *et al.*, 2019) revealed that the couple 1 corresponds to natural heliolactone and the couple 2 corresponds to natural (recently described) 6-*epi*-heliolactone. Optical rotatory measures (values in experimental section) of the four products permitted us to finally identify (-)-heliolactone ([6*S*,11*R*], RT = 32.5 min), (+)-6-*epi*-heliolactone ([6*R*,11*R*], RT = 35.7 min), (-)-6-*epi*-helilactone ([6*S*,11*S*], RT = 42.5 min) and (+)-heliolactone ([6*R*,11*S*], RT = 53.1 min).


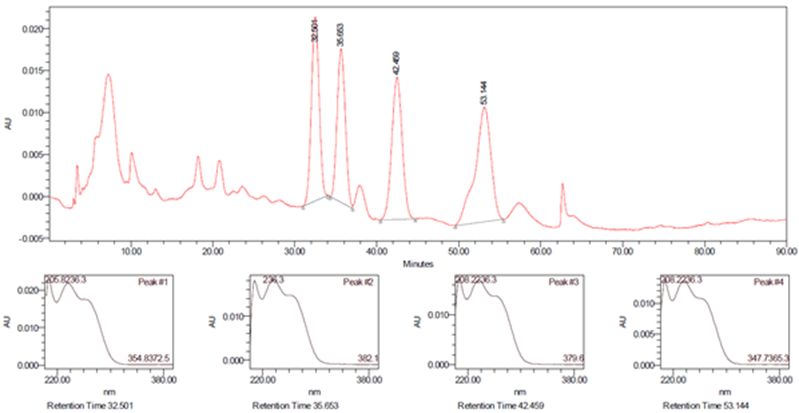


**Supplemental data 2. Chromatogram of the separation of heliolactone isomers by SFC.**

**Experimental section**

**General procedure.** Air- or/and water-sensitive reactions were carried out using commercial anhydrous solvents preserved on molecular sieves and under an argon/nitrogen atmosphere. Nuclear Magnetic Resonance (NMR) spectra [^1^H (300 MHz) and ^13^C (75 MHz)] were performed with Bruker Advance spectrometers at 25 °C using CDCl_3_ or C_6_D_6_ as solvents. Chemical shifts (δ) and coupling constants (*J*) are provided in ppm and Hertz (Hz), respectively. The solvent signal was used as an internal standard. CDCl_3_: δ (^1^H) = 7.26 ppm and δ (^13^C) = 77.16 ppm; C_6_D_6_: δ (^1^H) = 7.15 ppm and δ (^13^C) = 128.0 ppm. For the ^1^H NMR spectra, data are reported as follows: chemical shift, integration, multiplicity (s = singlet, d = doublet, t = triplet, q = quartet, m= multiplet, b = broad), and coupling constant. Analytical thin-layer chromatography (TLC) was carried out on 0.20 mm Macherey-Nager (MN) silica gel 60 pre-coated sheets, visualized under UV light (254 nm) and revealed with sulfuric vanillin reagent. Column chromatography was performed with a Pure Chromatography Instrument C-805 equipped with an UV detector using MN (50 µm irregular) (40-63 µm) or Buchi’s prepacked silica gel. Solvents and reagents were purchased from Sigma Aldrich, Alpha Aesar, Thermo Scientific, Accros, Fluka Prolabo, and Carlo Erba.

**3-Methoxy-5,5-dimethyl-2-cyclohexen-1-one (1).** To a suspension of commercially available dimedone (7.61 g, 54.29 mmol) in MeOH (30 mL) was added concentrated solution of H_2_SO_4_ (3.0 mL) at r.t. and the reaction mixture was stirred for 48 h to this temperature. Then Et_2_O (60 mL) and H_2_O (100 mL) were added and the resulting mixture was neutralized with solid NaHCO_3_ (16.5 g) to pH 7. The organic layer was separated and the aqueous layer was extracted thrice with EtOAc (50 mL). The combined organic layers were dried over Na_2_SO4, filtered and the solvents were evaporated under vacuum to furnish an orange-oil crude product (7.31 g, 87 %). This was then purified by distillation (b.p. 65 °C, 0.42 mm Hg) to obtain compound **1** (6.22 g, 40.33 mmol, 74 %) as a colorless oil. ^1^H NMR (CDCl_3_, 300 MHz): δ_H_ (ppm) = 5.35 (1H, s); 3.68 (3H, s); 2.26 (2H, s); 2.20 (2H, s); 1.06 (6H, s). These data were in accordance with the literature (Yoshino *et al.*, 2006).

**6-Allyl-3-methoxy-5,5-dimethylcyclohex-2-enone (2).** To a solution of iPr_2_NH (1.84 mL, 1.35 g, 13.31 mmol) in anhydrous THF (20 mL) was added *n*-BuLi (1.6 M, hexanes, 8.32 mL, 13.31 mmol) at 0 °C and the reaction mixture was allowed to warm to r.t. with stirring for 1 h. Then, a solution of compound **1** (1.35 g, 8.75 mmol) in anhydrous THF (6.8 mL) was added at -78 °C. After stirring for 1 h, a solution of allyl bromide (1.13 mL, 1.58 g, 13.08 mmol) in anhydrous THF (13.5 mL) and anhydrous HMPA (1.89 mL, 1.95 g, 10.86 mmol) were successively added at -78 °C. The reaction mixture was allowed to warm to r.t. with stirring for 18 h. Then, the reaction was quenched with a cooled saturated aqueous solution of NH_4_Cl (50 mL). The organic layer was separated, and the aqueous layer was extracted thrice with EtOAc (30 mL). The combined organic layers were washed with NH_4_Cl aq. sat. (2 x 30 mL) then brine (2 x 30 mL) then dried over Na_2_SO_4_, filtered and evaporated under reduce pressure. The residue was purified by column chromatography on silica gel using heptane/EtOAc (84:16) to give compound **2** (1.29 g, 6.64 mmol, 83 %) as a pale-yellow oil and the diallylated compound **2-s** as an orange solid (119.2 mg, 5.09 mmol, 6 %). **2**:  ^1^H NMR (CDCl_3_, 300 MHz): δ_H_ (ppm) = 5.92, 5.86 and 5.83 (all for 1H, successively t, *J* = 7.0, q, *J* = 7.0 and t, *J* = 7.0 Hz); 5.30 (1H, s); 5.04-4.93 (2H, m); 3.67 (3H, s); 2.35-2.18 (4H, m); 2.08 (1H, dd, *J* = 5.0, 8.5 Hz); 1.08 (3H, s); 0.99 (3H, s). These data were in accordance with Yamamoto et al. (2020). **2-s:** ^1^H NMR (CDCl_3_, 300 MHz): δ_H_ (ppm) = 5.88-8.82 (2H, m); 5.29 (1H, s); 5.05-4.96 (4H, m); 3.66 (3H, s); 2.53-2.45 (2H, m); 2.35 (2H, dd, *J* = 8.0, 15.0 Hz); 1.56 (2H, s); 1.09 (6H, s).

**4-Allyl-3,5,5-trimethylcyclohex-2-enone (3).** Under argon atmosphere and in anhydrous conditions, a solution of compound **2** (1.07 g, 5.51 mmol) in Et_2_O (14 mL) was added to a solution of MeLi (1.6 M in Et_2_O, 5.15 mL, 8.24 mmol) at -78 °C. The reaction mixture was allowed to warm at r.t. with stirring for 22 h. Then, the reaction was quenched with an aqueous saturated NH_4_Cl solution (15 mL) and EtOAc (15 mL) was added. The organic layer was separated, and the aqueous layer was extracted with EtOAc (2 x 15 mL). The combined organic layer was dried over Na_2_SO_4_, filtered, and concentrated under vacuum. The crude product (1.11 g) was purified by silica gel column chromatography using heptane/EtOAc (90:10) to give compound **3** (711.8 mg, 3.99 mmol, 72 %) as a pale-yellow oil. ^1^H NMR (CDCl_3_, 300 MHz): δ_H_ (ppm) = 5.88-5.74 (2H, m); 5.10-5.09, 5.05-5.03 and 5.00-4.99 (all for 2H, m); 2.55-2.46 (1H, m); 2.41 (1H, bd, *J* =17.5 Hz); 2.30-2.21 (1H, m); 2.04-1.99 (2H, m); 1.97 (3H, bd, *J* = 1.0 Hz); 1.07 (3H, s); 1.01 (3H, s). These data were in accordance with Yamamoto et al. (2020).

**8-Allyl-7,9,9-trimethyl-1,4-dioxaspiro[4.5]dec7-ene (4).** To a solution of compound **3** (787.4 mg, 4.42 mmol) in toluene (40 mL) were successively added ethylene glycol (12 mL, 13.3g, 214.6 mmol) and *p*-TsOH.H_2_O (66.0 mg, 0.35 mmol) at room temperature. The reaction mixture was then heated at reflux (140 °C) for 14 h using Dean-Stark system. After cooling to r.t., the reaction mixture was quenched with an aqueous solution of NaHCO_3_ sat. (40 mL) and extracted with EtOAc (3x 20 mL). The combined organic layer was dried over Na_2_SO_4_, filtered, and concentrated under vacuum. The crude mixture was purified by column chromatography on silica gel eluted with heptane/EtOAc (95:5) to give the compound **4** (756.8 mg, 3.40 mmol, 87 %) as a colorless oil. ^1^H NMR (CDCl_3_, 300 MHz): δ_H_ (ppm) = 5.81, 5.76 and 5.72 (all for 1H, successively t, *J* = 6.0, q, *J* = 6.0 and t, *J* = 6.0 Hz); 4.99 (1H, qd, *J* = 2.0, 8.0 Hz); 4.95 (1H, t, *J* = 2.0 Hz); 3.95 (4H, s); 2.83 (2H, d, *J* = 6.0 Hz); 2.26 (2H, d, J = 1.0 Hz); 1.67 (2H, s); 1.61 (3H, s); 1.07 (6H, s). These data were in accordance with Yamamoto et al. (2020).

**3-(7,9,9-Trimethyl-1,4-dioxaspiro[4.5]dec-7-en-8-yl)propane-1,2-diol (5).** To a stirred solution of compound **4** (505.3 mg, 2.27 mmol) in acetone (10 mL) and H_2_O (10 mL) were added OsO_4_ (4%, aq., 690 µL) and NMO (50 % aq., 791 µL) at room temperature. After 2 h stirring to r.t., the reaction mixture was quenched with aqueous Na_2_S_2_O_3_ sat. (25 mL) and extracted with EtOAc (4 x 15 mL). The combined organic extracts were dried over Na_2_SO_4_, filtered and the solvents were removed under vacuum to give the crude compound **5** (632.0 mg, 2.46 mmol) which was used without purification. ^1^H NMR (CDCl_3_, 300 MHz): δ_H_ (ppm) = 3.96-3.89 (1H, m); 3.94 (4H, s); 3.73-3.70 (1H, m); 3.49 (1H, dd, *J* = 7.0, 11.0 Hz); 2.40 (1H, dd, *J* = 9.5, 14.5 Hz); 2.29-2.28 (2H, m); 2.20 (1H, dd, *J* = 4.5, 14.5 Hz); 2.08 (1H, br s); 2.04-2.02 (1H, m); 1.70 (3H, s); 1.69 (2H, s); 1.12 (3H, s); 1.10 (3H, s).

**Compound 6: 2-(7,9,9-Trimethyl-1,4-dioxaspiro[4.5]dec-7-en-8-yl)acetaldehyde.** To a solution of the above crude compound **5** (632 mg, 2.46 mmol) in Et_2_O (12 mL) and H_2_O (6 mL) was added NaIO_4_ (633.6 mg, 2.96 mmol) at 0°C and stirred for 3 h to this temperature. Then, the reaction mixture was diluted with H_2_O (30 mL) and extracted with EtOAc (4 x 15 mL). The combined organic layers were washed with brine (2 x 15 mL), dried over Na_2_SO_4_, filtered, and concentrated under vacuum. The obtained residue was purified by column chromatography using silica gel and heptane/EtOAc 90:10 as eluent. Compound **6** (374.0 mg, 1.67 mmol, 73 % from compound **4**) was obtained as a colorless oil which solidified upon. ^1^H NMR (CDCl_3_, 300 MHz): δ_H_ (ppm) = 9.54 (1H, t, *J* = 2.5 Hz); 3.96 (4H, s); 3.14 (2H, s); 2.32 (2H, d, *J* = 1.0 Hz); 1.72 (2H, s); 1.62 (3H, s); 1.06 (6H, s). These data were in accordance with Yamamoto et al. (2020).

**Dimethyl (*E*)-2-(2-(7,9,9-trimethyl-1,4-dioxaspiro[4.5]dec-7-en-8-yl)vinyl)malonate (7).** To a suspension of NaH (60% dispersion in mineral oil, 275 mg, 6.9 mmol), in 1,4-dioxane (12 mL) was added dropwise dimethyl malonate (1.05 mL, 1.21 mg, 9.19 mmol) at r.t. and the reaction mixture was stirred for 20 min to this temperature. A solution of compound **6** (511.1 mg, 2.28 mmol) in 1,4-dioxane (1 mL) was then added at r.t. and the reaction mixture was heated under reflux (102 °C) overnight (16 h). After cooling at r.t., the reaction mixture was quenched with sat. aq. NH_4_Cl (20 mL), extracted with EtOAc (4 x 15 mL). The combined extract was successively washed with H_2_O (2 x 15 mL) and brine (2 x 15 mL), dried over Na_2_SO_4_, filtered, and concentrated under vacuum. The crude mixture was purified by silica gel column chromatography eluted with heptane/EtOAc 90:10 to give compound **7** (437.9 mg, 1.29 mmol, 57 %) as a colorless oil and the decarboxylated by-product **7-s** (40.5 mg, 0.14 mmol, 6 %). **7**: ^1^H NMR (CDCl_3_, 300 MHz): δ_H_ (ppm) = 6.08 (1H, br d, *J* = 15.9 Hz); 5.70 (1H, dd, *J* = 9.0, 15.9 Hz); 4.12 (1H, d, *J* = 9.0 Hz); 3.95 (4H, s); 3.75 (6H, s); 2.28 (2H, br s); 1.71 (3H, s); 1.68 (2H, s); 1.07 (6H, s). These data were in accordance with Yamamoto et al. (2020). **7s**: ^1^H NMR (CDCl_3_, 300 MHz): δ_H_ (ppm) = 5.96 (1H, dd, *J* = 1.0, 16.0 Hz); 5.55 (1H, tt, *J* = 7.0, 16.0 Hz); 3.95 (4H, s); 3.68 (3H, s); 3.14 (2H, dd, *J* = 1.5, 7.0); 2.28 (2H, br s); 1.71 (3H, d, *J* = 1.0 Hz); 1.68 (2H, br s); 1.07 (6H, s).

**Methyl (2*E*,3*E*)-2-(((4-methyl-5-oxo-2,5-dihydrofuran-2-yl)oxy)methylene)-4-(7,9,9-trimethyl-1,4-dioxaspiro[4.5]dec-7-en-8-yl)but-3-enoate (10).** Under N_2_ atmosphere and in anhydrous conditions, DIBAL (1.0 M in hexanes, 890 µL, 0.89 mmol) was added to a solution of compound **7** (101.8 mg, 0.30 mmol) at -78 °C. After stirring for 30 min at this temperature, the reaction mixture was quenched with MeOH (5 mL) and aqueous Rochelle salt sat. (5 mL), and allowed to warm to r.t. with stirring for 2 h. The organic layer was separated and the aqueous layer was extracted with EtOAc (3 x 5 mL). The combined organic layers were washed with aq. sat. Rochelle salt (2 x 5mL) then brine (2 x 5 mL), dried over Na_2_SO_4_, filtered and the solvent was removed under vacuum to give the corresponding enol-aldehyde mixture **8** (76.5 mg, ratio 50/50). This crude **8** was solubilized in THF (4 mL) then *t*-BuOK (51.6 mg, 0.34 mmol) and compound **9** (53.0 mg, 0.30 mmol) were successively added at 0 °C. After stirring for 2 h at 0 °C, the reaction mixture was quenched with sat. aq. NH_4_Cl (5 mL). The organic layer was separated and the aqueous layer was extracted with EtOAc (3 x 5 mL). The combined organic layers were successively washed with water (5 mL) and brine (2 x 5 mL), dried over Na_2_SO_4_, filtered, and concentrated under vacuum. The residue was purified by silica gel column chromatography eluted with heptane/EtOAc 84:16 to give compound **10** (10.6 mg, 0.026 mmol, 9 % from compound **7**) as a pale-yellow oil. ^1^H NMR (CDCl_3_, 300 MHz): δ_H_ (ppm) = 7.51 (1H, s); 6.94 (1H, t, *J* = 1.5 Hz); 6.79 (1H, br d, *J* = 16.0 Hz); 6.15 (1H, d, *J* = 16.5 Hz); 6.13 (1H, t, *J* = 1.5 Hz); 3.96 (4H, s); 3.76 (3H, s); 2.31 (2H, br s); 2.02 (3H, t, *J* = 1.5 Hz); 1.74 (3H, br s); 1.70 (2H, s); 1.09 (6H, s). These data were in accordance with Yamamoto et al. (2020).

**(±)-Heliolactone and (±)-6-*epi*-heliolactone (11).** Under N_2_ atmosphere and in anhydrous condition, *tert*-butyldimethylsilyl trifluoromethanesulfonate (TBDMS triflate) (8 µL, 9.21 mg, 0.034 mmol) was added to a stirred solution of compound **10** (10.6 mg, 0.26 mmol) in CH_2_Cl_2_ (700 µL) at 0 °C. After stirring at 0 °C for 30 min, the reaction mixture was quenched with sat. aq. NaHCO_3_ (1 mL), then the organic layer was separated, and the aqueous layer was extracted with EtOAc (3 x 1 mL). The combined organic layers were washed with H_2_O (1mL) then brine (2 x 1 mL), dried over Na_2_SO_4_, filtered, and concentrated under vacuum. The crude product was purified by rapid filtration over silica gel using EtOAc to give compound **11** (10.3 mg, 0.029 mmol, 97 %) as a mixture of racemic diastereomers. ^1^H NMR (CDCl_3_, 300 MHz): δ_H_ (ppm) = 7.53 (1H, br d, *J* = 1.0 Hz); 6.95 (1H, br s); 6.24 (1H, s); 6.15 (1H, br d, *J* = 1.0 Hz); 5.90 (1H, br s); 3.75 (3H, s); 2.57 (2H, br d, *J* = 7.5 Hz); 2.35 (1H, dd, *J* = 2.8, 16.5 Hz); 2.09 (1H, d, *J* = 17.0 Hz); 2.03 (3H, s); 1.90 (3H, s); 1.25 (1H, br s); 1.03 (3H, s); 0.96 (3H, d, *J* = 4.0 Hz). Data were in accordance with Woo and McErlean (2019).

The isomers of heliolactone were separated by chiral SFC (equipped with PDA) on an IC column using *n*-heptane/isopropanol (70:30, v/v) as an isocratic mobile phase in a flow of 1 mL/min (50-mL injections, 60-min runs). The above-synthesized compound **11** (10 mg) was solubilized in acetonitrile then isopropanol was added to obtain the final ratio of 1:6 (v/v) and the final concentration of 4 mg/mL. Fractions were collected manually and four peaks (1-4) were obtained at RT = 32.5, 35.7, 42.5, 53.1 min.

(−)-heliolactone [6*S*,11*R*] (1.8 mg), RT = 32.5 min; [α]_D_^20^ -30.0 (*c* 0.18, CH_2_Cl_2_)

(+)-6-*epi*-heliolactone [6*R*,11*R*] (1.9 mg), RT = 35.7 min; [α]_D_^20^ +89.8 (*c* 0.19, CH_2_Cl_2_)

(−)-6-*epi*-helilactone [6*S*,11*S*] (2.0 mg), RT = 42.5 min; [α]_D_^20^ -116.7 (*c* 0.20, CH_2_Cl_2_)

(+)-heliolactone [6*R*,11*S*] (2.1 mg), RT = 53.1 min; [α]_D_^20^ +51.2 (*c* 0.19, CH_2_Cl_2_)

**PCR, cloning and sequencing.** Conditioned seeds were dried on an 80 µm filter and then snap-frozen in liquid nitrogen. Seeds were then crushed into powder and RNA was extracted using the Nucleospin RNA Plant kit (Machery-Nagel). cDNA were synthesized with the qScript cDNA SuperMix (Quantabio) and then used as a template to amplify *OcuKAI2 and OcuD14* coding sequences by PCR, using specific primers (Supplemental Table 3) and high-fidelity DNA Polymerase (Q5 from NEB). Sequences were then inserted in pGEM®-T Easy Vector Systems from Promega® and confirmed by sequencing (Eurofins Genomics).

**RNAseq experiments.** For expression analysis following GS treatments, three independent batches of conditioned seeds were treated during 2 or 12 h either with mock (control) or with 1 µM (±)-GR24 or (−)-DCL. RNA were extracted following the previously-mentioned method. RNAseq experiment and analysis were performed by the Helixio genomic-bioinformatic platform, using the annotation of the OcIN23 population genome as template. Sequencing was performed as a 150 base pair double read utilizing the Illumina sequencer Element AVITI.

For expression analysis during the *O. cumana* life cycle, conditioned seeds were treated with 1 µM (±)-GR24 for 24 hours. Depending on the stage of development, the parasitic material was removed with fine forceps or excised from the sunflower roots with a razor blade. For each development stage and/or organ, samples were collected in triplicates of independent experiments for mRNA sequencing, constituting sixty samples. Thus, sixty independent plant material RNA-seq libraries were constructed using the TruSeq Stranded mRNA Kit (Illumina Inc., San Diego, CA, USA) according to the manufacturer’s protocol. Next-generation sequencing (NGS) was performed by the Genome & Transcriptome core facility of Genotoul (Toulouse, France) using the Illumina HiSeq3000 platform to produce 2x150 bp paired-end reads. For more information concerning the development stages from which biological samples were collected see Supplemental Table 2.

All 75 pairs of raw paired-end RNA reads were quality-trimmed using Trimmomatic v0.39 at default settings (ILLUMINACLIP:TruSeq3-PE.fa:2:30:10 LEADING:3 TRAILING:3 SLIDINGWINDOW:4:15 MINLEN:36) (Bolger *et al.*, 2014). Read quality before and after trimming was assessed using FastQC v0.11.9 (https://www.bioinformatics.babraham.ac.uk/projects/fastqc/). All pairs of trimmed reads were mapped against the reference genome of *Orobanche cumana* using STAR v2.7.11b (Dobin *et al.*, 2013). Read counts were normalized across all samples using the TMM (trimmed mean of M values) method (Robinson and Oshlack, 2010). Statistical analysis was performed by using a one-way ANOVA followed by a Dunnett’s post hoc test to compare treatments with the untreated group (Figure 2c).

**Protein expression and purifications.** AtD14 and AtKAI2 were purified and expressed with cleavable GST tags as described (de Saint Germain et al., 2016). For OcuKAI2d expression, the coding sequences from *O. cumana* were amplified by PCR by means of a seed-derived cDNA template and specific primers (Supplementary Table 3) containing a protease cleavage site for tag removal, and subsequently cloned into the pGEXT-4T-3 expression vector. The OcuKAI2d and OcuKAI2d^S96A^ proteins were purified and expressed as described (de Saint Germain et al., 2016). But the protease inhibitor has not been added in the buffer in order to avoid non-specific covalent adducts to serine of the catalytic triad (Figure S8).

**Site-directed mutagenesis**. Site-directed mutagenesis experiments were done with the QuickChange II XL Site Directed Mutagenesis kit (Stratagene) on pGEX-4T-3-OcuKAI2d plasmids (Supplementary Table 3). Mutagenesis was verified by systematic DNA sequencing.

**Enzymatic hydrolysis of GR24 isomers by purified proteins.** Ligands (10 µM) were incubated without and with purified proteins (5 µM) for 150 min at 25 ºC in PBS (0.1 mL, pH 6.8) in presence of (±)-1-indanol (100 µM) as the internal standard. The solutions were acidified to pH 1 with 10% trifluoroacetic acid in CH_3_CN (v/v) (2 µL) to quench the reaction and centrifuged (12 min, 12,000 tr/min). Thereafter, the samples were subjected to RP-UPLC-MS analyses using Ultra Performance Liquid Chromatography system equipped with a PDA and a Triple Quadrupole mass spectrometer Detector (Acquity UPLC-TQD, Waters, USA). RP-UPLC (HSS C_18_ column, 1.8 μm, 2.1 mm × 50 mm) with 0.1% formic acid in CH_3_CN and 0.1% formic acid in water (aq. FA, 0.1%, v/v, pH 2.8) as eluents [10% CH_3_CN, followed by linear gradient from 10 to 100% of CH_3_CN (4 min)] was carried out at a flow rate of 0.6 mL/min. The detection was performed by PDA using the TQD mass spectrometer operated in Electrospray ionization positive mode at 3.2 kV capillary voltage. The cone voltage and collision energy were optimized to maximize the signal and were respectively 20 V for cone voltage and 12 eV for collision energy and the collision gas used was argon at a pressure maintained near 4.5.10^-3^ mBar.

**Enzymatic assays with profluorescent probes.** The enzyme activity was deter­mined by measuring the release of the fluorescent intensities of each fluorophores resulting from the cleavage of profluorescent probes by AtD14, AtKAI2 and OcuKAI2 proteins in a Tecan SPARK M10 in a 96-well format (de Saint Germain *et al.*, 2021). In the assay, using an Integra Viaflo 96 robot, 50 μL of a solution of protein at 0.33 µM in the same buffer was added simultaneously in all 96 wells to 50 μL profluo­rescent substrate solution (at varying concentrations, prepared from a 10 mM stock solution in 100% DMSO) in PBS (100 mM phosphate, pH 6.8, 150 mM NaCl). After 15 s lag time, the formation of fluorophores was recorded over 3 hours at 15-s intervals at 25 °C. Each fluorophore was analyzed with the following excitation (ex) and emission (em) wavelengths: DiFMU λ_ex_ 360 nm/λ_em_ 450 nm. All experiments were repeated with three technical replicates. The fluorescence of each fluorophore was also determined for each measurement at the same time frame but in absence of enzymes in order to determine the standard curves.

**nanoDSF.** Denaturation profiles of the OcuKAI2d protein samples were obtained using the Tycho NT.6 instrument (Nanotemper, München, Germany). Proteins were diluted in PBS (100 mM Phosphate, pH 6.8, 150 mM NaCl) to a concentration of 10 μM. Ligands were tested at the concentration of 200 or 400 µM. 10 μL samples were loaded into NT.6 capillaries and heated from 35 °C to 95 °C, at a rate of 0.3 K/s. Six samples were analyzed simultaneously. Raw data were exported into datasets that contained fluorescence intensity at 330 and 350 nm (F_330_ and F_350_), the ratio of these values (F_350_/F_330_), and their first derivatives (∂F_330_/∂T, ∂F_350_/∂T, ∂(F_350_/F_330_)/∂T). The plots show one of the three independent data collections done for each protein.

**Direct electrospray ionization – mass spectrometry of OcuKAI2 proteins.** Mass spectrometry measurements were performed with an electrospray Triple-TOF 4600 mass spectrometer (ABSciex) coupled to the nanoRSLC ultra performance liquid chromatography system (Thermo Scientific) equipped with a C4-desalting column. For ESI−MS measurements, the instrument was operated in positive and RF quadrupole modes with the TOF data being collected between *m/z* 400−2990. Collision energy was set to 10 eV and nitrogen was used as collision gas. Mass spectra acquisition was performed after loading and desalting of protein samples on C4-column. The Analyst and Peakview softwares were used for acquisition and data processing, respectively. Mass spectra were deconvoluted with the MaxEnt algorithm. The protein average masses are calculated from the spectra with a mass accuracy of ± 1 Da.

**NanoLC-MSMS peptide analysis of OcuKAI2 proteins.** Protein samples were digested before submission to mass spectrometry analysis. Trypsin-generated peptides from OcuKAI2d proteins were analyzed by nanoLC-MS/MS using a nanoElute liquid chromatography system (Bruker) coupled to a timsTOF Pro mass spectrometer (Bruker). Peptides were loaded with solvent A on a trap column (nanoEase C18, 100 Å, 5 µm, 180 µm x 20 mm) and separated on a Aurora analytical column (ION OPTIK, 25 cm x 75 µm, C18, 1.6 µm) with a gradient of 0-35% of solvent B for 30 min. Solvent A was 0.1 % formic acid and 2% acetonitrile in water and solvent B was acetonitrile with 0.1% formic acid. MS and MS/MS spectra were recorded from *m/z* 100 to 1700 with a mobility scan range from 0.6 to 1.4 V.s/cm^2^. MS/MS spectra were acquired with the PASEF (Parallel Accumulation Serial Fragmentation) ion mobility-based acquisition mode using a number of PASEF MS/MS scans set as 10. MS and MS/MS raw data were processed and converted into mgf files with DataAnalysis software (Bruker).

Peptides identifications were performed using the MASCOT search engine (Matrix science, London, UK) against OcuKAI2d sequences. Database searches were performed using trypsin cleavage specificity with two possible missed cleavages. Ligand mass adducts of 96.02 Da (GR24, heliolactone) and 230.13 Da (DCL) on serine/histidine and oxidation of methionines were set as variable modifications. Peptide and fragment tolerances were set at 10 ppm and 0.05 Da, respectively. Only ions with a score higher than the identity threshold and a false-positive discovery rate of less than 1% were considered.

**References**:

**Bolger, A.M., Lohse, M. and Usadel, B.** (2014) Trimmomatic: a flexible trimmer for Illumina sequence data. *Bioinformatics*, **30**, 2114-2120. <https://doi.org/10.1093/bioinformatics/btu170>

**de Saint Germain, A., Clavé, G., Badet-Denisot, M.A., Pillot, J.P., Cornu, D., Le Caer, J.P., Burger, M., Pelissier, F., Retailleau, P., Turnbull, C., Bonhomme, S., Chory, J., Rameau, C. and Boyer, F.D.** (2016) An histidine covalent receptor and butenolide complex mediates strigolactone perception. *Nat. Chem. Biol.*, **12**, 787-794. <https://doi.org/10.1038/nchembio.2147>

**de Saint Germain, A., Clavé, G. and Boyer, F.-D.** (2021) Synthesis of Profluorescent Strigolactone Probes for Biochemical Studies. In *Strigolactones: Methods and Protocols* (Prandi, C. and Cardinale, F. eds). New York, NY: Springer US, pp. 219-231.

**de Saint Germain, A., Retailleau, P., Norsikian, S., Servajean, V., Pelissier, F., Steinmetz, V., Pillot, J.-P., Rochange, S., Pouvreau, J.-B. and Boyer, F.-D.** (2019) Contalactone, a contaminant formed during chemical synthesis of the strigolactone reference GR24 is also a strigolactone mimic. *Phytochemistry*, **168**, 112112. <https://doi.org/10.1016/j.phytochem.2019.112112>

**Dobin, A., Davis, C.A., Schlesinger, F., Drenkow, J., Zaleski, C., Jha, S., Batut, P., Chaisson, M. and Gingeras, T.R.** (2013) STAR: ultrafast universal RNA-seq aligner. *Bioinformatics*, **29**, 15-21. <https://doi.org/10.1093/bioinformatics/bts635>

**Robinson, M.D. and Oshlack, A.** (2010) A scaling normalization method for differential expression analysis of RNA-seq data. *Genome Biol.*, **11**, R25. <https://doi.org/10.1186/gb-2010-11-3-r25>

**Woo, S. and McErlean, C.S.P.** (2019) Total Synthesis and Stereochemical Confirmation of Heliolactone. *Org Lett.*, **21**, 4215-4218. <https://doi.org/10.1021/acs.orglett.9b01402>

**Yamamoto, S., Atarashi, T., Kuse, M., Sugimoto, Y. and Takikawa, H.** (2020) Concise synthesis of heliolactone, a non-canonical strigolactone isolated from sunflower. *Biosci. Biotechnol. Biochem.*, **84**, 1113-1118. <https://doi.org/10.1080/09168451.2020.1734444>

**Yoshimura, M., Fonné-Pfister, R., Screpanti, C., Hermann, K., Rendine, S., Dieckmann, M., Quinodoz, P. and De Mesmaeker, A.** (2019) Total Synthesis and Biological Evaluation of Heliolactone. *Helv. Chim. Acta*, **102**, e1900211. <https://doi.org/10.1002/hlca.201900211>

**Yoshino, T., Ng, F. and Danishefsky, S.J.** (2006) A Total Synthesis of Xestodecalactone A and Proof of Its Absolute Stereochemistry:  Interesting Observations on Dienophilic Control with 1,3-Disubstituted Nonequivalent Allenes. *J. Am. Chem. Soc.*, **128**, 14185-14191. <https://doi.org/10.1021/ja064270e>
